# Comparative perioperative and oncological outcomes of the da Vinci Xi versus Si robotic surgical systems in rectal cancer surgery: a systematic review and meta-analysis

**DOI:** 10.1007/s11701-026-04059-0

**Published:** 2026-10-20

**Authors:** Ivens Murad Garcia Magalhães, Fillipe Augusto de Oliveira Paixão, Gustavo Tamura, Pedro Averbach

**Affiliations:** 1 Faculdade de Medicina, Universidade Federal de Juiz de Fora, Avenida Presidente Itamar Franco, Juiz de Fora, Minas Gerais Brazil; 2 Hospital Governador Celso Ramos, Florianópolis, Santa Catarina Brazil; 3 Hospital Sírio-Libanês, São Paulo, Brazil; 4 Discipline of Surgical Technique and Experimental Surgery, Faculdade de Medicina, Universidade de São Paulo, São Paulo, Brazil

**Keywords:** Rectal cancer, Robotic surgical procedures, da Vinci Xi, da Vinci Si, Total mesorectal excision, Meta-analysis

## Abstract

The da Vinci Xi replaced the Si with a boom-mounted architecture intended to remove the re-docking and multiquadrant limits on pelvic access. Whether it changes outcomes in rectal cancer surgery is unresolved: existing syntheses pooled rectal with colonic resection. Five databases, Google Scholar and citation graphs were searched to August 2026, without language or design restriction, for comparative studies reporting outcomes separately by platform in rectal cancer resection. Screening, extraction and risk-of-bias assessment were done in duplicate. Random-effects models with a Hartung–Knapp adjustment were used; certainty was rated with GRADE. Eight non-randomised cohorts (843 patients; 380 Xi, 463 Si) were included. Pooling all five cohorts reporting operative time, the mean difference was − 27.7 min (95% confidence interval − 80.4 to + 25.0; I² 95%); restricted to the four reporting means with standard deviations, thus conditional on excluding the largest cohort, it was − 50.6 min (− 69.4 to − 31.7). Docking time was shorter with the Xi in all three reporting cohorts, which defined it differently or not at all. The evidence is very uncertain about the effect of platform on anastomotic leak (risk ratio 0.53, 0.24 to 1.14), complications, conversion, reoperation, margin involvement and specimen quality; certainty was very low throughout. The evidence is very uncertain about the effect of the da Vinci Xi compared with the Si on perioperative and oncological outcomes in rectal cancer, and functional outcomes have not been compared. Because the platforms were compared across periods, centres and levels of experience, no estimate isolates the platform.

**PROSPERO registration** CRD420261480007, registered 18 August 2026, before data extraction.

**Supplementary Information:**

The online version contains supplementary material available at https://doi.org/10.1007/s11701-026-04059-0.

## Introduction

Total mesorectal excision determines oncological outcome in rectal cancer [1], and it is performed in an anatomical space that punishes poor exposure: the pelvis is narrow and fixed, the dissection plane lies millimetres from the autonomic nerves and the mesorectal fascia, and adequate mobilisation usually demands work in a second abdominal quadrant. Robot-assisted surgery was adopted here because stable three-dimensional vision, wristed instruments and tremor filtration are most valuable where access is constrained [2].

The third-generation da Vinci Si, introduced in 2009, retained a patient-side cart whose position fixed the operative field once docking was complete, so working in the splenic flexure and then in the pelvis required re-docking, repositioning the arms, or completing part of the operation laparoscopically. The fourth-generation da Vinci Xi, released in 2014, was designed to remove that constraint: its overhead boom rotates to address any quadrant without moving the cart, the arms are thinner and have greater reach, and the endoscope can be mounted on any arm [3]. Rectal resection, combining splenic flexure mobilisation with pelvic dissection, typifies the multiquadrant surgery this redesign addressed.

Two meta-analyses have compared the platforms in colorectal surgery, pooling six studies and 610 patients [4] and eight studies and 1000 patients [5]. Neither restricted its population to rectal cancer or analysed rectal-specific results separately; both included colonic or benign resections, one cohort consisting of right hemicolectomies alone. Of the pathological and oncological outcomes, the 2025 analysis pooled only lymph node yield and the 2021 analysis none; neither reported margin involvement, specimen quality, R0 resection, recurrence, survival or functional outcomes, precisely the outcomes that matter in rectal cancer, where the constraints the Xi was built to relieve are pelvic. And because the Xi was usually introduced after the Si, and sometimes at different centres, the two groups were operated in different periods or places by surgeons at different points on their learning curve. We therefore conducted a systematic review and meta-analysis restricted to rectal cancer, treating this confounding as a structural feature of the evidence.

## Methods

### Protocol and registration

The review followed the PRISMA 2020 statement [6] and was registered in PROSPERO (CRD420261480007) on 18 August 2026, after the database searches and title-and-abstract screening (15–16 August) but before data extraction. A full protocol was written before data extraction. Deviations are listed in Online Resource 1 (Section S7): one cohort was excluded after data extraction, when its tumour-location data showed that the registered population rule could not be met (Section S5), and several analyses were changed in response to peer review, each labelled post hoc with its pre-specified counterpart reported alongside.

### Literature search

We searched PubMed/MEDLINE, Embase, Scopus, the Web of Science Core Collection and the Cochrane Central Register of Controlled Trials from inception, with no date, language, publication-type or study-design restriction, on 15 August 2026 (Embase, Scopus) and 16 August 2026 (the remainder). Strategies combined three concept blocks with AND — rectal cancer, robotic surgery and platform-specific terms — using controlled vocabulary with title, abstract and keyword terms and, where supported, proximity operators requiring the ambiguous strings “Xi” and “Si” to occur adjacent to a robot, platform, system or generation term.

As pre-specified in the protocol, Google Scholar was searched as a supplementary source on 20–21 August 2026; the protocol specified the first 100 to 200 records of each strategy, and the first 100 were screened. We also screened the reference lists of five reports and the citation graphs of six seed publications; five included cohorts could not be forward-searched individually, so the citation searching is not claimed to be comprehensive. Trial registers were not searched. Full strategies are in Online Resource 1.

### Eligibility criteria

We included comparative studies reporting at least one outcome separately for the da Vinci Xi and the da Vinci Si in adults undergoing resection for rectal cancer; conference abstracts and non-English publications were eligible. Two rules were applied strictly: a mixed colorectal study was eligible only where an outcome was reported with a rectal-specific denominator, and a study that spanned a platform transition but pooled the two platforms was not a comparative study for this review, however large. Comparisons against other platforms, laparoscopy or open surgery were excluded unless a direct Xi versus Si contrast was separately extractable.

### Study selection and data extraction

Titles, abstracts and then full texts were screened in duplicate, disagreements were resolved by a third reviewer, and a fourth re-checked every extracted value and risk-of-bias judgement against the source. The unit of synthesis was the independent cohort, not the publication: multiple reports of one cohort were linked and counted once, with an index publication pre-specified for each so that no patient could contribute twice. Data were extracted onto a piloted standardised form and each value obtained by arithmetic rather than printed directly was flagged as derived. For anastomotic leak the denominator was restricted to patients who received an anastomosis. Outcome definitions and the period and centre of each platform arm were tabulated from the sources (Online Resource 1, Supplementary Tables 6 and 7).

Corresponding authors were contacted where a publication contained an unresolved internal inconsistency affecting an extracted value, where a critical outcome appeared to have been ascertained but not reported, or where a continuous outcome lacked a measure of dispersion. One author replied, confirming the extracted values and supplying counts the publication does not print; these are identified as author-supplied in Online Resource 1.

### Risk of bias and certainty of evidence

Non-randomised studies were assessed with ROBINS-I V2 [7, 8] against a specified target trial in which patients would be randomly assigned to platform by the same surgical team in the same calendar period. Assessment was result-specific. Pre-specified confounding domains were calendar period, surgeon and team experience, institution, tumour height and stage, neoadjuvant therapy, procedure type, body mass index, sex and perioperative care protocol. Missing information was not interpreted as low risk of bias. Following the guidance that results at critical risk of bias should generally be excluded from synthesis [7, 9], the primary analysis of each affected outcome excludes them, and analyses retaining them are pre-specified sensitivity analyses.

Certainty of evidence was rated with GRADE [10, 11]. Because every study was assessed with ROBINS-I, certainty started at high and was rated down two levels for serious and three for very serious risk of bias [11]; inconsistency, indirectness, imprecision, judged against both the interval and the number of events, and publication bias were judged for every outcome by two reviewers independently, with a third arbitrating. A large language model was used only to translate draft material into English and to format the manuscript; it generated no scientific content and performed no part of the review.

### Statistical analysis

Analyses used R with the meta [12] and metafor [13] packages, following the Cochrane Handbook [9]; every figure, the flow diagram included, was produced by the script provided as Online Resource 4. All models were random-effects with a restricted maximum-likelihood estimate of between-study variance (τ²) and a Hartung–Knapp confidence interval [14, 15], with prediction intervals [16]. Dichotomous outcomes were pooled as risk ratios from study-level log risk ratios, to which the software adds 0.5 in studies with a zero cell; studies with no event in either arm are excluded, and the Mantel–Haenszel estimate [17] without correction is given in Online Resource 4. Continuous outcomes were pooled as mean differences.

Medians with interquartile ranges were converted to means and standard deviations [18, 19]; nothing was imputed. The protocol restricted primary analyses to cohorts reporting a mean with a standard deviation. Because that rule excluded the largest cohort, about two-fifths of the patients in the cohorts reporting these outcomes and the only one in which the Xi was slower, the analysis including converted values is now primary for operative time, length of stay and lymph node yield, a post hoc change made in response to peer review; the pre-specified analysis is reported alongside, labelled as conditional on excluding that cohort. An outcome was pooled only where at least three cohorts contributed comparable data. Pre-specified sensitivity analyses comprised leave-one-out analysis, the Peto odds ratio, reported rather than derived counts and alternative outcome definitions.

Because the Hartung–Knapp interval can be narrower than a conventional interval when τ² is estimated as zero, as it was for every dichotomous outcome, intervals with the ad hoc variance correction are reported alongside and used for interpretation where the two differ [9]. With three to five cohorts I² is imprecise and is reported with its confidence interval; an I² of 0% was not read as consistency. Where three or fewer cohorts were informative, conversion and reoperation are summarised by event counts and the risk difference, which retains every reporting cohort, with ratio estimates and binomial-normal mixed models [13] as sensitivity analyses. For mesorectal specimen quality, complete in every patient in two cohorts, the risk difference is primary. Docking time, defined differently or not at all, is presented study by study with the direction of effect counted [9]. These three choices were made post hoc, after peer review.

## Results

### Study selection

**Fig. 1:**
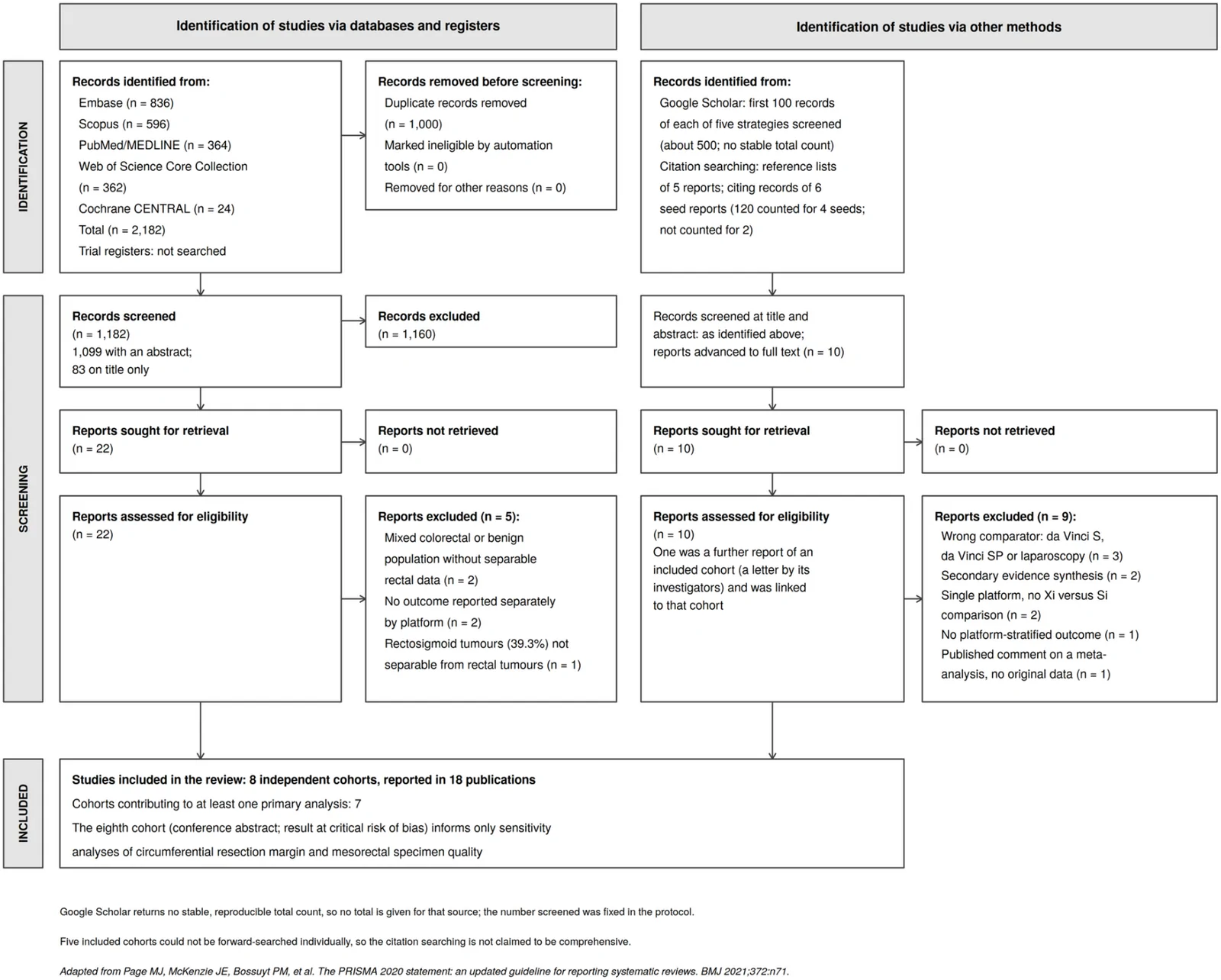
PRISMA 2020 flow diagram. The supplementary Google Scholar search and the citation searching (20–21 August 2026) are shown as identification via other methods; Google Scholar gives no stable total count, so the number screened, fixed in the protocol, is shown instead. Trial registers were not searched. One of the ten reports assessed through other methods is a letter by the investigators of an included cohort; it is counted among the eighteen reports of included studies and linked to that cohort, whereas the excluded published comment is a commentary on a previous meta-analysis. Seven cohorts contribute to at least one primary analysis; the eighth informs only sensitivity analyses. Adapted from Page et al. [6]

The searches returned 2,182 records, of which 1,000 were duplicates. Of 1,182 unique records screened, 1,160 were excluded and 22 reports were assessed at full text, five of which were excluded. One of these was a comparative cohort that would otherwise have been eligible but in which 39.3% of patients had rectosigmoid tumours and no outcome was reported with those patients removed [20]. The supplementary search and citation searching yielded ten further reports, nine excluded and one an additional report of an included cohort; no new eligible study was found. Eight independent cohorts [21–28], reported in eighteen publications [29–38], were included (Fig. 1). Seven contribute to at least one primary analysis; the eighth, a conference abstract at critical risk of bias, informs only sensitivity analyses.

### Characteristics of included studies

The eight cohorts comprised 843 patients, 380 operated with the da Vinci Xi and 463 with the da Vinci Si (Table 1). All were non-randomised: seven retrospective comparative cohorts and one retrospective 1:1 case-matched cohort, recruited between 2010 and 2024 in Turkey, Italy, the United Kingdom and Portugal, the United States, Hong Kong, Taiwan and China. One cohort was available only as a conference abstract.

**Table 1:** Study, patient and tumour characteristics of the eight included cohorts. In every cell the da Vinci Xi value is given first and the da Vinci Si value second

Study (year)	Country	Design; surgeon(s)	Recruitment; timing of the two arms	Xi/Si (*n*)	BMI, kg/m²	Tumour height	Tumour stage	ASA	Neoadjuvant therapy
Ozben (2016) [21]	Turkey	Retrospective; multivariable; single institution, several surgeons	Jan 2010–Mar 2015; sequential, same institution (Xi from Nov 2014)	28/25	27.0 ± 3.8 vs. 26.6 ± 4.1	9.5 ± 4.7 vs. 12.0 ± 4.4 cm from the anal verge (*p* = 0.09)	(y) pTNM III–IV 13 (46.4%) vs. 3 (12.0%); *p* = 0.02	I/II/III: 8/16/4 vs. 12/12/1 (*p* = 0.21)	Chemoradiotherapy 13 (46.4%) vs. 12 (48.0%)
Cha/Marks (2018) [22]	USA	Retrospective, prospectively maintained database	2011–2017; timing not reported	36/55	NR	NR	NR	NR	NR
Morelli (2018) [23]	Italy	Retrospective; 1:1 case-matched on 8 variables; single surgeon	Apr 2010–Sep 2016; sequential, one surgeon (Xi from Jan 2015)	30/30	24.9 ± 3.5 vs. 24.4 ± 3.4 (*p* = 0.52)	6.4 ± 4.3 vs. 7.1 ± 4.1 cm from the anal verge (*p* = 0.26)	Stage I 18 (60%) vs. 15 (50%); II–III 12 vs. 15 (*p* = 0.44); clinical or pathological not stated	I/II/III/IV: 1/16/13/1 vs. 0/15/15/0	Radio-chemotherapy 12 (40%) vs. 15 (50%)
Tung (2018) [24]	Hong Kong SAR	Retrospective; three arms; single institution	Jan 2016–Apr 2018; timing not reported	24/33	NR	NR	pStage I/II/III: 5/11/8 vs. 10/9/14	NR	Unspecified 8 (33%) vs. 13 (39.4%)
Huang (2019) [25]	Taiwan	Retrospective; mixed colorectal; single surgical team	Dec 2011–Oct 2017; sequential, same team (Xi from May 2015)	29/77 (rectal)	23.4 ± 3.9 vs. 23.8 ± 4.0 (whole colorectal cohort)	NR	Clinical stage III–IV 33 (55.0%) vs. 91 (75.8%); *p* = 0.03 (whole colorectal cohort)	≥ III: 7 (11.7%) vs. 15 (12.5%) (whole colorectal cohort)	Chemoradiotherapy 22 (75.9%) vs. 66 (85.7%) (rectal subgroup)
Panteleimonitis (2020) [26]	UK; Portugal	Retrospective multicentre (3 centres), several surgeons	May 2013–May 2018; by centre: Si at one centre (to Feb 2016), Xi at two others (from Nov 2015 and May 2016)	116/124	28 (24–31) vs. 27 (24–30), median (IQR); *p* = 0.221	NR	pT3–4 63 (55.7%) vs. 56 (45.2%); *p* = 0.246; no N, M or stage group reported	I/II/III/IV: 7/84/20/1 vs. 10/93/15/0 (*p* = 0.467)	Radiotherapy only 36 (31.0%) vs. 30 (24.2%)
Aliyev (2022) [27]	Turkey	Retrospective; CUSUM; single surgeon	Jan 2011–May 2021; sequential, one surgeon, two hospitals (Xi from Jan 2018)	62/88	24.93 ± 1.31 vs. 24.76 ± 1.24 (*p* = 0.507)	Lower rectum 55 (88.7%) vs. 70 (79.5%); anatomical subsites, no centimetre definition (*p* = 0.071)	Clinical TNM III 25 (40.3%) vs. 18; Si counts sum to 44, not 88	NR (ASA not reported)	Long-course chemoradiotherapy 52 (83.9%) vs. 74 (84.1%)
Wang (2026) [28]	China	Retrospective; multivariable; Cox; single institution	Nov 2019–Jun 2024; Si alone to Dec 2020, then both platforms concurrently	55/31	> 25: 15 (27.3%) vs. 6 (19.4%); categories only (*p* = 0.415)	≤ 5 cm from the anal verge 21 (38.2%) vs. 9 (29.0%); dichotomised (*p* = 0.393)	Stage III–IV 21 (38.2%) vs. 11 (35.5%); *p* = 0.397	NR; ASA ≥ III was an exclusion criterion	Unspecified 5 (9.1%) vs. 6 (19.4%)

NR not reported, BMI body mass index, ASA American Society of Anesthesiologists physical status, CUSUM cumulative sum, IQR interquartile range. Continuous values are mean ± standard deviation unless stated otherwise. All eight cohorts are non-randomised. In four the da Vinci Xi replaced the da Vinci Si within the same team; in the multicentre cohort all Si patients were operated at one centre and all Xi patients at two others; in the most recent cohort both platforms were available concurrently after an initial period of Si use alone; two cohorts did not report the timing (Online Resource 1, Supplementary Table 7)

Caveats attaching to individual cells, none resolvable from the published record. Huang 2019 reports body mass index, ASA and stage only for its whole colorectal cohort (60 Xi, 120 Si); the only baseline field with a rectal-specific denominator is neoadjuvant therapy, and rectal cases made up 48.3% of its Xi arm against 64.2% of its Si arm. Aliyev 2022 reports clinical stage for only 44 of its 88 Si patients and computes the percentages against that reduced denominator, and gives the start of its Si series as January 2011 in its Methods and December 2011 in its Abstract. Panteleimonitis 2020 replaced missing values with the series mean (10 ASA, 3 T stage, 12 BMI) and its denominators are correspondingly smaller than the arm sizes without being labelled as such. Morelli 2018 ASA counts sum to 31 in an arm of 30, and its tumour stage is not identified as clinical or pathological. Tung 2018 compares the Xi with two separate Si arms, so the Si counts shown are our own sums; its published p values are three-group tests that do not apply to the two-group comparison and are not shown. Neoadjuvant therapy is not a single exposure across the review, and tumour height is not a common metric: only two cohorts report a continuous distance from the anal verge, so no pooled comparison of tumour height is possible

The timing of the two arms differed between cohorts (Table 1; Online Resource 1, Supplementary Table 7). In four cohorts the Xi replaced the Si within the same team, between November 2014 and January 2018. In the multicentre cohort the platform was determined by centre, all Si patients being operated at one centre and all Xi patients at two others, with periods overlapping between November 2015 and February 2016 [26]. In the most recent cohort both platforms were available concurrently after an initial 14 months of Si use alone, allocation following availability and theatre scheduling [28]. Two cohorts did not report the timing. Baseline characteristics are incompletely reported; stage differed significantly between arms in one cohort [21], and in two others on tests that are not directly comparable [24, 25]. The mixed colorectal cohort contributed margin involvement and, on its rectal procedures (28 Xi, 73 Si), operative time to one sensitivity analysis; another cohort reported means without dispersion and contributed only dichotomous data. Neither corresponding author replied to our query.

### Risk of bias

Twenty-three study results were assessed; twenty-one were at serious and two at critical risk of bias, and none was at low or moderate risk overall (Online Resource 2, Supplementary Fig. 1). Confounding dominated, structurally: platform was determined by date of surgery, by centre or by theatre availability, so calendar period, centre, surgeon experience and perioperative care are collinear with or entangled in the exposure, and neither the matching used by one cohort nor the regression used by two others addresses them. Selection of the reported result was at serious risk in seven results, each because of an internal inconsistency in the published paper.

### Operative efficiency

**Fig. 2:**
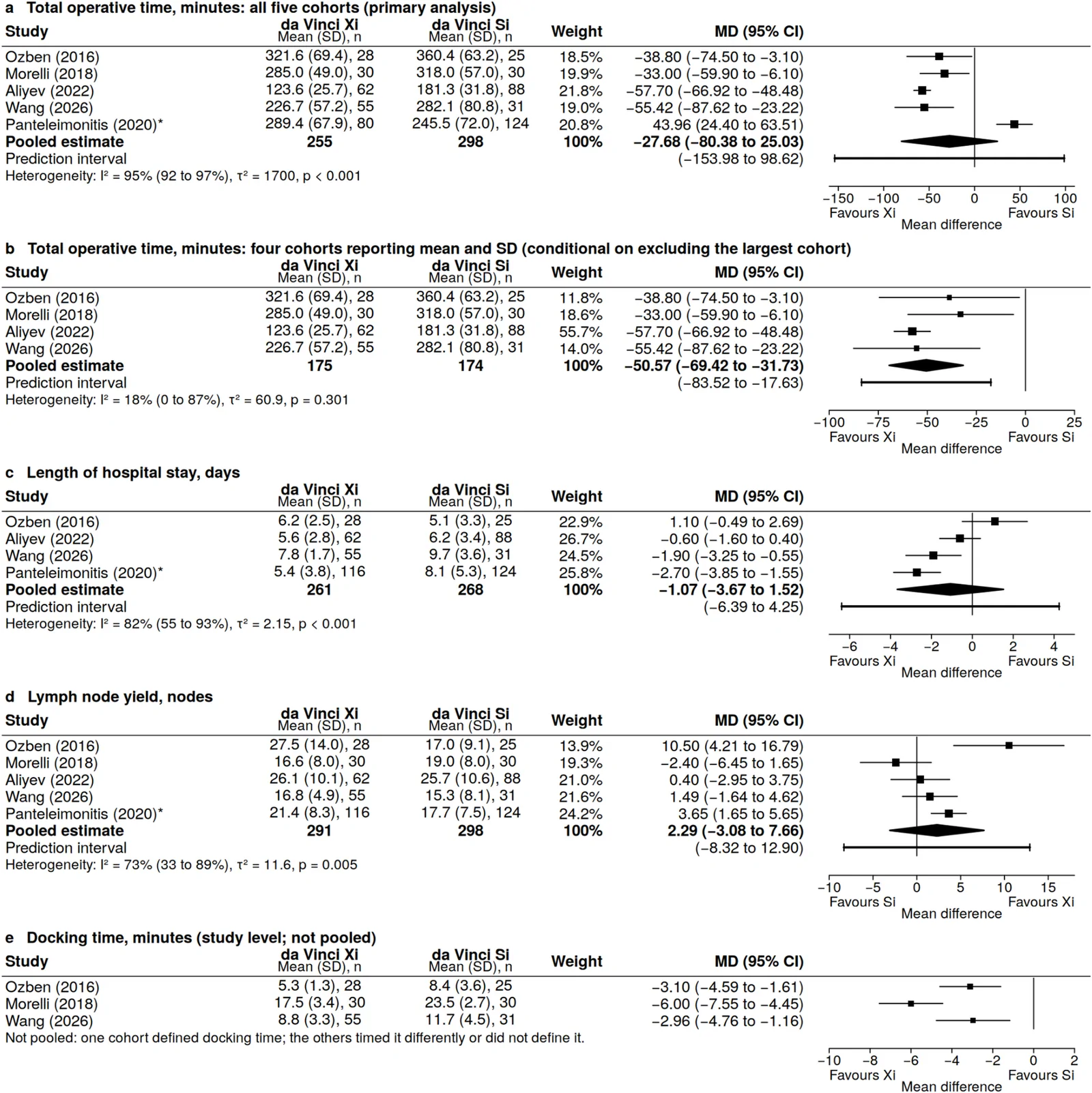
Forest plots for the continuous outcomes. (**a**) Total operative time, primary analysis of all five rectal cohorts; the largest cohort, marked with an asterisk, reported medians with interquartile ranges, converted to means and standard deviations [18, 19], and contributes the 80 Xi patients whose operative time was recorded. (**b**) Total operative time restricted to the four cohorts reporting a mean with a standard deviation; this estimate is conditional on excluding the largest cohort. (**c**) Length of hospital stay. (**d**) Lymph node yield. (**e**) Docking time, shown study by study without a pooled estimate because definitions differed or were not stated. An asterisk after a study label marks values converted from medians and interquartile ranges. Pooled models are random-effects with restricted maximum-likelihood estimation and a Hartung–Knapp adjustment; the diamond is the pooled mean difference and the bar beneath it the prediction interval. For operative time, docking time and length of stay a lower value favours the da Vinci Xi; for lymph node yield a higher value does, and the axis in panel **d** is oriented accordingly

With the largest cohort included, its medians converted, operative time was 27.7 min shorter with the Xi (95% CI − 80.4 to + 25.0; five cohorts, 553 patients; I² 95%, 95% CI 92 to 97%; prediction interval − 154.0 to + 98.6) (Fig. 2a). Restricted to the four cohorts reporting a mean with a standard deviation, and therefore conditional on excluding that cohort, it was 50.6 min shorter (− 69.4 to − 31.7; 349 patients; I² 18%, 0 to 87%) (Fig. 2b). The excluded cohort did not record operative time at one of its two Xi centres, so 80 of its 116 Xi patients contribute [26]. The cohort carrying most weight in the conditional analysis does not define its endpoint and labels the same variable console time in its regression analysis [27]; without it the estimates were − 19.4 (− 91.0 to + 52.1) and − 41.4 (− 70.8 to − 12.0) minutes.

Docking time was shorter with the Xi in all three cohorts reporting it, by 3.1, 6.0 and 3.0 min (Fig. 2e), but only one defined it, absolute times differed threefold, and some procedures required two dockings, so it was not pooled; three of three in one direction cannot exclude chance (sign test *P* = 0.25). Console time, reported by two cohorts, favoured the Xi in both and was not pooled. No subgroup analysis was possible (Supplementary Fig. 2).

### Perioperative safety

**Fig. 3:**
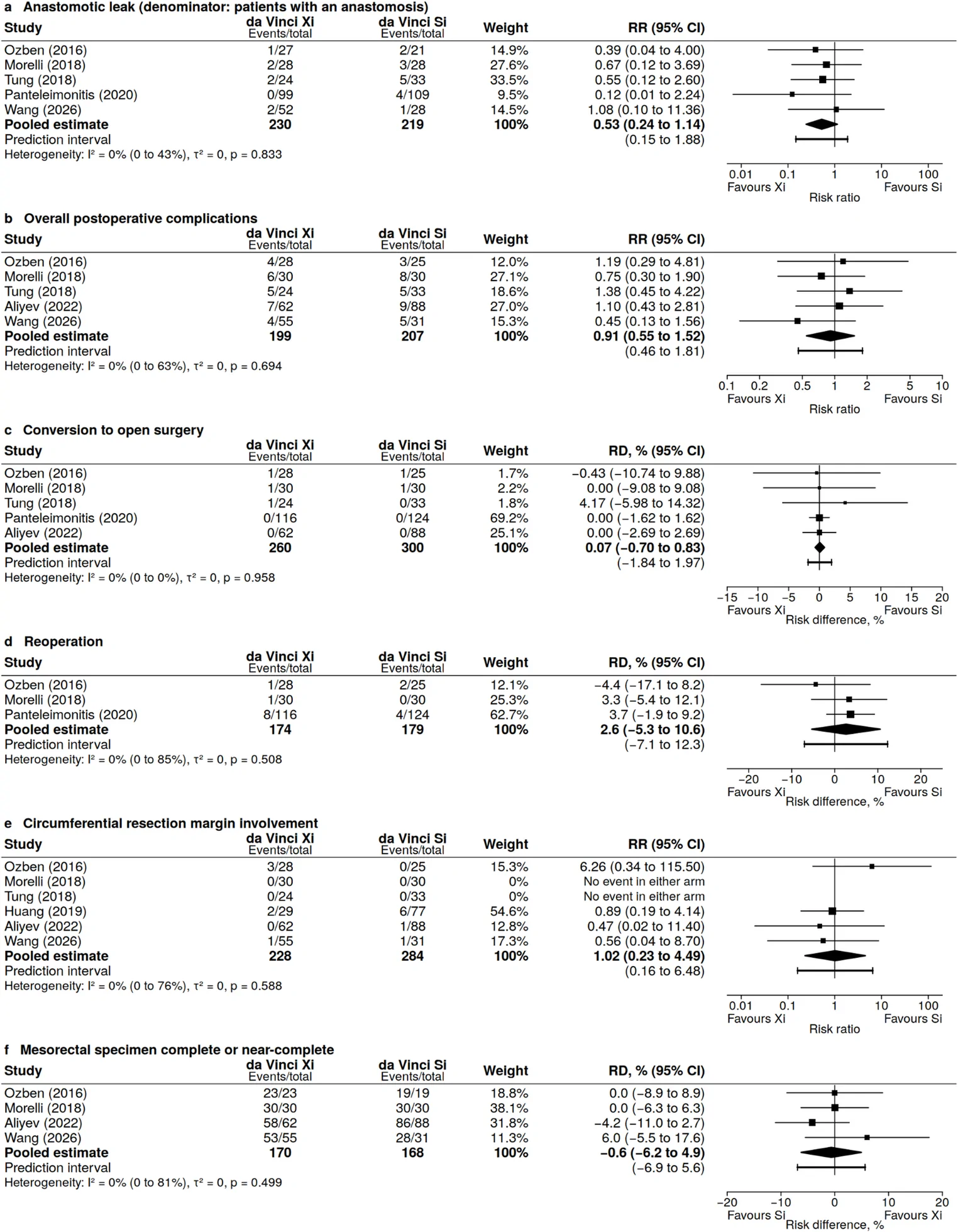
Forest plots for the dichotomous outcomes. (**a**) Anastomotic leak, with the number of patients who received an anastomosis as the denominator. (**b**) Overall postoperative complications. (**c**) Conversion to open surgery and (**d**) reoperation, shown as risk differences in percentage points, which retain cohorts with no event in either arm; ratio estimates are given in Table 2. (**e**) Circumferential resection margin involvement, risk ratio; cohorts with no event in either arm are shown with zero weight. (**f**) Mesorectal specimen quality graded complete or near-complete, risk difference. Panels **e** and **f** exclude the result at critical risk of bias, in accordance with ROBINS-I guidance. All models are random-effects with restricted maximum-likelihood estimation and a Hartung–Knapp adjustment; the bar beneath each diamond is the prediction interval. A risk ratio below 1.00 or a negative risk difference favours the da Vinci Xi, except in panel **f**, where the event is a success and the axis is oriented accordingly

The evidence is very uncertain about the effect of platform on overall postoperative complications (risk ratio 0.91, 0.55 to 1.52; ad hoc 0.46 to 1.81; five cohorts, 406 patients, 56 events): 13 fewer per 1000 (66 fewer to 75 more) against a Si risk of 145 per 1000 (Fig. 3b). The composite was defined in four ways, only one cohort stating a 30-day window (Supplementary Table 6).

Anastomotic leak, analysed among patients who received an anastomosis, was less frequent with the Xi in four of five cohorts, but the evidence is very uncertain (risk ratio 0.53, 0.24 to 1.14; ad hoc 0.15 to 1.88; 449 patients, 22 events): 32 fewer per 1000 (52 fewer to 9 more) against a Si risk of 68 per 1000 (Fig. 3a). The risk difference excluded zero with the Hartung–Knapp interval (− 3.3 per cent, − 5.7 to − 1.0) but not with the ad hoc correction (− 8.0 to + 1.4); the Peto odds ratio was 0.45 (0.19 to 1.09), and including the cohort reporting anastomotic complications gave 0.83 (0.33 to 2.05). The leak counts for one cohort were confirmed with its corresponding author [23].

Conversion occurred in five of 560 patients (3 of 260 with the Xi, 2 of 300 with the Si); the risk difference was + 0.07 per cent (− 0.70 to + 0.83; ad hoc − 1.84 to + 1.97) (Fig. 3c), and ratio estimates were uninformative (Table 2). Reoperation occurred in 16 of 353 patients, more often with the Xi in two of three cohorts including the largest (risk difference + 2.6 per cent, − 5.3 to + 10.6) (Fig. 3d). Length of stay was 1.1 days shorter with the Xi (− 3.7 to + 1.5; four cohorts, 529 patients; I² 82%; prediction interval − 6.4 to + 4.3) (Fig. 2c), under definitions and discharge policies that differed between health systems.

**Table 2:** Pooled estimates, alternative intervals and sensitivity analyses. Experimental group = da Vinci Xi; control group = da Vinci Si

Outcome	Cohorts (patients)	Primary estimate (95% CI)	95% CI with ad hoc correction	I² (95% CI)	95% prediction interval	Sensitivity analyses
*Operative efficiency*						
Operative time, min	5 (553)	MD − 27.68 (− 80.38 to + 25.03)	−81.38 to + 26.03	95.4% (91.8 to 97.4)	−153.98 to + 98.62	Four cohorts with mean ± SD, conditional on excluding the largest cohort: −50.57 (− 69.42 to − 31.73; ad hoc − 72.21 to − 28.93), I² 18.0%. Largest cohort’s Xi arm entered at 116: −27.53 (− 80.37 to + 25.32). Without the cohort whose endpoint may be console time: −19.45 (− 91.01 to + 52.12); in the four-cohort set − 41.36 (− 70.76 to − 11.96). Adding the mixed colorectal cohort: −26.82 (− 66.92 to + 13.27). Leave-one-out − 50.57 to − 19.45
Docking time, min	3 (199)	Not pooled: −3.10, − 6.00 and − 2.96 by cohort; 3 of 3 favour the Xi (sign test *P* = 0.25)	—	—	—	Shown for completeness only: pooled MD − 4.04 (− 8.32 to + 0.24); standardised mean difference − 1.26 (− 2.72 to + 0.19)
Length of stay, days	4 (529)	MD − 1.07 (− 3.67 to + 1.52)	−3.67 to + 1.52	82.4% (54.7 to 93.2)	−6.39 to + 4.25	Three cohorts with mean ± SD: −0.51 (− 4.14 to + 3.11), I² 74.8%. Leave-one-out − 1.70 to − 0.51
*Perioperative safety*						
Overall complications	5 (406)	RR 0.91 (0.55 to 1.52)	0.46 to 1.81	0% (0 to 62.6)	0.46 to 1.81	RD − 1.1 (− 8.0 to + 5.7). Reported counts only: 0.83 (0.44 to 1.57). GLMM OR 0.90 (0.50 to 1.60). Leave-one-out 0.83 to 1.04
Anastomotic leak	5 (449)	RR 0.53 (0.24 to 1.14)	0.15 to 1.88	0% (0 to 43.1)	0.15 to 1.88	RD − 3.3 (− 5.7 to − 1.0; ad hoc − 8.0 to + 1.4). Peto OR 0.45 (0.19 to 1.09). Including the cohort reporting anastomotic complications: 0.83 (0.33 to 2.05). Smallest defensible denominators: 0.52 (0.25 to 1.12). GLMM OR 0.43 (0.17 to 1.11). Leave-one-out 0.47 to 0.61
Conversion to open surgery	5 (560); 5 events	RD + 0.07 (− 0.70 to + 0.83)	−1.84 to + 1.97	0% (0 to 0)	−1.84 to + 1.97	Xi 3/260, Si 2/300. RR 1.41 (0.19 to 10.29; three informative cohorts). Peto OR 1.53 (0.08 to 29.57). GLMM OR 1.55 (0.25 to 9.60)
Reoperation	3 (353); 16 events	RD + 2.6 (− 5.3 to + 10.6)	−7.1 to + 12.3	0% (0 to 84.6)	−7.1 to + 12.3	Xi 10/174, Si 6/179. RR 1.66 (0.25 to 11.22). Peto OR 1.74 (0.19 to 15.67). GLMM OR 1.76 (0.63 to 4.98)
*Oncological and pathological*						
CRM involvement	4 informative of 6 (395 of 512); 14 events	RR 1.02 (0.23 to 4.49)	0.16 to 6.48	0% (0 to 76.2)	0.16 to 6.48	Retaining the critical-risk result: RR 1.08 (0.38 to 3.10), Peto OR 1.25 (0.30 to 5.24), rectal-only cohorts 1.29 (0.20 to 8.17). GLMM OR 1.20 (0.38 to 3.77). Leave-one-out 0.73 to 1.21
Mesorectal quality, complete or near-complete	4 (338); 11 incomplete	RD − 0.6 (− 6.2 to + 4.9)	−6.9 to + 5.6	0% (0 to 80.6)	−6.9 to + 5.6	Success-scale RR 0.99 (0.94 to 1.05). Retaining the critical-risk result: RD 0.0 (− 4.8 to + 4.8); failure-scale RR 0.81 (0.06 to 11.91). GLMM OR for an incomplete specimen 1.09 (0.25 to 4.73). Leave-one-out − 1.5 to + 1.0
Lymph node yield, n	5 (589)	MD + 2.29 (− 3.08 to + 7.66)	−3.08 to + 7.66	73.2% (33.1 to 89.3)	−8.32 to + 12.90	Four cohorts with mean ± SD: +2.02 (− 6.24 to + 10.29), I² 74.3%. Post hoc, exploratory, without the discordant cohort: +0.16 (− 4.48 to + 4.80). Leave-one-out + 1.18 to + 3.27

MD mean difference, RR risk ratio, OR odds ratio, RD risk difference in percentage points, GLMM binomial-normal generalised linear mixed model, SD standard deviation, CI confidence interval, CRM circumferential resection margin. Primary estimates are random-effects (restricted maximum likelihood) with a Hartung–Knapp interval. The ad hoc correction replaces the Hartung–Knapp standard error with the conventional one whenever the latter is larger; where the two intervals differ in whether they include no effect, the wider is used for interpretation. Prediction intervals use a t distribution with k − 1 degrees of freedom. Risk ratios are pooled from study-level estimates to which 0.5 is added in studies with a zero cell; cohorts with no event in either arm contribute no information to ratio estimates but are retained in risk differences and GLMMs. A negative MD or RD, or a risk ratio below 1.00, favours the da Vinci Xi, except for lymph node yield and mesorectal quality, where a higher value favours the Xi. Changed or added in response to peer review: the primary analyses of operative time, length of stay and lymph node yield, which include the largest cohort with converted medians and were sensitivity analyses in the protocol; the risk-difference summaries of conversion, reoperation and mesorectal quality, for which the protocol specified the risk ratio; the ad hoc intervals; the GLMMs; and the operative-time analyses with the largest cohort’s Xi arm at 116 and without the cohort whose endpoint may be console time. The lymph node analysis without the discordant cohort is post hoc and exploratory. Leave-one-out ranges refer to the primary analysis

### Oncological and pathological outcomes

The evidence is very uncertain about the effect of platform on circumferential margin involvement (risk ratio 1.02, 0.23 to 4.49; ad hoc 0.16 to 6.48; four informative of six cohorts, 14 events) and on mesorectal specimen quality graded complete or near-complete [39, 40] (risk difference − 0.6 per cent, − 6.2 to + 4.9; four cohorts, 338 patients, 11 incomplete specimens) (Fig. 3e, f). Both exclude the one result at critical risk of bias; retaining it gives 1.08 (0.38 to 3.10) and 0.0 per cent (− 4.8 to + 4.8).

Neither result demonstrates equivalence. The margin analysis cannot exclude a fourfold relative increase, and margin involvement is the pathological variable most strongly associated with local recurrence [41]. The success-scale risk ratio for specimen quality specified in the protocol, 0.99 (0.94 to 1.05), looks precise only because more than 95 per cent of specimens were complete and two cohorts with no incomplete specimen carry most of its weight (Supplementary Fig. 32).

Lymph node yield was 2.3 nodes higher with the Xi (− 3.1 to + 7.7; five cohorts, 589 patients; I² 73%; prediction interval − 8.3 to + 12.9) (Fig. 2d). A post hoc analysis omitting the cohort whose arms differed in stage and procedure type (Supplementary Fig. 18) is exploratory and was not used for inference. R0 resection was reported by one cohort and distal margin by two; neither was pooled.

### Outcomes that could not be pooled, and certainty

Four outcomes classified as critical, and one classified as important, could not be pooled. Functional outcomes were reported by one cohort, and only against each group’s own baseline, so no between-group contrast exists [23]. Major complications of Clavien–Dindo grade III or above [42, 43], whose counts for one cohort were supplied by its corresponding author, and diverting stoma formation, excluding one result at critical risk of bias, were each reported by two cohorts, below the three-cohort threshold. Thirty-day mortality was recorded in four cohorts, three with no death, and survival by one cohort [28], which found no association with platform.

Certainty was very low for every outcome (Table 3). Starting at high, as recommended for evidence assessed with ROBINS-I [11], every outcome was rated down two levels for serious risk of bias, three for anastomotic leak, and had at least one further serious limitation: imprecision in all ten, indirectness in five and inconsistency in three. Upgrading was assessed and applied to none (Online Resource 1). Publication bias could not be assessed, the largest analysis containing five informative cohorts against a pre-specified threshold of ten.

**Table 3:** GRADE summary of findings: da Vinci Xi compared with da Vinci Si for rectal cancer surgery, with the judgement for every certainty domain

Outcome	Cohorts (patients)	Risk with Si	Effect with Xi (95% CI)	Risk of bias	Incon- sistency	Indirect- ness	Impre- cision	Publication bias	Cer- tainty	Comment
*Critical outcomes*										
Anastomotic leak	5 (449)	68 per 1000	36 per 1000 (17 to 78); RR 0.53 (0.24 to 1.14)	Very serious^a^	Not serious	Serious^b^	Serious^c^	Not assessable^d^	Very low	Fewer leaks with the Xi in 4 of 5 cohorts; no estimate robustly excludes no difference
Overall complications	5 (406)	145 per 1000	132 per 1000 (79 to 220); RR 0.91 (0.55 to 1.52)	Serious^e^	Not serious^f^	Serious^g^	Serious^c^	Not assessable^d^	Very low	Benefit or harm cannot be excluded
Major complications, Clavien–Dindo ≥ III	2 cohorts (210)	—	Not pooled	—	—	—	—	—	Not graded	8 events; fewer with the Xi in both cohorts; one cohort’s counts supplied by its author
Conversion to open surgery	5 (560)	7 per 1000	1 more per 1000 (7 fewer to 8 more); RD	Serious^e^	Not serious	Not serious	Serious^h^	Not assessable^d^	Very low	5 events (3 Xi, 2 Si); ratio estimates uninformative
Reoperation	3 (353)	34 per 1000	26 more per 1000 (53 fewer to 106 more); RD	Serious^e^	Not serious	Not serious	Very serious^i^	Not assessable^d^	Very low	More frequent with the Xi in 2 of 3 cohorts; not evidence of no difference
CRM involvement	6 (512); 4 informative	28 per 1000	29 per 1000 (7 to 126); RR 1.02 (0.23 to 4.49)	Serious^j^	Not serious	Not serious	Very serious^k^	Not assessable^d^	Very low	Uninformative rather than equivalent
Mesorectal quality, complete or near-complete	4 (338)	970 per 1000	964 per 1000 (908 to 1000^l^); RD − 0.6 points (− 6.2 to + 4.9)	Serious^j^	Not serious	Not serious	Serious^m^	Not assessable^d^	Very low	Almost every specimen complete in both groups
Functional outcomes, 12 months	1 cohort	—	Not estimable	—	—	—	—	—	Not graded	Reported only against each group’s own baseline
30-day mortality	4 cohorts (410)	—	Not pooled	—	—	—	—	—	Not graded	One death, in a Si arm
Disease-free and overall survival	1 cohort	—	Not pooled	—	—	—	—	—	Not graded	No association with platform reported
*Important outcomes*										
Operative time	5 (553)	—	MD − 27.7 min (− 80.4 to + 25.0); conditional on excluding the largest cohort − 50.6 (− 69.4 to − 31.7)	Serious^e^	Serious^n^	Serious^o^	Serious^p^	Not assessable^d^	Very low	Shorter with the Xi only when the largest cohort is excluded
Docking time	3 (199)	—	Not pooled; shorter with the Xi in 3 of 3 cohorts, by 3.0 to 6.0 min	Serious^e^	Not serious^q^	Serious^r^	Serious^s^	Not assessable^d^	Very low	Definitions differ or are not stated
Length of stay	4 (529)	—	MD − 1.1 days (− 3.7 to + 1.5)	Serious^e^	Serious^t^	Serious^u^	Serious^c^	Not assessable^d^	Very low	Discharge policy differs between health systems
Lymph node yield	5 (589)	—	MD + 2.3 nodes (− 3.1 to + 7.7)	Serious^e^	Serious^v^	Not serious	Serious^c^	Not assessable^d^	Very low	Heterogeneity largely from one cohort with non-comparable arms
Diverting stoma	2 cohorts	—	Not pooled	—	—	—	—	—	Not graded	Below the three-cohort threshold once the result at critical risk of bias is set aside

RR risk ratio, RD risk difference, MD mean difference, CI confidence interval, CRM circumferential resection margin. Certainty starts at high because every study was assessed with ROBINS-I, and is rated down two levels for serious and three for very serious risk of bias [11]. For every graded outcome the evidence is very uncertain about the effect of platform. Risks with the Si are the event rates in all Si patients of every cohort in the primary analysis of the outcome, including cohorts with no event in either arm; risks with the Xi are derived from them and the pooled relative effect or risk difference and were not reported by any study. In four cohorts the Xi replaced the Si within the same team; in one, platform coincided with centre; in one, the platforms were used concurrently only in the later period; two did not report the timing. Every estimate therefore reflects the platform together with period, centre and experience

^a^Structural confounding by period, centre or theatre availability, and, in one cohort, leak counts confirmed by its corresponding author

^b^One cohort ascertained leak by routine contrast enema, detecting subclinical leaks

^c^The interval includes both no difference and an appreciable effect

^d^Fewer than ten cohorts; not assessed, which does not mean absent, and not rated down

^e^Structural confounding by period, centre or theatre availability; no cohort could adjust for it

^f^I² of 0% with five cohorts is uninformative; intervals overlap widely

^g^Four definitions of the composite; only one cohort states a 30-day window

^h^Five events in 560 patients: the optimal information size is not met and ratio estimates span a fivefold reduction to a tenfold increase

^i^Sixteen events; the interval spans substantial benefit and harm

^j^As in e; the result at critical risk of bias is excluded

^k^Fourteen events; the interval cannot exclude a fourfold relative increase

^l^Computed upper limit 1020 per 1000, shown as 1000

^m^Eleven incomplete specimens; the optimal information size is not met

^n^I² 95%; the largest cohort points the other way

^o^The endpoint of the cohort carrying most weight in the conditional analysis may be console time, and definitions differ

^p^The interval includes no difference and a large reduction

^q^All three cohorts favour the Xi

^r^A process measure, defined differently or not at all

^s^Three small cohorts, not pooled; three of three in one direction (sign test *P* = 0.25)

^t^I² 82%; the prediction interval spans more than ten days

^u^Discharge policy is set by institutions and health systems rather than by the platform

^v^I² 73%, largely from one cohort whose arms differed in stage and procedure type

## Discussion

Across eight cohorts and 843 patients the evidence is very uncertain about the effect of the Xi on every outcome. Operative time was shorter with the Xi only when the largest cohort was excluded, and docking time was shorter in each of three cohorts that measured it differently.

### The operative-time finding depends on one exclusion

The 50-minute reduction should not be the review’s headline. It rests on a rule that excluded the largest cohort in the literature [26], the only one whose Xi arm was slower; with that cohort included the direction is unchanged, but the interval crosses zero and heterogeneity is 95 per cent. The cohort dominating the conditional estimate does not define its endpoint, and its mean of 124 min is shorter than the console times reported by two other cohorts [21, 27, 28].

The largest cohort’s investigators offer their own explanation [26]: the Xi cases included twenty training cases, and the rest were performed by surgeons early in their robotic experience, at two centres whose robotic programmes began with the Xi. Everywhere else in this literature the later-operated arm is the more experienced one, so confounding by era flatters the Xi; here, where platform coincided with centre and surgeon, the opposite was true, which makes the discordance interpretable rather than anomalous.

The benefit of the Xi may also depend on what the Si arm had already achieved: where the Si still required re-docking or a hybrid laparoscopic phase the Xi removes real steps, whereas a unit already performing single-docking fully robotic surgery on the Si has less to gain. We could not test this, because the two most divergent cohorts describe the same manoeuvre in opposite terms, and the one study comparing both Si approaches within a single institution [24] does not support it.

### Who this comparison informs

The Si is no longer marketed, so the question is not which platform to buy. The comparison informs units still operating Si systems, readers of historical series and registries that mix the two generations, and the evaluation of later platforms, which are being introduced in the same way, one after another in the same units, and will carry the same entanglement of platform, period and experience.

### Pathological outcomes, safety signals and previous syntheses

Circumferential margin involvement and mesorectal specimen quality are least vulnerable to measurement bias, being assessed by a pathologist against standard criteria, and neither showed a difference. Both are imprecise: the margin analysis rests on 14 events, and specimen quality is almost always complete.

Anastomotic leak favoured the Xi in four of five cohorts, and 3.3 fewer leaks per 100 patients would matter clinically if real; but no ratio estimate excludes no difference, the risk difference does so only with the unadjusted Hartung–Knapp interval, and the difference disappears when the definition is widened. This is a hypothesis, not a demonstrated effect, and where the Xi was used later it may reflect changes in anastomotic practice, since no cohort reported stapling technology, perfusion assessment or neoadjuvant regimens by platform. Reoperation was more frequent with the Xi in two of three cohorts including the largest; that estimate is too imprecise to support any conclusion and must not be read as showing no difference.

Our operative-time and docking-time findings point in the same direction as both previous colorectal meta-analyses, but our conclusions differ on two outcomes. The 2025 analysis [5] reported a significant reduction in length of stay of about one day and a lymph node advantage of 2.6 nodes; in rectal-only data both estimates are imprecise and heterogeneous, with prediction intervals spanning clinically important effects in either direction. Four of our eight cohorts appear in neither previous synthesis.

### The functional gap

The Xi redesign targets pelvic exposure and access, whose clinical consequence should be measurable in autonomic function after total mesorectal excision; one cohort in the entire literature has reported those outcomes, and only against each group’s own preoperative baseline, so no between-platform comparison can be extracted [23, 32]. Twelve years after the Xi entered practice, the domain the redesign most plausibly influences remains essentially unstudied.

### Limitations

In four cohorts the Xi replaced the Si within the same team, so the groups were operated in different periods by surgeons further along their learning curve; in one, platform coincided with centre; in one, the platforms were used concurrently only later; and two did not report the timing. Period and centre cannot be adjusted for when they are collinear with the exposure. One cohort shows that the surgeon’s learning curve was completed only at the forty-fourth case and that platform and learning curve were independently associated with operating time [27, 44]. Every estimate here, and in the two prior syntheses, therefore reflects the platform together with period, centre and experience, and co-interventions that changed over time — stapling technology, perfusion assessment, neoadjuvant regimens, enhanced recovery and transanal approaches — were not reported by platform and may mediate any difference.

The evidence is thin: eight retrospective cohorts, at most five informative per analysis and no more than 22 events for any dichotomous outcome except overall complications. One cohort is known only from a conference abstract, baseline characteristics are incompletely reported, and outcome definitions varied materially; one cohort ascertained anastomotic leak by routine contrast enema, detecting subclinical leaks the others never sought [23]. The two Istanbul cohorts both include Si-era cases from one hospital, operated by different surgeons, so some overlap of Si-arm patients cannot be excluded. One otherwise eligible cohort was excluded after data extraction; it favoured the Xi on every perioperative outcome, so the exclusion is conservative. Several analyses were changed after peer review, each reported with its pre-specified counterpart. Trial registers were not searched, citation searching was incomplete, and no outcome approached the ten cohorts needed to assess small-study effects, so publication bias is undetermined.

### Implications and future research

These data neither demonstrate nor exclude an oncological difference between the platforms, and any efficiency gain from the Xi is unconfirmed and, where observed, measured against a comparator operating in an earlier era or at another centre. Future comparisons should use platforms available concurrently in the same units; where randomisation is impractical, prospective registries should record platform with surgeon case number, docking approach, co-interventions and standard outcome definitions, including a common definition of docking time. Functional outcomes measured with validated instruments, and long-term oncological outcomes, are the priority.

## Conclusion

In rectal cancer surgery the evidence is very uncertain about the effect of the da Vinci Xi compared with the Si on operative and docking time, anastomotic leak, complications, conversion, reoperation, margin involvement, specimen quality, length of stay and lymph node yield; operative time was shorter with the Xi only when the largest cohort was excluded. Certainty is very low for every outcome, and because the platforms were compared across periods, centres and levels of experience, none of these estimates supports a causal claim about the platform. Functional outcomes have not been compared between platforms and should be the priority for future work.

## Supplementary Information

Below is the link to the electronic supplementary material.

Supplementary Material 1: The online version contains supplementary material. Online Resource 1 (PDF) is the Supplementary Material: search execution notes, outcome definitions and denominators, the eligibility revision applied after data extraction, every deviation from the protocol and every change made in response to peer review (Section S7), and Supplementary Tables 1 to 7 — the full search strategies, the excluded reports with reasons, the complete outcome-level extraction, the ROBINS-I V2 assessment of all 23 study results, the GRADE evidence profile, the outcome definitions of every cohort, and the timing, centres and co-interventions of each platform arm. Online Resource 2 (PDF) contains Supplementary Figures 1 to 33: the risk-of-bias summary, the exploratory operative-time display, seven leave-one-out analyses and twenty-four sensitivity analyses. Online Resource 3 (PDF) is the PRISMA 2020 checklist. Online Resource 4 (ZIP) contains the analysis datasets as comma-separated files, every result table, and the complete R script that reproduces every estimate and every figure in this review from them.

Supplementary Material 2

Supplementary Material 3

Supplementary Material 4

## Data Availability

All data supporting the findings of this study are available within the paper and its Online Resources. Online Resource 1, Supplementary Table 3, lists every value extracted from every included publication, with the outcome definition and denominator for each and with values obtained by arithmetic rather than printed in the source identified as derived. Online Resource 4 contains those data as comma-separated files. This review generated no new patient-level data and deposited no dataset in a repository; the secondary data analysed are the published reports of the included studies, which are cited in the reference list. One quantity used here is not printed in its source publication: the counts of major complications of Clavien–Dindo grade III or above for one cohort, which were supplied by that study’s corresponding author in personal correspondence. They are reproduced with that provenance identified in Online Resource 1, and the correspondence is held by the authors and can be made available on reasonable request with the permission of the author who supplied it.

## Code availability

The complete R code that produces every estimate, every table entry and every figure in this report from the datasets is provided as Online Resource 4. It is a single self-contained script; the datasets are embedded in it, so it runs without any other file, and it checks every primary estimate against an independent computation.

## Declarations

### Use of large language models

A large language model was used only to translate draft material into English and to adapt the manuscript to the journal’s formatting requirements. It contributed no scientific content, and the authors are accountable for the whole of the final text.
