# Supplementary material for: Comparative perioperative and oncological outcomes of the da Vinci Xi versus Si robotic surgical systems in rectal cancer surgery: a systematic review and meta-analysis
Source: J Robot Surg. 2026 Oct 20;20(1):1115. doi: 10.1007/s11701-026-04059-0 (PMC13683476; doi:10.1007/s11701-026-04059-0)

Online Resource 2 — Supplementary Figures 1 to 33 (revised)

Comparative Perioperative and Oncological Outcomes of the da Vinci Xi
Versus Si Robotic Surgical Systems in Rectal Cancer Surgery:
A Systematic Review and Meta-analysis

Journal of Robotic Surgery

Ivens Murad Garcia Magalhães; Fillipe Augusto de Oliveira Paixão;
Gustavo Tamura; Pedro Averbach

Corresponding author: Ivens Murad Garcia Magalhães,
Faculdade de Medicina, Universidade Federal de Juiz de Fora,
Avenida Presidente Itamar Franco, 2344, Juiz de Fora, Minas Gerais, Brazil.
E-mail: garciaivens@gmail.com

All figures in this file were produced in R with the meta and metafor packages
by the analysis script supplied as Online Resource 4. No figure uses colour.

Throughout, the da Vinci Xi is the experimental group and the da Vinci Si the control group.
An asterisk after a study label marks values converted from medians and interquartile ranges.
Pooled estimates are random-effects (restricted maximum likelihood) with a Hartung–Knapp interval;
the bar beneath each pooled estimate is the 95% prediction interval.

Supplementary Fig. 1 ROBINS-I V2 risk-of-bias summary for the 23 assessed study results. Each row is one study result, not one study. Symbols: + low, − moderate, × serious, ! critical, ? not assessed.

	D1 Confounding	D2 Classification of interventions	D3 Selection of participants	D4 Missing data	D5 Measurement of outcome	D6 Selection of reported result	Overall
Ozben (2016) | G1 Operative efficiency	×	+	−	−	−	−	×
Ozben (2016) | G2 Perioperative safety	×	+	−	−	−	−	×
Ozben (2016) | G3 Pathological/oncological	×	+	×	−	+	−	×
Ozben (2016) | G4 Diverting stoma	!	+	−	−	+	−	!
Cha (2018) | G3 Pathological/oncological	!	?	?	?	?	?	!
Morelli (2018) | G1 Operative efficiency	×	+	+	−	−	−	×
Morelli (2018) | G2 Perioperative safety	×	+	+	+	−	×	×
Morelli (2018) | G3 Pathological/oncological	×	+	+	+	+	−	×
Morelli (2018) | G4 Diverting stoma	−	+	+	−	+	−	×
Tung (2018) | G2 Perioperative safety	×	+	−	−	−	×	×
Tung (2018) | G3 Pathological/oncological	×	+	−	−	+	×	×
Huang (2019) | G1 Operative efficiency (sens.)	×	+	×	−	−	−	×
Huang (2019) | G3 Pathological/oncological	×	+	−	−	+	−	×
Panteleimonitis (2020) | G1 Operative efficiency	×	+	−	−	−	−	×
Panteleimonitis (2020) | G2 Perioperative safety	×	+	−	−	−	−	×
Panteleimonitis (2020) | G3 Pathological/oncological	×	+	−	−	+	−	×
Aliyev (2022) | G1 Operative efficiency	×	+	−	−	−	×	×
Aliyev (2022) | G2 Perioperative safety	×	+	−	−	−	×	×
Aliyev (2022) | G3 Pathological/oncological	×	+	−	−	+	×	×
Aliyev (2022) | G4 Diverting stoma	×	+	−	−	+	×	×
Wang (2026) | G1 Operative efficiency	×	+	−	−	−	−	×
Wang (2026) | G2 Perioperative safety	×	+	−	−	−	−	×
Wang (2026) | G3 Pathological/oncological	×	+	−	−	+	−	×

+ Low   − Moderate   × Serious   ! Critical   ? Not assessed

Supplementary Fig. 2 Total operative time by cohort, annotated with each study's own description of its da Vinci Si operative approach. Exploratory and non-inferential: no pooling, no subgroup test and no p values.

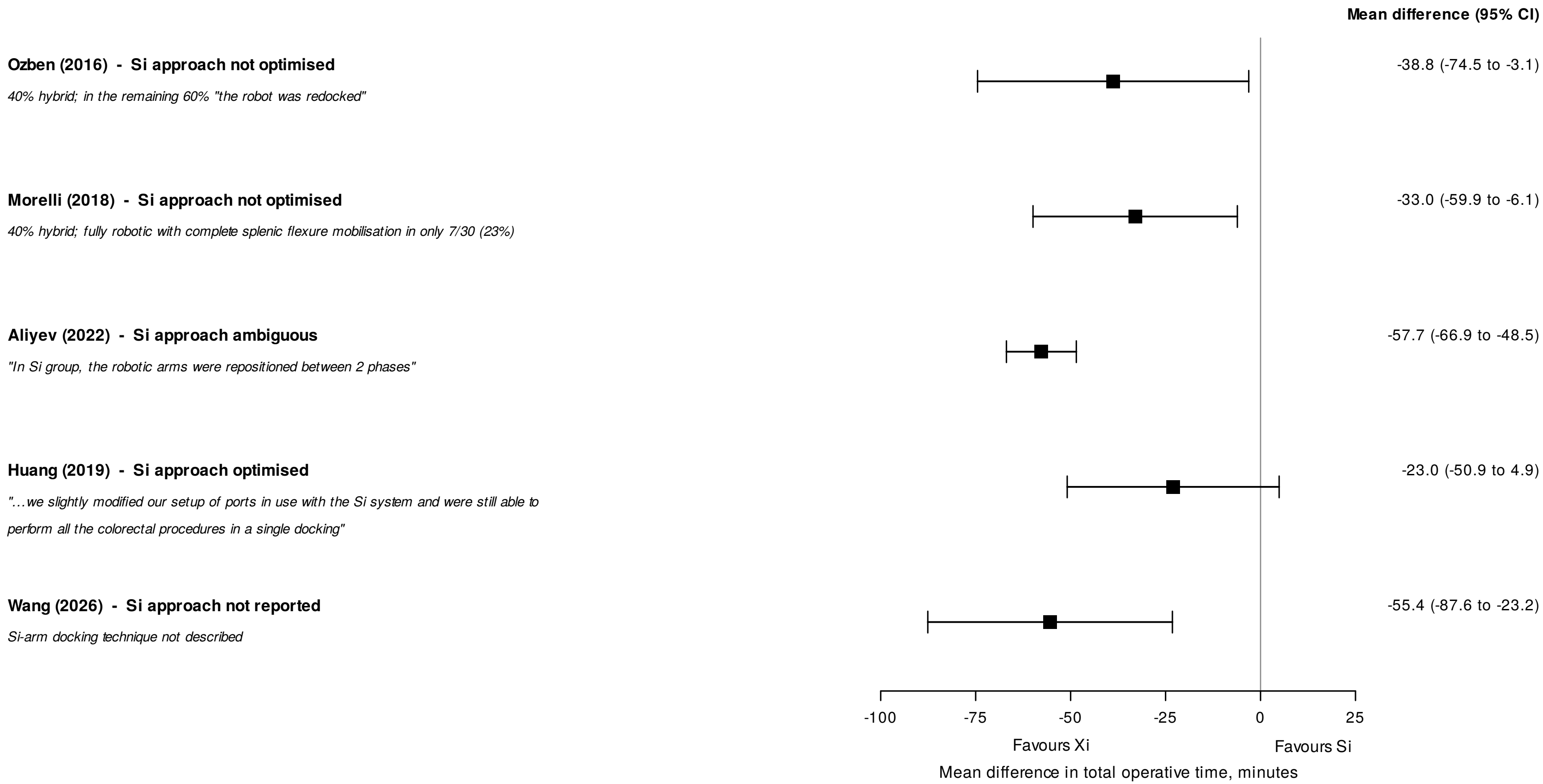

Supplementary Fig. 3 Leave-one-out analysis, total operative time, primary analysis (five cohorts).

Total operative time, minutes

**Analysis**	**MD (95% CI)**	**I²**
Omitting Ozben (2016)	−25.23 (−101.59 to 51.13)	97%
Omitting Morelli (2018)	−26.49 (−103.94 to 50.96)	97%
Omitting Aliyev (2022)	−19.45 (−91.01 to 52.12)	93%
Omitting Wang (2026)	−21.17 (−93.52 to 51.17)	96%
Omitting Panteleimonitis (2020)*	−50.57 (−69.42 to −31.73)	18%
**All cohorts**	**−27.68 (−80.38 to 25.03)**	**95%**

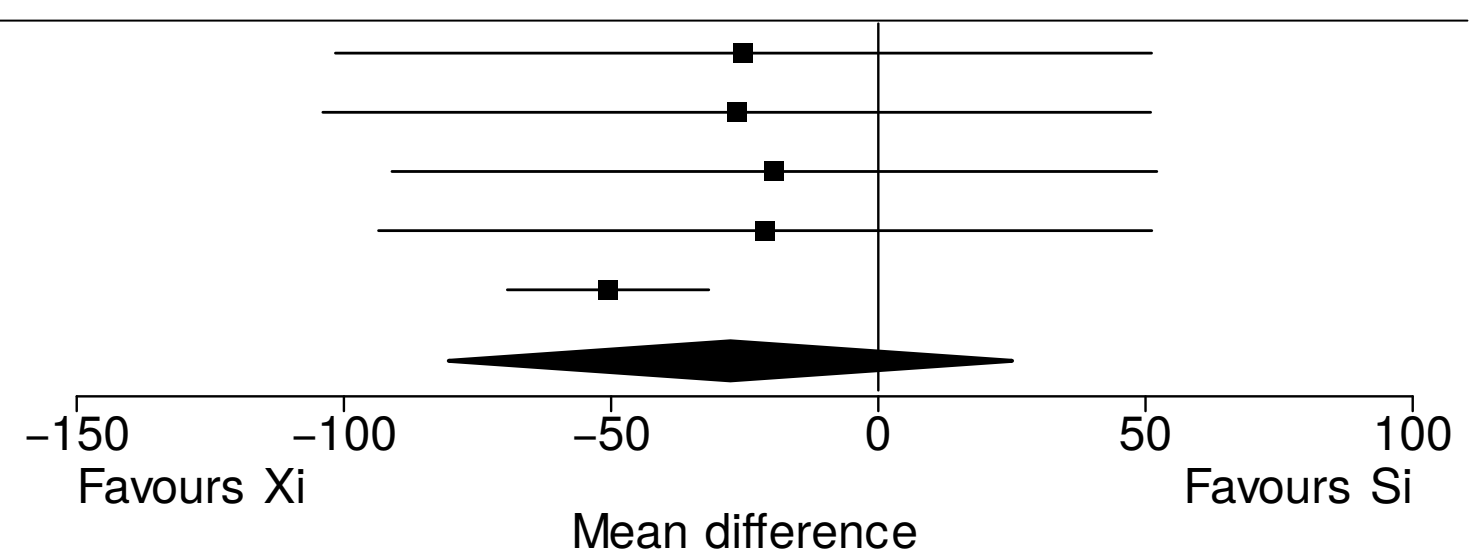

Supplementary Fig. 4 Leave-one-out analysis, length of hospital stay, primary analysis (four cohorts).

Length of hospital stay, days

**Analysis**	**MD (95% CI)**	**I^2**
Omitting Ozben (2016)	−1.70 (−4.40 to 1.00)	74%
Omitting Aliyev (2022)	−1.22 (−6.16 to 3.72)	86%
Omitting Wang (2026)	−0.79 (−5.47 to 3.90)	87%
Omitting Panteleimonitis (2020)*	−0.51 (−4.14 to 3.11)	75%
**All cohorts**	**−1.07 (−3.67 to 1.52)**	**82%**

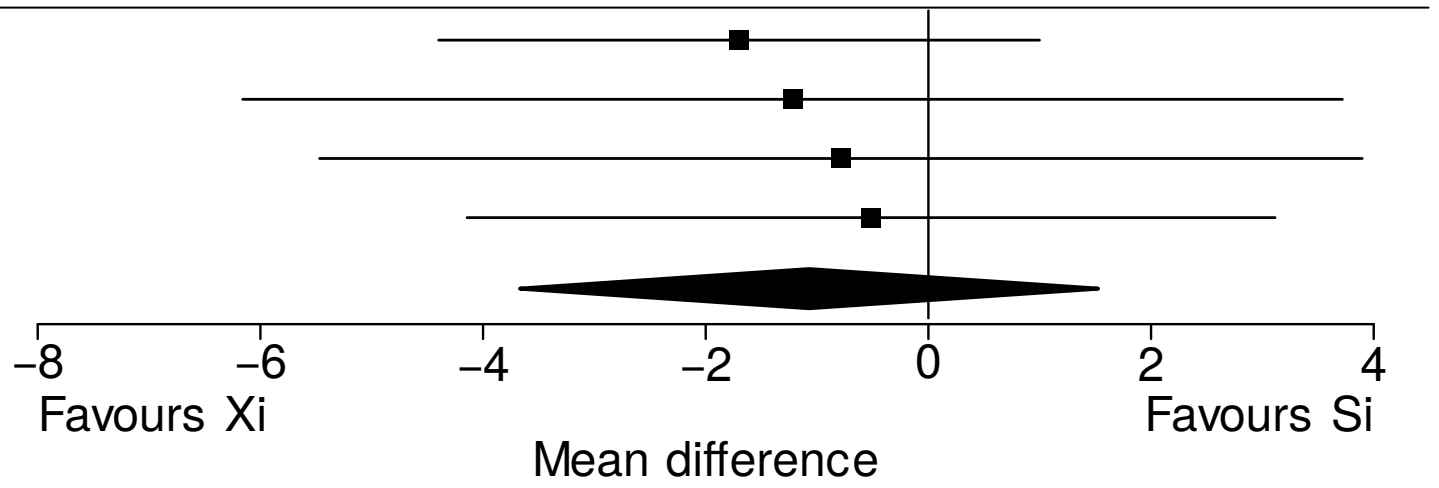

Supplementary Fig. 5 Leave-one-out analysis, lymph node yield, primary analysis (five cohorts).

Lymph node yield, nodes

**Analysis**	**MD (95% CI)**	**I^2**
Omitting Ozben (2016)	1.18 (−2.78 to 5.13)	63%
Omitting Morelli (2018)	3.27 (−2.84 to 9.39)	67%
Omitting Aliyev (2022)	2.91 (−5.08 to 10.89)	78%
Omitting Wang (2026)	2.65 (−5.62 to 10.91)	79%
Omitting Panteleimonitis (2020)*	2.02 (−6.24 to 10.29)	74%
**All cohorts**	**2.29 (−3.08 to 7.66)**	**73%**

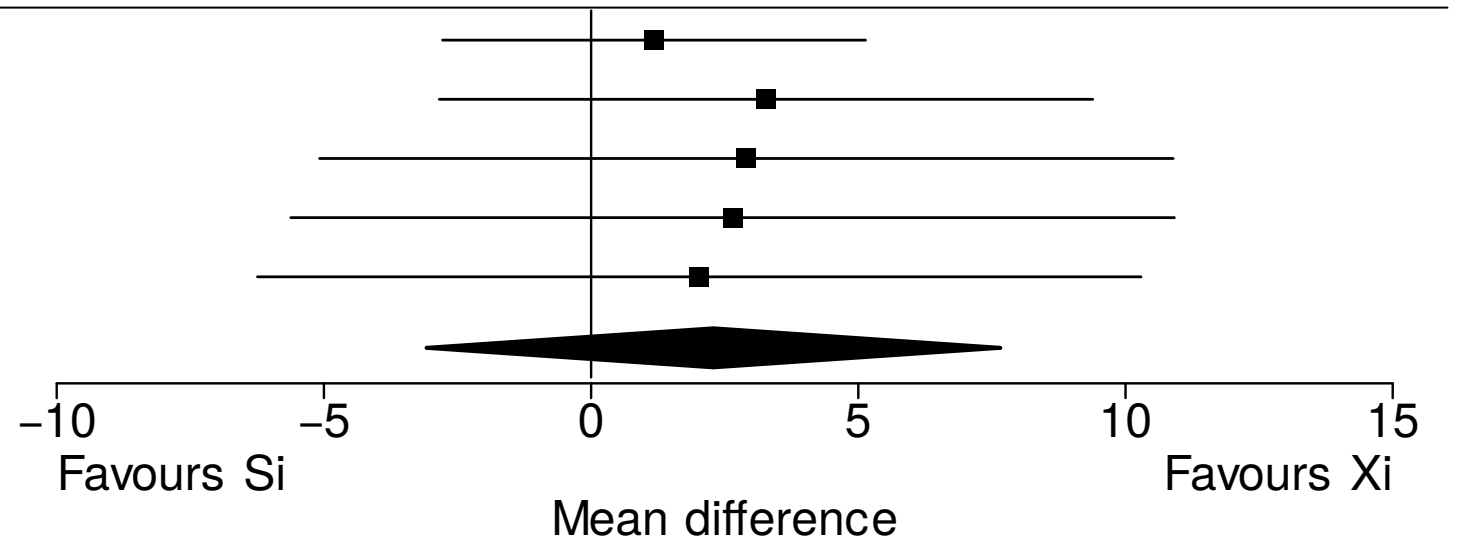

Supplementary Fig. 6 Leave-one-out analysis, overall postoperative complications.

Overall postoperative complications

**Analysis**	**RR (95% CI)**	**I^2**
Omitting Ozben (2016)	0.88 (0.44 to 1.76)	0%
Omitting Morelli (2018)	0.98 (0.46 to 2.08)	0%
Omitting Tung (2018)	0.83 (0.44 to 1.57)	0%
Omitting Aliyev (2022)	0.85 (0.40 to 1.80)	0%
Omitting Wang (2026)	1.03 (0.67 to 1.59)	0%
**All cohorts**	**0.91 (0.55 to 1.52)**	**0%**

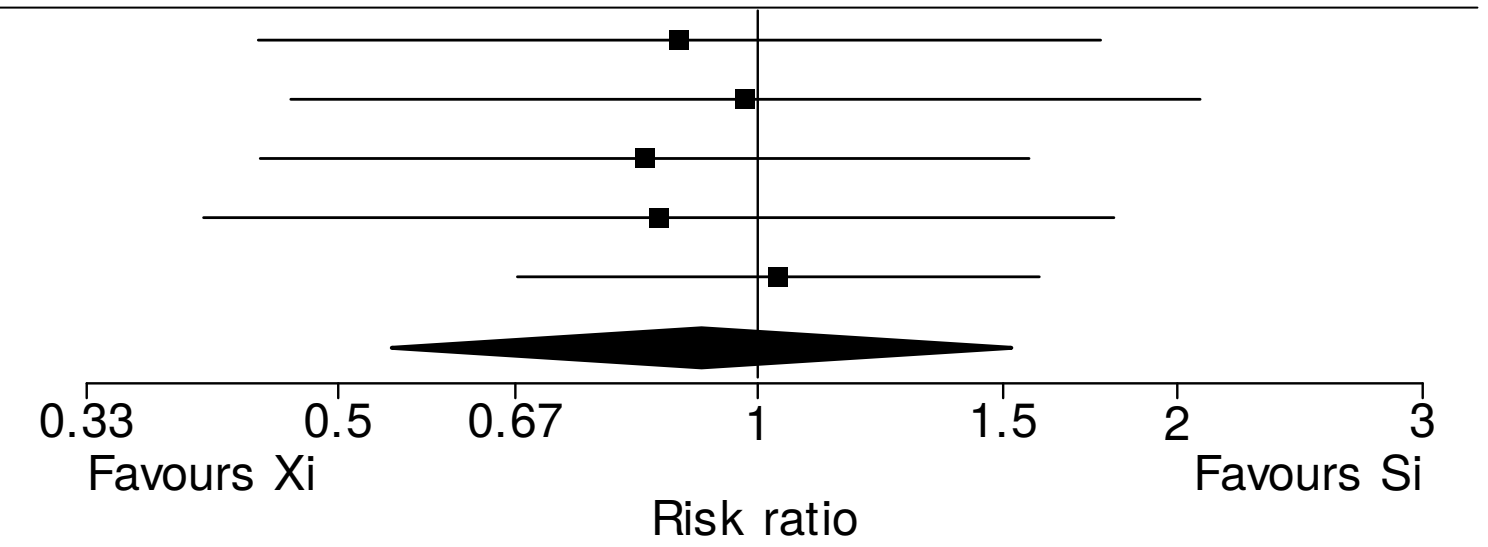

Supplementary Fig. 7 Leave-one-out analysis, anastomotic leak.

Anastomotic leak

Analysis	RR (95% CI)	I^2
Omitting Ozben (2016)	0.55 (0.19 to 1.63)	0%
Omitting Morelli (2018)	0.48 (0.15 to 1.53)	0%
Omitting Tung (2018)	0.51 (0.15 to 1.79)	0%
Omitting Panteleimonitis (2020)	0.61 (0.35 to 1.07)	0%
Omitting Wang (2026)	0.47 (0.18 to 1.18)	0%
**All cohorts**	**0.53 (0.24 to 1.14)**	**0%**

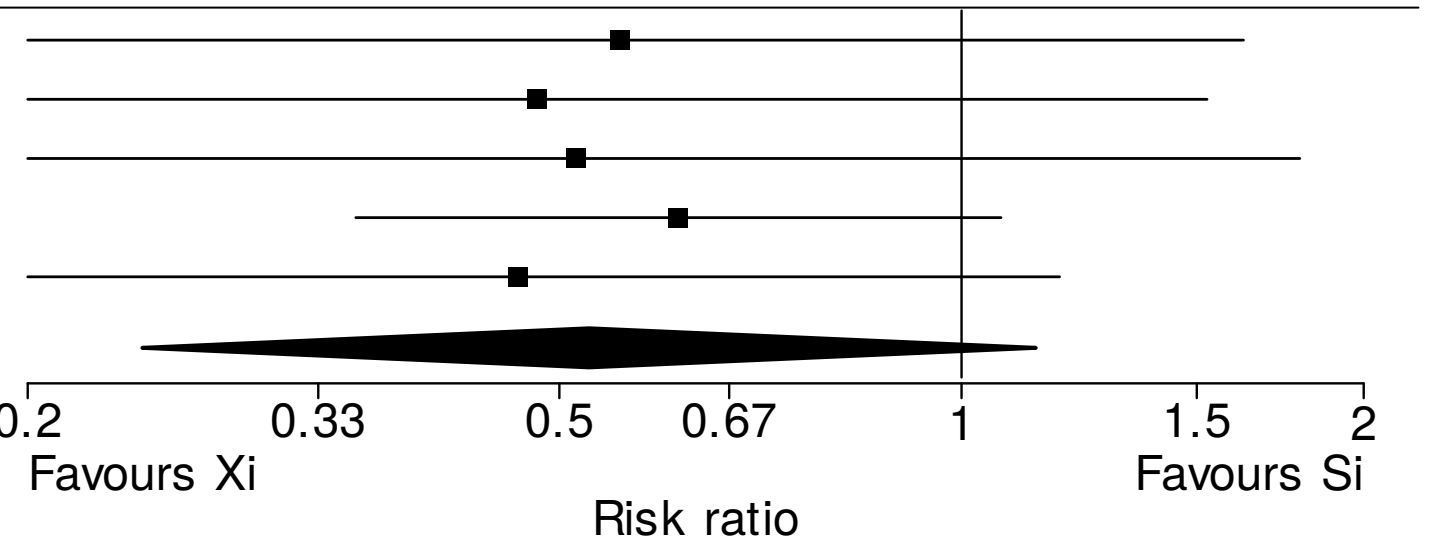

Supplementary Fig. 8 Leave-one-out analysis, circumferential resection margin involvement.

Circumferential resection margin involvement

**Analysis**	**RR (95% CI)**	**I^2**
Omitting Ozben (2016)	0.73 (0.34 to 1.61)	0%
Omitting Huang (2019)	1.21 (0.03 to 43.10)	0%
Omitting Aliyev (2022)	1.14 (0.10 to 13.19)	0%
Omitting Wang (2026)	1.15 (0.09 to 14.68)	0%
**All cohorts**	**1.02 (0.23 to 4.49)**	**0%**

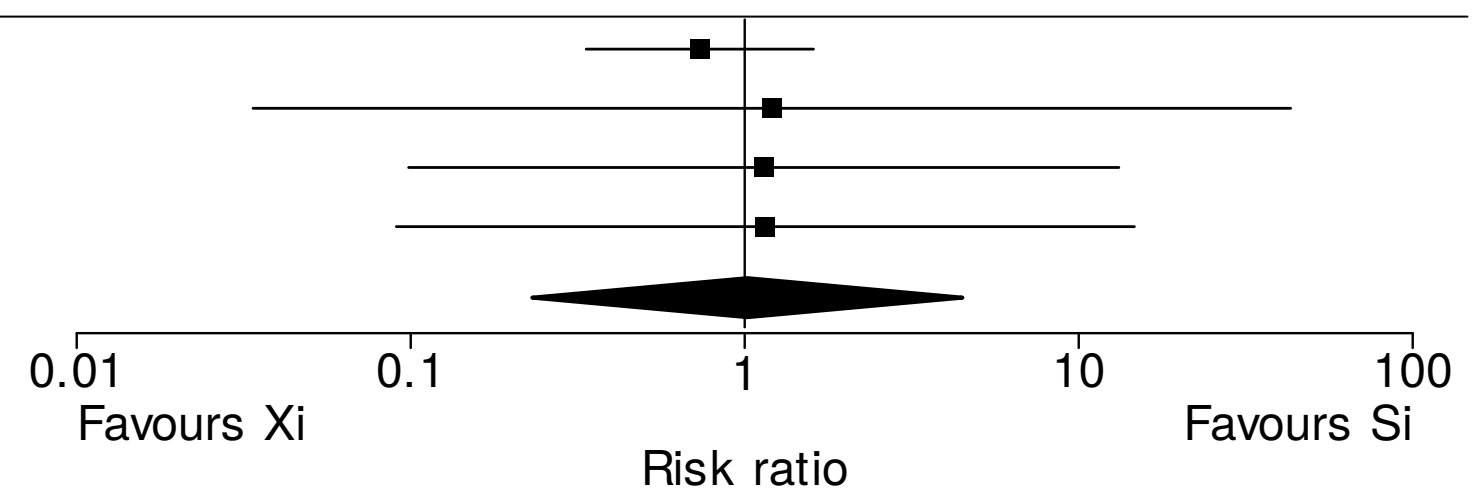

Supplementary Fig. 9 Leave-one-out analysis, mesorectal specimen quality (risk difference, percentage points).

Mesorectal specimen complete or near-complete

**Analysis**	**RD, % (95% CI)**	**I²**
Omitting Ozben (2016)	−0.8 (−11.0 to 9.4)	15%
Omitting Morelli (2018)	−0.9 (−12.6 to 10.9)	13%
Omitting Aliyev (2022)	1.0 (−5.8 to 7.8)	0%
Omitting Wang (2026)	−1.5 (−7.6 to 4.6)	0%
**All cohorts**	**−0.6 (−6.2 to 4.9)**	**0%**

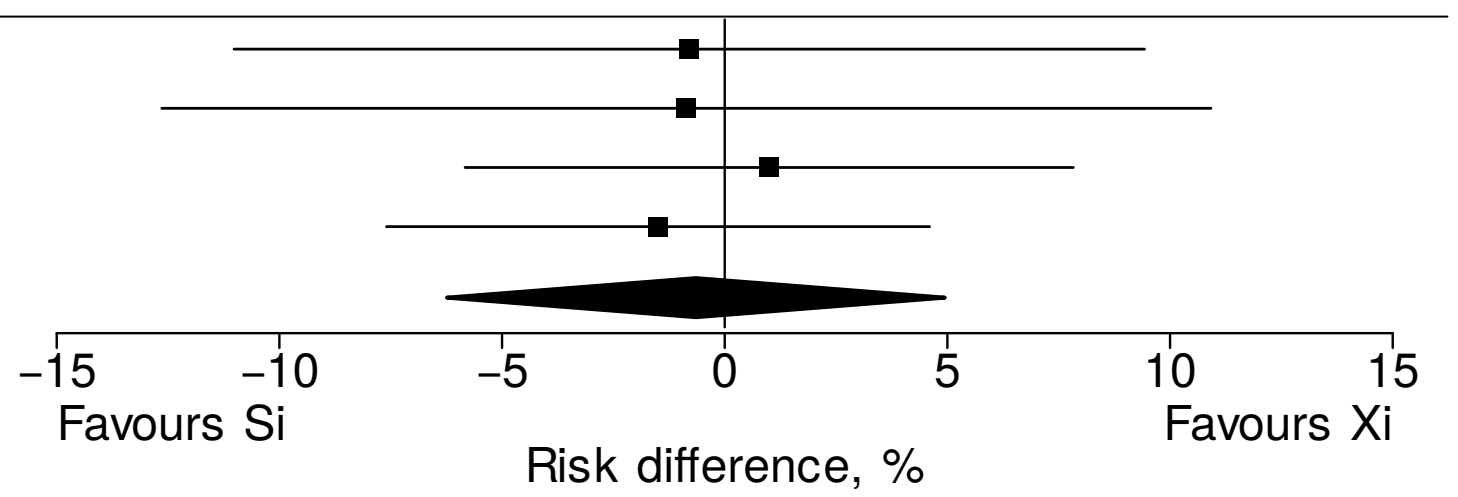

Supplementary Fig. 10 Total operative time, with the largest cohort's Xi arm entered at the printed arm size (116) rather than the 80 patients whose operative time was recorded.

Total operative time, minutes; largest cohort's Xi arm at 116

**Study**	**da Vinci Xi** Mean (SD), n	**da Vinci Si** Mean (SD), n	**Weight**	**MD (95% CI)**
Ozben (2016)	321.6 (69.4), 28	360.4 (63.2), 25	18.4%	−38.80 (−74.50 to −3.10)
Morelli (2018)	285.0 (49.0), 30	318.0 (57.0), 30	19.8%	−33.00 (−59.90 to −6.10)
Aliyev (2022)	123.6 (25.7), 62	181.3 (31.8), 88	21.7%	−57.70 (−66.92 to −48.48)
Wang (2026)	226.7 (57.2), 55	282.1 (80.8), 31	19.0%	−55.42 (−87.62 to −23.22)
Panteleimonitis (2020)*	289.4 (67.6), 116	245.5 (72.0), 124	21.0%	43.98 (26.32 to 61.64)
**Pooled estimate**	**291**	**298**	**100%**	**−27.53 (−80.37 to 25.32)**
Prediction interval				(−154.66 to 99.61)

Heterogeneity: $I^2 = 96\%$ (93 to 98%), $\tau^2 = 1720$, $p < 0.001$

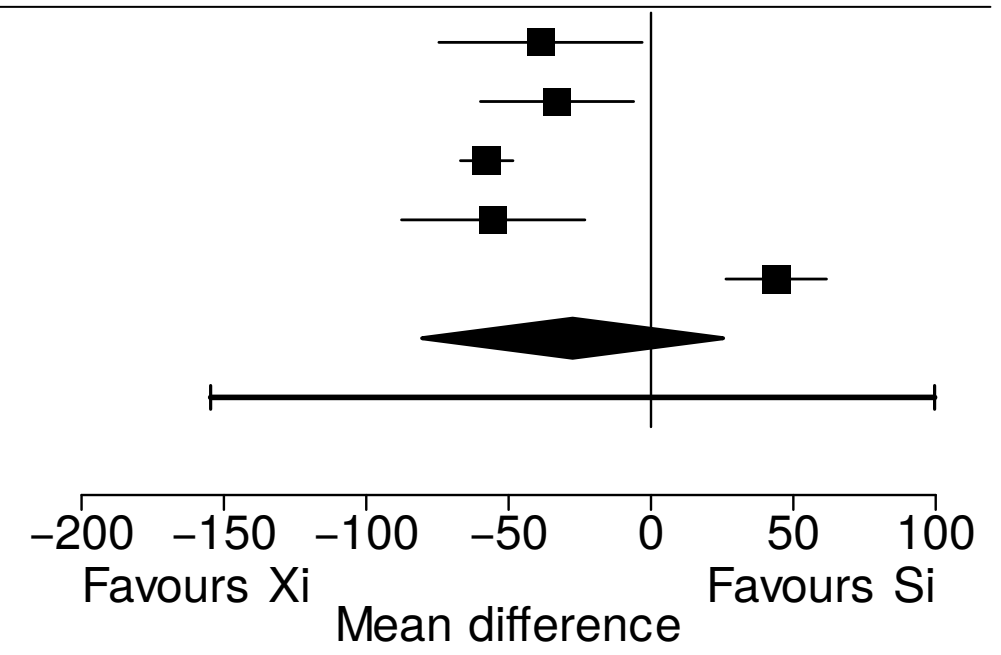

Supplementary Fig. 11 Total operative time, five-cohort set without the cohort whose timing endpoint may be console time (Aliyev 2022).

Total operative time, minutes; five-cohort set without Aliyev (2022)

**Study**	**da Vinci Xi** Mean (SD), n	**da Vinci Si** Mean (SD), n	**Weight**	**MD (95% CI)**
Ozben (2016)	321.6 (69.4), 28	360.4 (63.2), 25	23.7%	−38.80 (−74.50 to −3.10)
Morelli (2018)	285.0 (49.0), 30	318.0 (57.0), 30	25.4%	−33.00 (−59.90 to −6.10)
Wang (2026)	226.7 (57.2), 55	282.1 (80.8), 31	24.4%	−55.42 (−87.62 to −23.22)
Panteleimonitis (2020)*	289.4 (67.9), 80	245.5 (72.0), 124	26.5%	43.96 (24.40 to 63.51)
**Pooled estimate**	**193**	**210**	**100%**	**−19.45 (−91.01 to 52.12)**
Prediction interval				(−175.33 to 136.44)

Heterogeneity: $I^2 = 93\%$ (84 to 97%), $\tau^2 = 1880$, $p < 0.001$

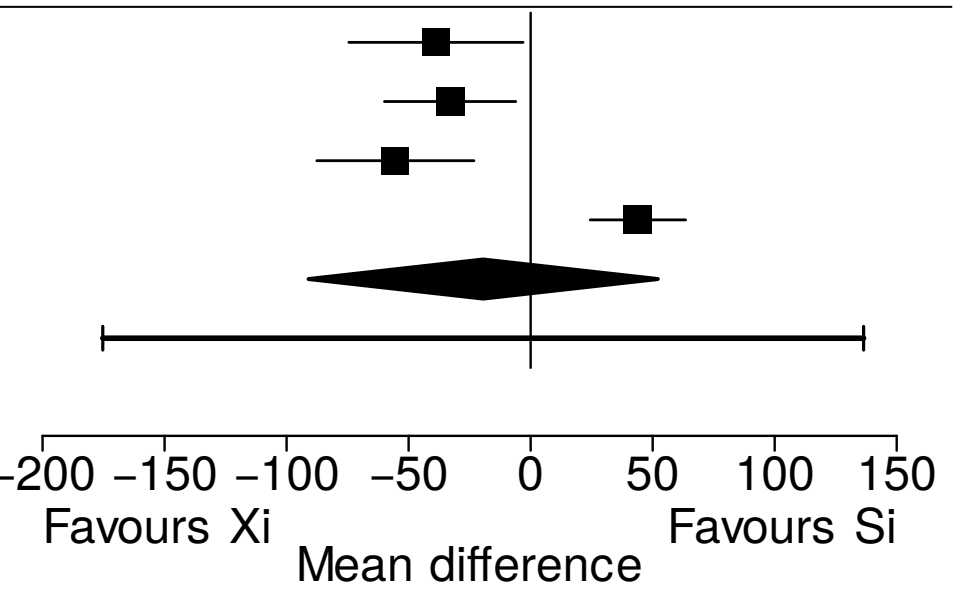

Supplementary Fig. 12 Total operative time, four mean ± SD cohorts without the same cohort.

Total operative time, minutes; four-cohort set without Aliyev (2022)

**Study**	**da Vinci Xi** Mean (SD), n	**da Vinci Si** Mean (SD), n	**Weight**	**MD (95% CI)**
Ozben (2016)	321.6 (69.4), 28	360.4 (63.2), 25	25.1%	−38.80 (−74.50 to −3.10)
Morelli (2018)	285.0 (49.0), 30	318.0 (57.0), 30	44.1%	−33.00 (−59.90 to −6.10)
Wang (2026)	226.7 (57.2), 55	282.1 (80.8), 31	30.8%	−55.42 (−87.62 to −23.22)
**Pooled estimate**	**113**	**86**	**100%**	**−41.36 (−70.76 to −11.96)**
Prediction interval				(−80.59 to −2.13)

Heterogeneity: $I^2 = 0\%$ (0 to 81%), $\tau^2 = 0$, $p = 0.570$

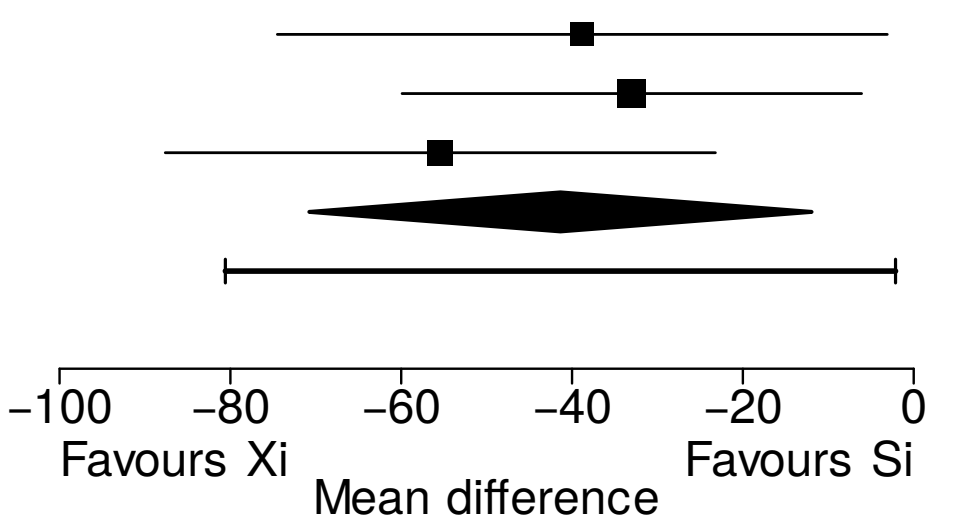

Supplementary Fig. 13 Total operative time, adding the mixed colorectal cohort on its rectal procedures.

Total operative time, minutes; adding the mixed colorectal cohort

Study	da Vinci Xi Mean (SD), n	da Vinci Si Mean (SD), n	Weight	MD (95% CI)
Ozben (2016)	321.6 (69.4), 28	360.4 (63.2), 25	15.2%	−38.80 (−74.50 to −3.10)
Morelli (2018)	285.0 (49.0), 30	318.0 (57.0), 30	16.6%	−33.00 (−59.90 to −6.10)
Aliyev (2022)	123.6 (25.7), 62	181.3 (31.8), 88	18.6%	−57.70 (−66.92 to −48.48)
Wang (2026)	226.7 (57.2), 55	282.1 (80.8), 31	15.7%	−55.42 (−87.62 to −23.22)
Panteleimonitis (2020)*	289.4 (67.9), 80	245.5 (72.0), 124	17.6%	43.96 (24.40 to 63.51)
Huang (2019)	241.8 (60.7), 28	264.8 (72.1), 73	16.4%	−23.00 (−50.91 to 4.91)
**Pooled estimate**	**283**	**371**	**100%**	**−26.82 (−66.92 to 13.27)**
Prediction interval				(−129.93 to 76.28)

Heterogeneity: $I^2 = 94\%$ (90 to 97%), $\tau^2 = 1350$, $p < 0.001$

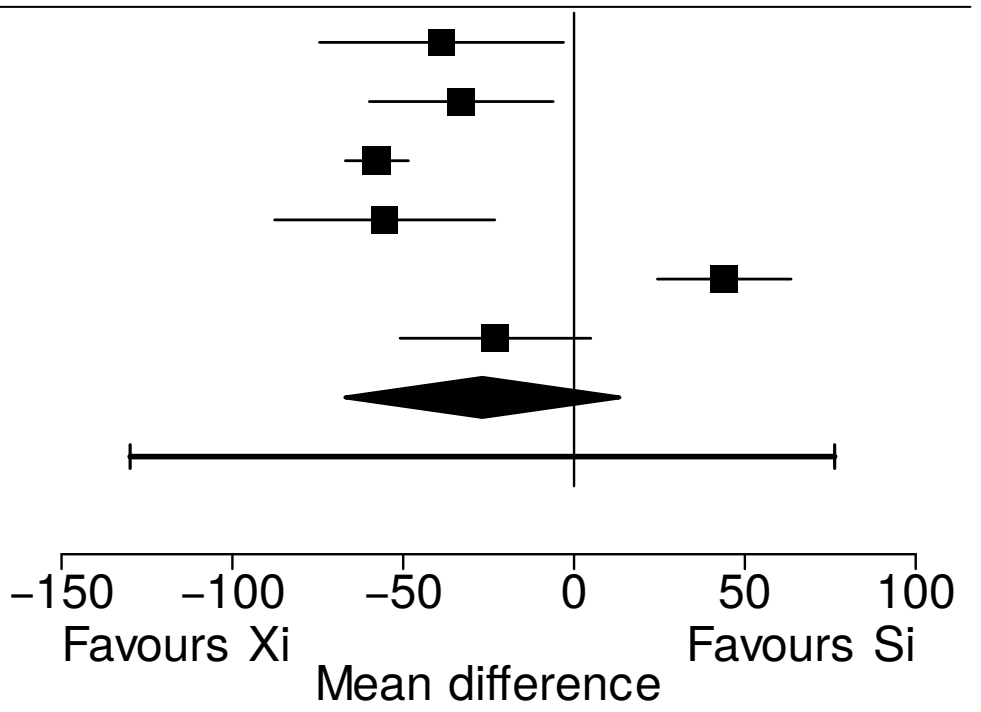

Supplementary Fig. 14 Docking time, pooled mean difference. Shown for completeness only: definitions differ or are not stated, so this estimate is not used for inference.

Docking time, minutes; pooled mean difference (not used for inference)

**Study**	**da Vinci Xi** Mean (SD), n	**da Vinci Si** Mean (SD), n	**Weight**	**MD (95% CI)**
Ozben (2016)	5.3 (1.3), 28	8.4 (3.6), 25	34.5%	−3.10 (−4.59 to −1.61)
Morelli (2018)	17.5 (3.4), 30	23.5 (2.7), 30	33.9%	−6.00 (−7.55 to −4.45)
Wang (2026)	8.8 (3.3), 55	11.7 (4.5), 31	31.6%	−2.96 (−4.76 to −1.16)
**Pooled estimate**	**113**	**86**	**100%**	**−4.04 (−8.32 to 0.24)**
Prediction interval				(−11.86 to 3.78)

Heterogeneity: $I^2 = 78\%$ (28 to 93%), $\tau^2 = 2.31$, $p = 0.011$

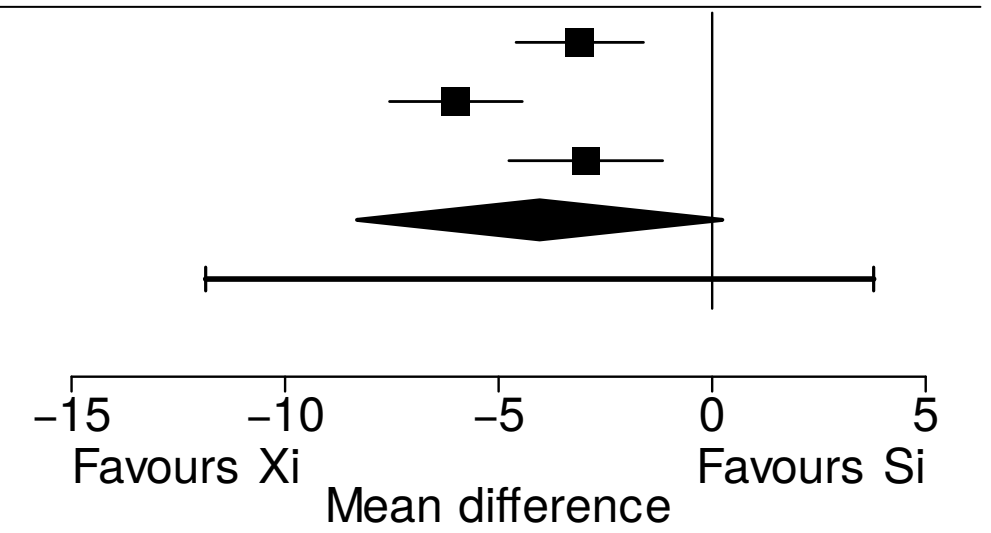

Supplementary Fig. 15 Docking time, standardised mean difference. Not used for inference, for the same reason.

Docking time; standardised mean difference (not used for inference)

**Study**	**da Vinci Xi** Mean (SD), n	**da Vinci Si** Mean (SD), n	**Weight**	**SMD (95% CI)**
Ozben (2016)	5.3 (1.3), 28	8.4 (3.6), 25	32.4%	−1.15 (−1.74 to −0.57)
Morelli (2018)	17.5 (3.4), 30	23.5 (2.7), 30	31.5%	−1.93 (−2.55 to −1.31)
Wang (2026)	8.8 (3.3), 55	11.7 (4.5), 31	36.1%	−0.78 (−1.23 to −0.32)
**Pooled estimate**	**113**	**86**	**100%**	**−1.26 (−2.72 to 0.19)**
Prediction interval				(−3.90 to 1.38)

Heterogeneity: $I^2 = 77\%$ (24 to 93%), $\tau^2 = 0.262$, $p = 0.013$

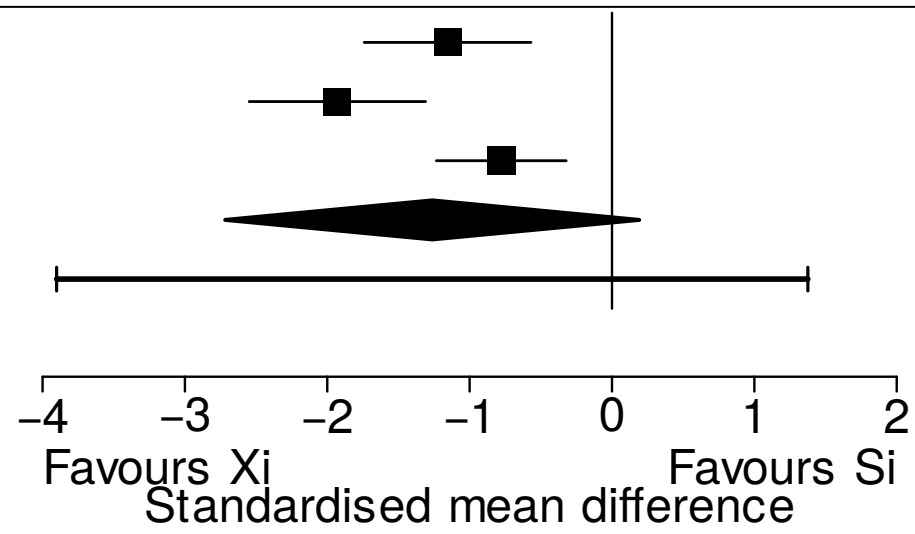

Supplementary Fig. 16 Length of hospital stay, three cohorts reporting a mean with a standard deviation.

Length of hospital stay, days; three cohorts reporting mean and SD

**Study**	**da Vinci Xi** Mean (SD), n	**da Vinci Si** Mean (SD), n	**Weight**	**MD (95% CI)**
Ozben (2016)	6.2 (2.5), 28	5.1 (3.3), 25	30.2%	1.10 (−0.49 to 2.69)
Aliyev (2022)	5.6 (2.8), 62	6.2 (3.4), 88	36.8%	−0.60 (−1.60 to 0.40)
Wang (2026)	7.8 (1.7), 55	9.7 (3.6), 31	32.9%	−1.90 (−3.25 to −0.55)
**Pooled estimate**	**145**	**144**	**100%**	**−0.51 (−4.14 to 3.11)**
Prediction interval				(−6.96 to 5.93)

Heterogeneity: $I^2 = 75\%$ (16 to 92%), $\tau^2 = 1.57$, $p = 0.019$

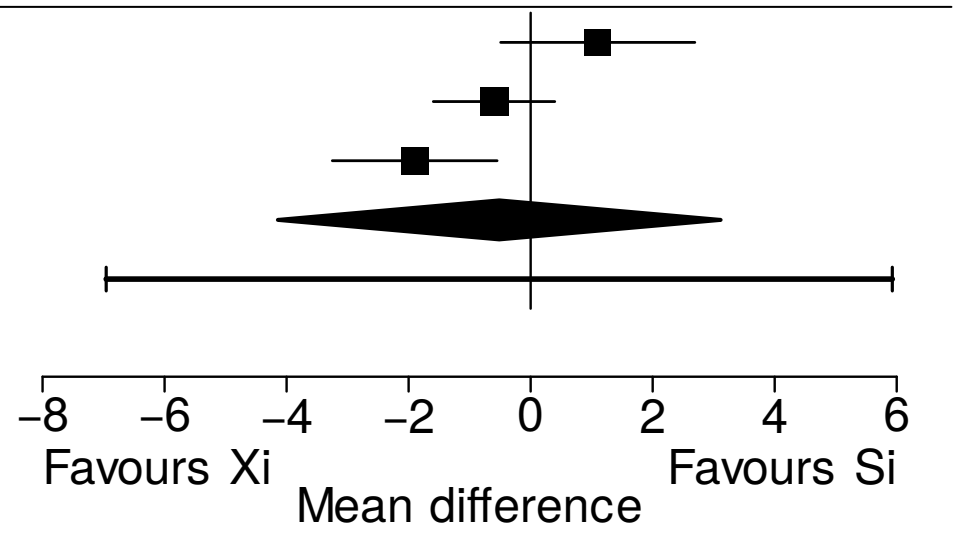

Supplementary Fig. 17 Lymph node yield, four cohorts reporting a mean with a standard deviation.

Lymph node yield, nodes; four cohorts reporting mean and SD

Study	da Vinci Xi<br>Mean (SD), n	da Vinci Si<br>Mean (SD), n	Weight	MD (95% CI)
Ozben (2016)	27.5 (14.0), 28	17.0 (9.1), 25	20.2%	10.50 (4.21 to 16.79)
Morelli (2018)	16.6 (8.0), 30	19.0 (8.0), 30	25.4%	−2.40 (−6.45 to 1.65)
Aliyev (2022)	26.1 (10.1), 62	25.7 (10.6), 88	27.0%	0.40 (−2.95 to 3.75)
Wang (2026)	16.8 (4.9), 55	15.3 (8.1), 31	27.5%	1.49 (−1.64 to 4.62)
**Pooled estimate**	**175**	**174**	**100%**	**2.02 (−6.24 to 10.29)**
Prediction interval				(−13.82 to 17.87)

Heterogeneity: $I^2 = 74\%$ (28 to 91%), $\tau^2 = 18.9$, $p = 0.009$

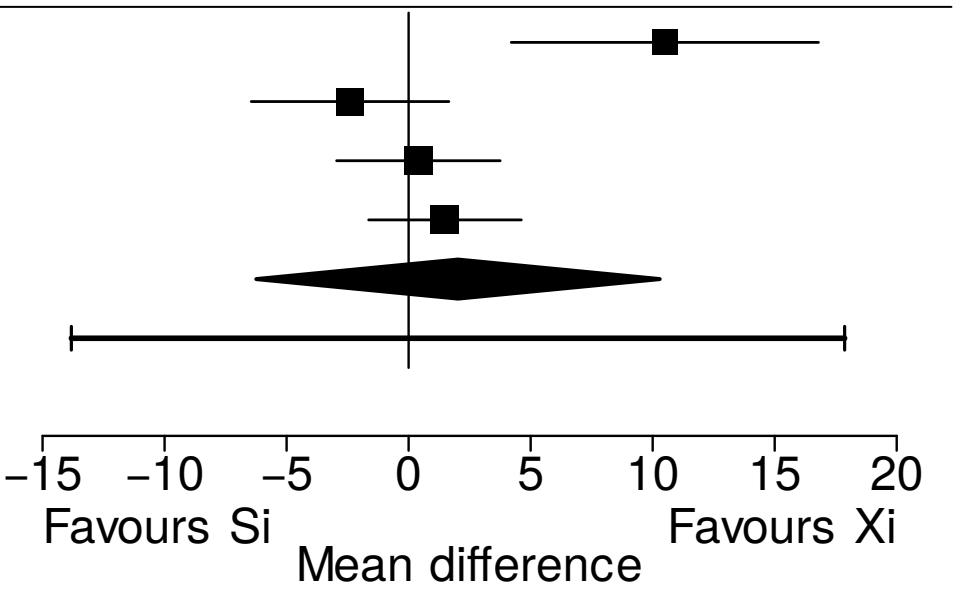

Supplementary Fig. 18 Lymph node yield, post hoc and exploratory: four-cohort set without the cohort whose arms differed in stage and procedure. Data-driven; not used for inference.

Lymph node yield, nodes; post hoc, without Ozben (2016)

Study	da Vinci Xi<br>Mean (SD), n	da Vinci Si<br>Mean (SD), n	Weight	MD (95% CI)
Morelli (2018)	16.6 (8.0), 30	19.0 (8.0), 30	24.3%	−2.40 (−6.45 to 1.65)
Aliyev (2022)	26.1 (10.1), 62	25.7 (10.6), 88	35.3%	0.40 (−2.95 to 3.75)
Wang (2026)	16.8 (4.9), 55	15.3 (8.1), 31	40.4%	1.49 (−1.64 to 4.62)
**Pooled estimate**	**147**	**149**	**100%**	**0.16 (−4.48 to 4.80)**
Prediction interval				(−4.31 to 4.64)

Heterogeneity: $I^2 = 11\%$ (0 to 91%), $\tau^2 = 0.0376$, $p = 0.325$

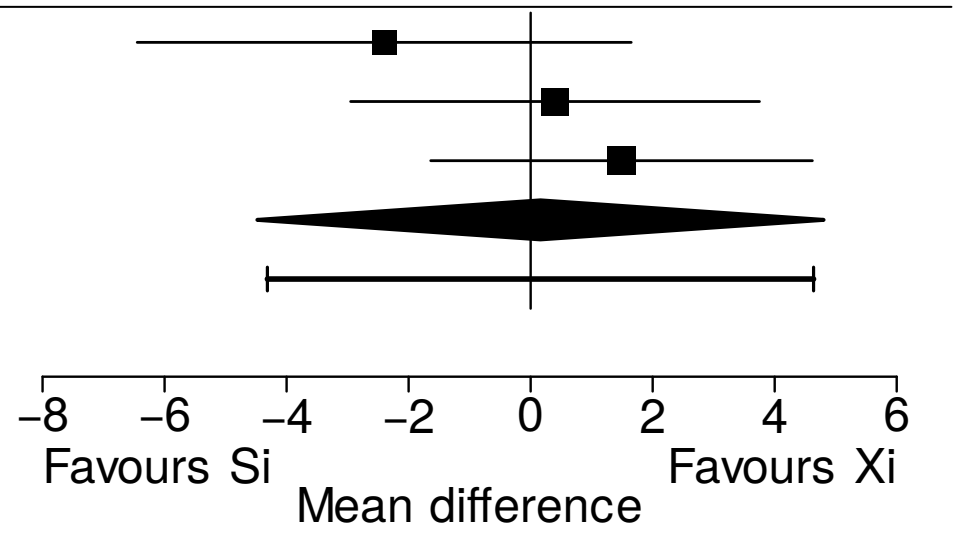

Supplementary Fig. 19 Overall postoperative complications, risk difference.

Overall postoperative complications; risk difference

**Study**	**da Vinci Xi** Events/total	**da Vinci Si** Events/total	**Weight**	**RD, % (95% CI)**
Ozben (2016)	4/28	3/25	13.7%	2.3 (−15.9 to 20.5)
Morelli (2018)	6/30	8/30	9.9%	−6.7 (−28.0 to 14.7)
Tung (2018)	5/24	5/33	10.9%	5.7 (−14.7 to 26.0)
Aliyev (2022)	7/62	9/88	44.3%	1.1 (−9.0 to 11.2)
Wang (2026)	4/55	5/31	21.1%	−8.9 (−23.5 to 5.8)
**Pooled estimate**	**199**	**207**	**100%**	**−1.1 (−8.0 to 5.7)**
Prediction interval				(−10.7 to 8.4)

Heterogeneity: $I^2 = 0\%$ (0 to 60%), $\tau^2 = 0$, $p = 0.722$

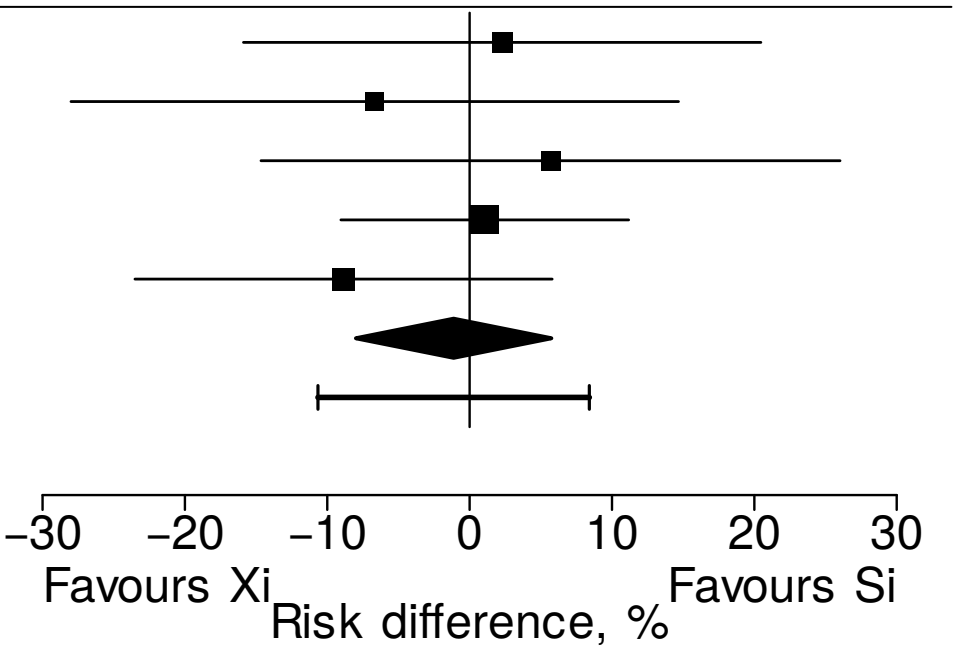

Supplementary Fig. 20 Overall postoperative complications, reported counts only.

Overall postoperative complications; reported counts only

Study	da Vinci Xi Events/total	da Vinci Si Events/total	Weight	RR (95% CI)
Ozben (2016)	4/28	3/25	14.8%	1.19 (0.29 to 4.81)
Morelli (2018)	6/30	8/30	33.3%	0.75 (0.30 to 1.90)
Aliyev (2022)	7/62	9/88	33.1%	1.10 (0.43 to 2.81)
Wang (2026)	4/55	5/31	18.8%	0.45 (0.13 to 1.56)
**Pooled estimate**	**175**	**174**	**100%**	**0.83 (0.44 to 1.57)**
Prediction interval				(0.35 to 1.98)

Heterogeneity: $I^2 = 0\%$ (0 to 71%), $\tau^2 = 0$, $p = 0.661$

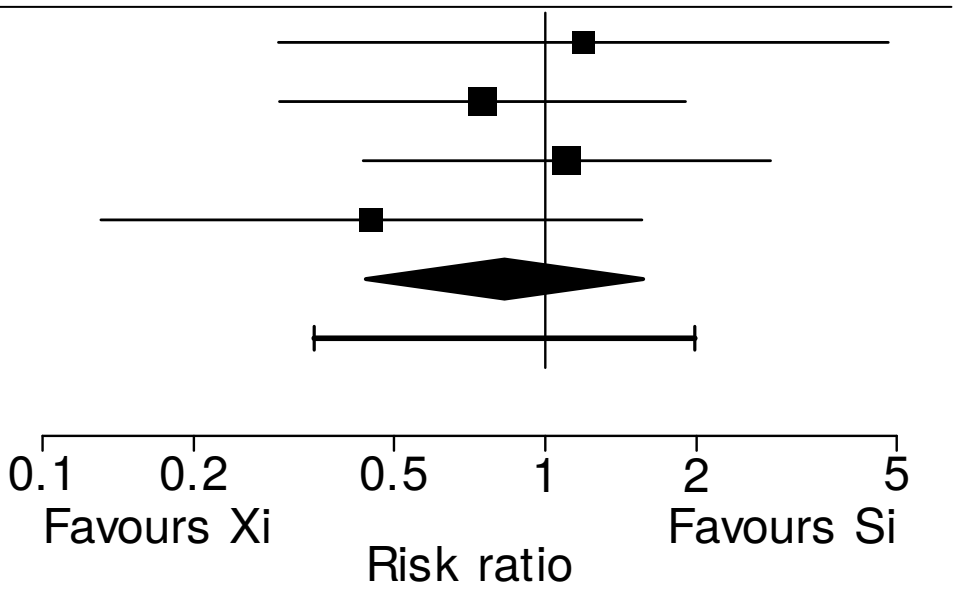

Supplementary Fig. 21 Anastomotic leak, widened to include the cohort reporting anastomotic complications.

Anastomotic leak; including the cohort reporting anastomotic complications

**Study**	**da Vinci Xi** Events/total	**da Vinci Si** Events/total	**Weight**	**RR (95% CI)**
Ozben (2016)	1/27	2/21	10.2%	0.39 (0.04 to 4.00)
Morelli (2018)	2/28	3/28	17.6%	0.67 (0.12 to 3.69)
Tung (2018)	2/24	5/33	20.6%	0.55 (0.12 to 2.60)
Panteleimonitis (2020)	0/99	4/109	6.8%	0.12 (0.01 to 2.24)
Wang (2026)	2/52	1/28	10.0%	1.08 (0.10 to 11.36)
Aliyev (2022)	7/62	5/88	34.7%	1.99 (0.66 to 5.97)
**Pooled estimate**	**292**	**307**	**100%**	**0.83 (0.33 to 2.05)**
Prediction interval				(0.20 to 3.41)

Heterogeneity: I^2 = 0% (0 to 74%), τ^2 = 0.144, p = 0.438

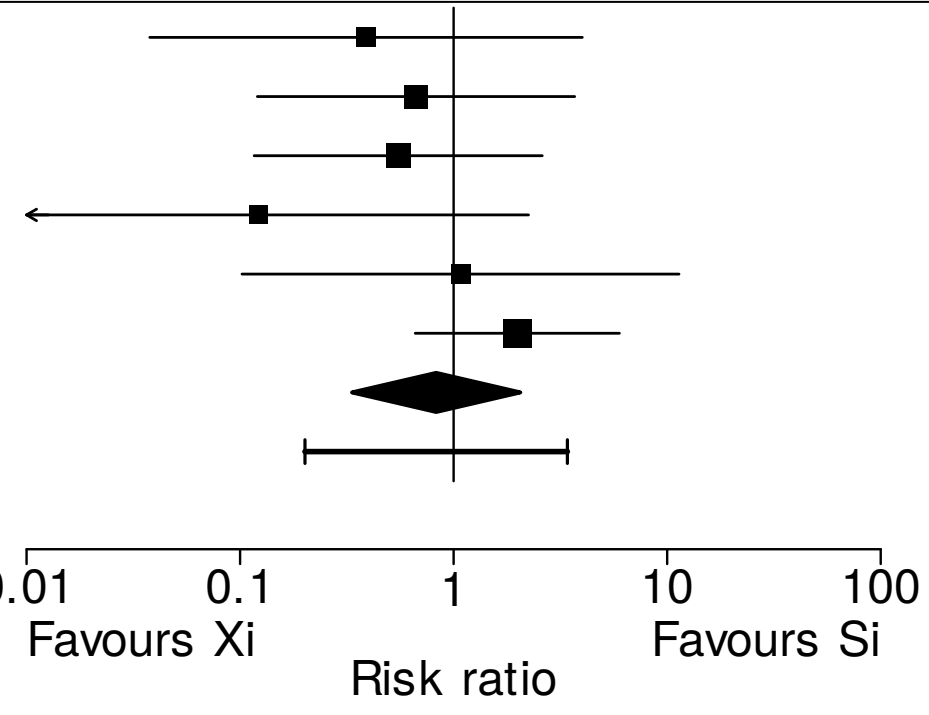

Supplementary Fig. 22 Anastomotic leak, Peto odds ratio.

Anastomotic leak; Peto odds ratio

**Study**	**da Vinci Xi** Events/total	**da Vinci Si** Events/total	**Weight**	**OR (95% CI)**
Ozben (2016)	1/27	2/21	14.0%	0.38 (0.04 to 3.89)
Morelli (2018)	2/28	3/28	23.0%	0.65 (0.11 to 4.01)
Tung (2018)	2/24	5/33	30.2%	0.54 (0.11 to 2.63)
Panteleimonitis (2020)	0/99	4/109	19.5%	0.14 (0.02 to 1.04)
Wang (2026)	2/52	1/28	13.2%	1.08 (0.10 to 11.92)
**Pooled estimate**	**230**	**219**	**100%**	**0.45 (0.19 to 1.09)**
Prediction interval				(0.13 to 1.56)

Heterogeneity: I^2 = 0% (0 to 59%), τ^2 = 0, p = 0.735

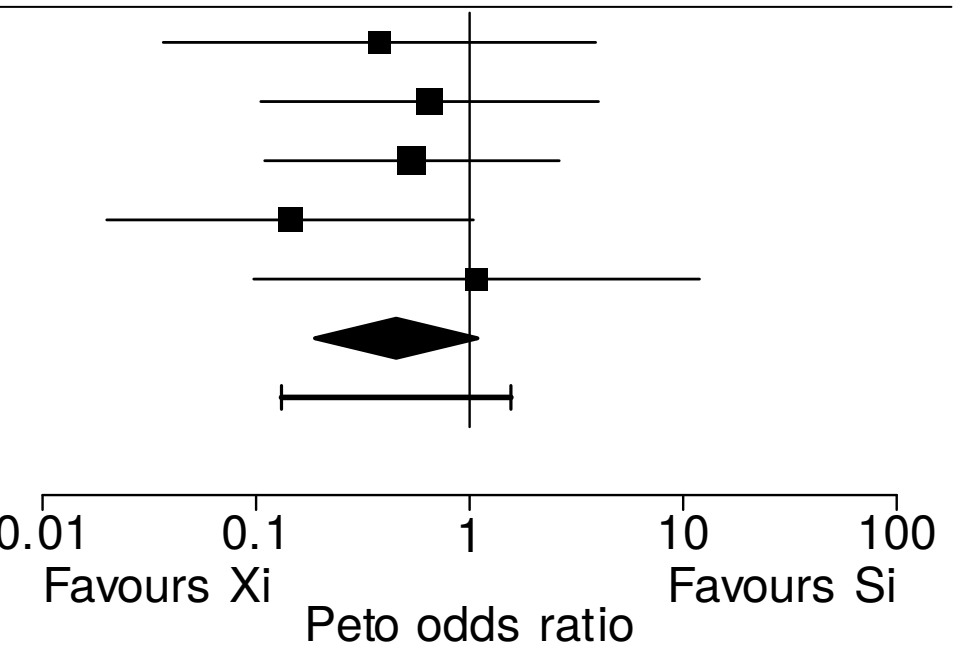

Supplementary Fig. 23 Anastomotic leak, risk difference.

Anastomotic leak; risk difference

**Study**	**da Vinci Xi** Events/total	**da Vinci Si** Events/total	**Weight**	**RD, % (95% CI)**
Ozben (2016)	1/27	2/21	5.3%	−5.8 (−20.3 to 8.6)
Morelli (2018)	2/28	3/28	5.0%	−3.6 (−18.5 to 11.3)
Tung (2018)	2/24	5/33	4.1%	−6.8 (−23.3 to 9.7)
Panteleimonitis (2020)	0/99	4/109	70.8%	−3.7 (−7.6 to 0.3)
Wang (2026)	2/52	1/28	14.8%	0.3 (−8.4 to 8.9)
**Pooled estimate**	**230**	**219**	**100%**	**−3.3 (−5.7 to −1.0)**
Prediction interval				(−8.0 to 1.4)

Heterogeneity: $I^2 = 0\%$ (0 to 16%), $\tau^2 = 0$, $p = 0.912$

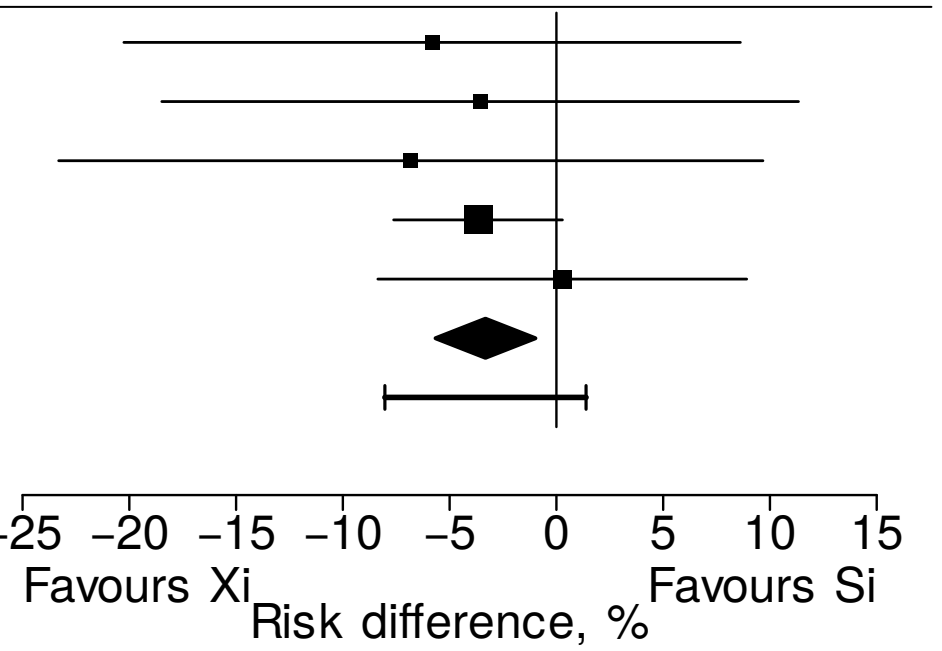

Supplementary Fig. 24 Anastomotic leak, smallest defensible denominators.

Anastomotic leak; smallest defensible denominators

**Study**	**da Vinci Xi** Events/total	**da Vinci Si** Events/total	**Weight**	**RR (95% CI)**
Ozben (2016)	1/27	2/21	14.8%	0.39 (0.04 to 4.00)
Morelli (2018)	2/28	3/28	27.6%	0.67 (0.12 to 3.69)
Tung (2018)	2/24	5/33	33.5%	0.55 (0.12 to 2.60)
Panteleimonitis (2020)	0/99	4/110	9.5%	0.12 (0.01 to 2.26)
Wang (2026)	2/52	1/27	14.6%	1.04 (0.10 to 10.94)
**Pooled estimate**	**230**	**219**	**100%**	**0.52 (0.25 to 1.12)**
Prediction interval				(0.15 to 1.87)

Heterogeneity: I^2 = 0% (0 to 41%), τ^2 = 0, p = 0.841

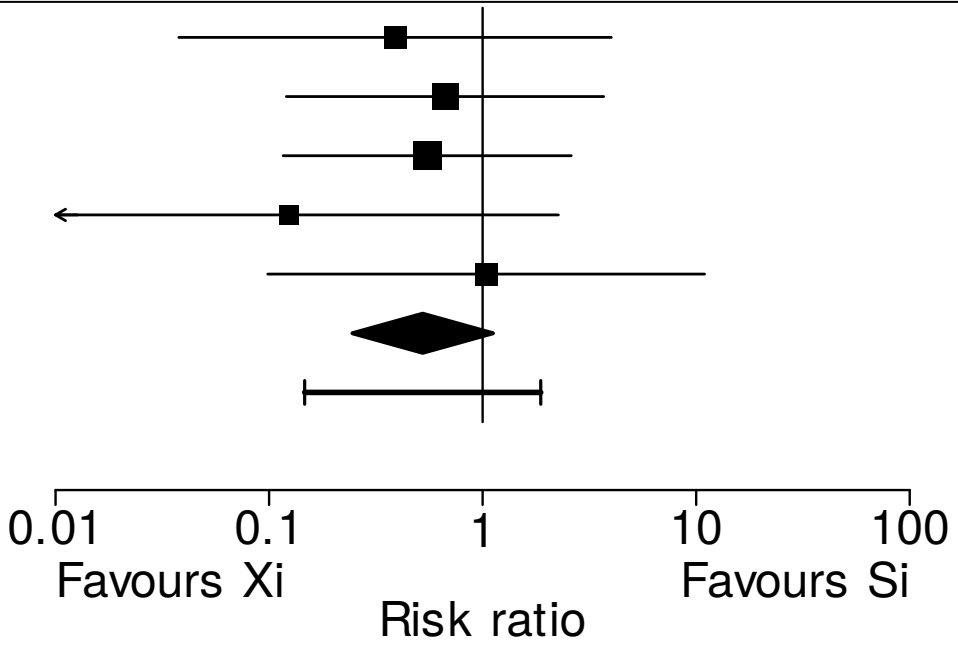

Supplementary Fig. 25 Conversion to open surgery, risk ratio (three informative cohorts; the analysis specified in the protocol).

Conversion to open surgery; risk ratio

**Study**	**da Vinci Xi** Events/total	**da Vinci Si** Events/total	**Weight**	**RR (95% CI)**
Ozben (2016)	1/28	1/25	36.5%	0.89 (0.06 to 13.54)
Morelli (2018)	1/30	1/30	36.4%	1.00 (0.07 to 15.26)
Tung (2018)	1/24	0/33	27.1%	4.10 (0.17 to 96.49)
Panteleimonitis (2020)	0/116	0/124	0%	No event in either arm
Aliyev (2022)	0/62	0/88	0%	No event in either arm
**Pooled estimate**	**260**	**300**	**100%**	**1.41 (0.19 to 10.29)**
Prediction interval				(0.04 to 51.88)

Heterogeneity: I^2 = 0% (0 to 66%), τ^2 = 0, p = 0.738

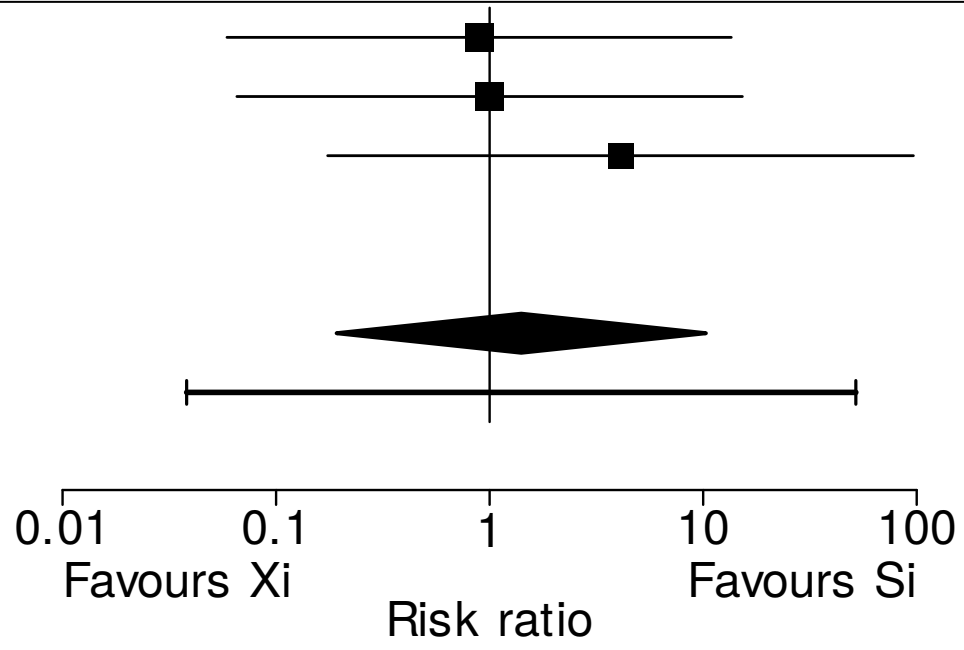

Supplementary Fig. 26 Conversion to open surgery, Peto odds ratio.

Conversion to open surgery; Peto odds ratio

**Study**	**da Vinci Xi** Events/total	**da Vinci Si** Events/total	**Weight**	**OR (95% CI)**
Ozben (2016)	1/28	1/25	39.9%	0.89 (0.05 to 14.70)
Morelli (2018)	1/30	1/30	40.2%	1.00 (0.06 to 16.37)
Tung (2018)	1/24	0/33	19.9%	10.75 (0.20 to 569.48)
Panteleimonitis (2020)	0/116	0/124	0%	No event in either arm
Aliyev (2022)	0/62	0/88	0%	No event in either arm
**Pooled estimate**	**260**	**300**	**100%**	**1.53 (0.08 to 29.57)**
Prediction interval				(0.03 to 74.86)

Heterogeneity: $I^2 = 0\%$ (0 to 82%), $\tau^2 = 0$, $p = 0.560$

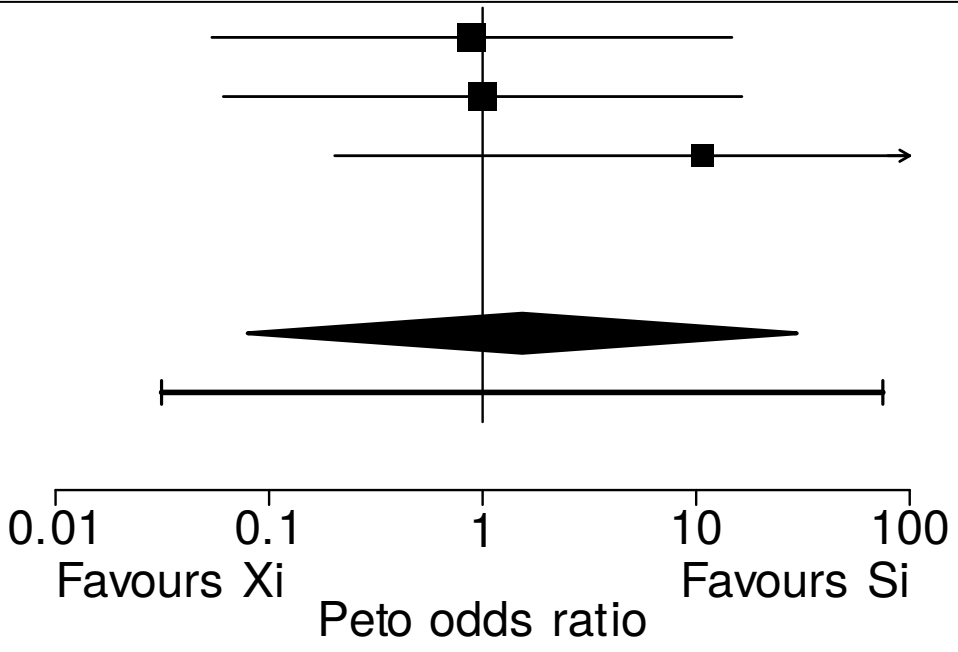

Supplementary Fig. 27 Reoperation, risk ratio (the analysis specified in the protocol).

Reoperation; risk ratio

**Study**	**da Vinci Xi** Events/total	**da Vinci Si** Events/total	**Weight**	**RR (95% CI)**
Ozben (2016)	1/28	2/25	18.1%	0.45 (0.04 to 4.63)
Morelli (2018)	1/30	0/30	9.9%	3.00 (0.13 to 70.78)
Panteleimonitis (2020)	8/116	4/124	72.0%	2.14 (0.66 to 6.91)
**Pooled estimate**	**174**	**179**	**100%**	**1.66 (0.25 to 11.22)**
Prediction interval				(0.19 to 14.80)

Heterogeneity: I^2 = 0% (0 to 86%), τ^2 = 0, p = 0.467

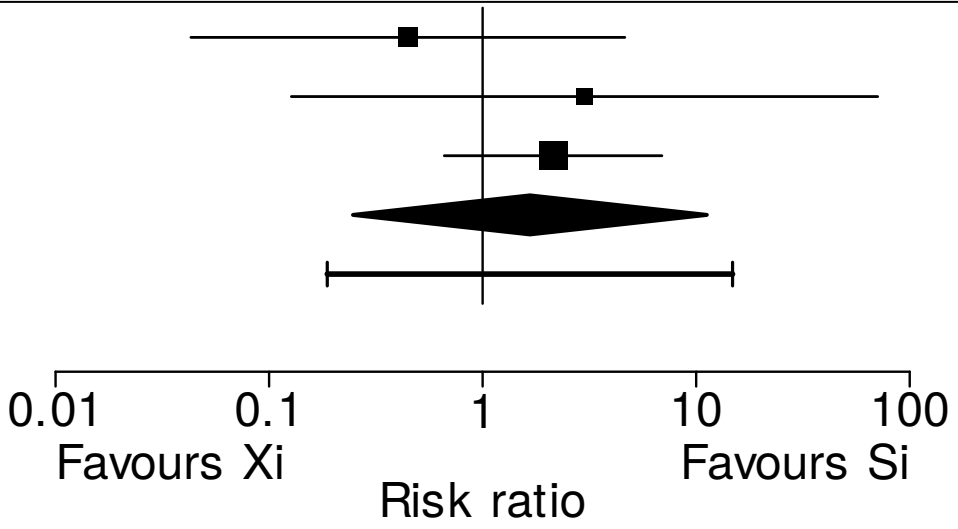

Supplementary Fig. 28 Reoperation, Peto odds ratio.

Reoperation; Peto odds ratio

**Study**	**da Vinci Xi** Events/total	**da Vinci Si** Events/total	**Weight**	**OR (95% CI)**
Ozben (2016)	1/28	2/25	18.8%	0.44 (0.04 to 4.47)
Morelli (2018)	1/30	0/30	6.5%	7.39 (0.15 to 372.38)
Panteleimonitis (2020)	8/116	4/124	74.7%	2.16 (0.68 to 6.88)
**Pooled estimate**	**174**	**179**	**100%**	**1.74 (0.19 to 15.67)**
Prediction interval				(0.19 to 15.67)

Heterogeneity: I^2 = 0% (0 to 90%), τ^2 = 0.000000459, p = 0.368

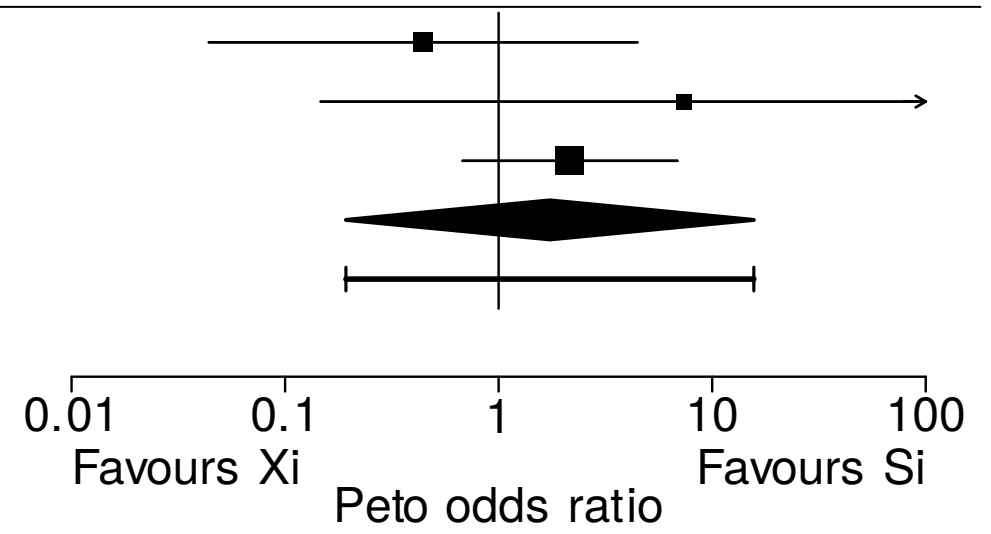

Supplementary Fig. 29 Circumferential resection margin involvement, retaining the result at critical risk of bias.

Circumferential resection margin involvement; retaining the critical-risk result

Study	da Vinci Xi Events/total	da Vinci Si Events/total	Weight	RR (95% CI)
Ozben (2016)	3/28	0/25	13.0%	6.26 (0.34 to 115.50)
Cha (2018)	1/36	1/55	14.7%	1.53 (0.10 to 23.66)
Morelli (2018)	0/30	0/30	0%	No event in either arm
Tung (2018)	0/24	0/33	0%	No event in either arm
Huang (2019)	2/29	6/77	46.5%	0.89 (0.19 to 4.14)
Aliyev (2022)	0/62	1/88	10.9%	0.47 (0.02 to 11.40)
Wang (2026)	1/55	1/31	14.8%	0.56 (0.04 to 8.70)
**Pooled estimate**	**264**	**339**	**100%**	**1.08 (0.38 to 3.10)**
Prediction interval				(0.24 to 4.80)

Heterogeneity: $I^2 = 0\%$ (0 to 58%), $\tau^2 = 0$, $p = 0.736$

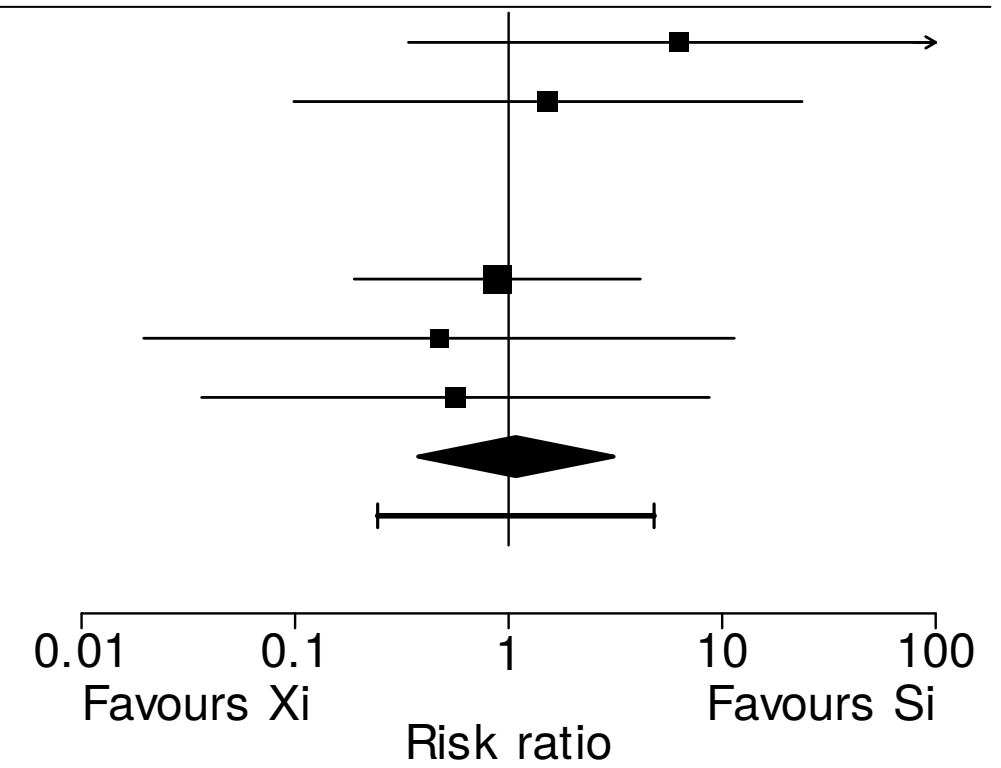

Supplementary Fig. 30 Circumferential resection margin involvement, Peto odds ratio (retaining the result at critical risk of bias).

Circumferential resection margin involvement; Peto odds ratio

**Study**	**da Vinci Xi** Events/total	**da Vinci Si** Events/total	**Weight**	**OR (95% CI)**
Ozben (2016)	3/28	0/25	21.3%	7.16 (0.71 to 72.26)
Cha (2018)	1/36	1/55	14.0%	1.56 (0.09 to 26.89)
Morelli (2018)	0/30	0/30	0%	No event in either arm
Tung (2018)	0/24	0/33	0%	No event in either arm
Huang (2019)	2/29	6/77	44.0%	0.88 (0.18 to 4.40)
Aliyev (2022)	0/62	1/88	7.2%	0.18 (0.00 to 9.73)
Wang (2026)	1/55	1/31	13.5%	0.54 (0.03 to 9.89)
**Pooled estimate**	**264**	**339**	**100%**	**1.25 (0.30 to 5.24)**
Prediction interval				(0.27 to 5.65)

Heterogeneity: $I^2 = 0\%$ (0 to 77%), $\tau^2 = 0$, $p = 0.461$

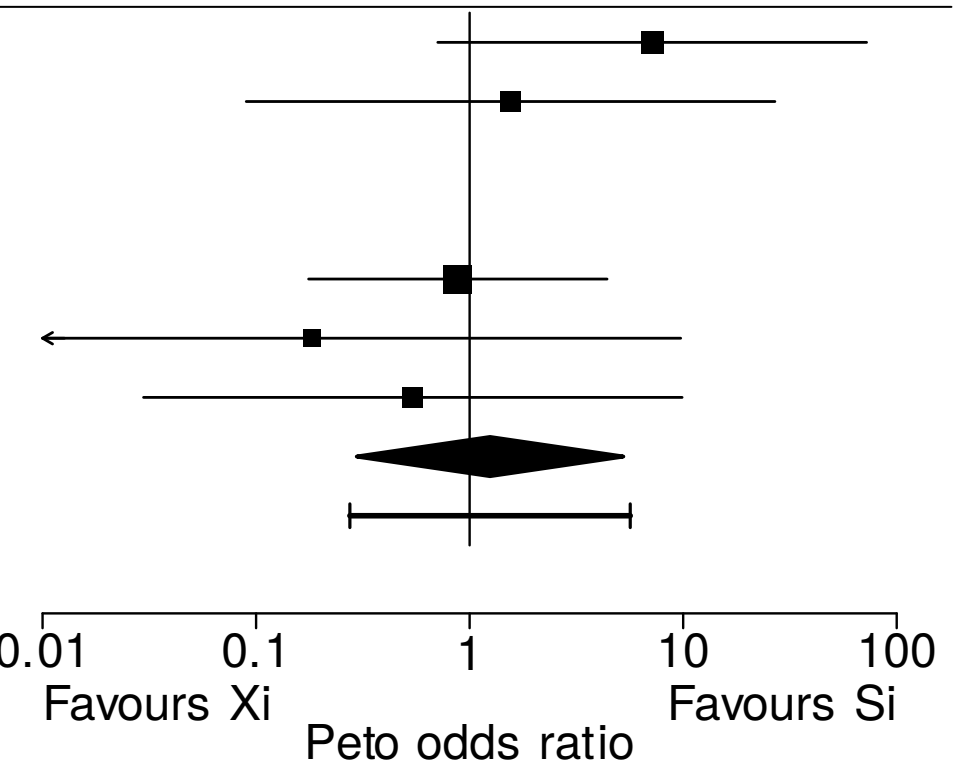

Supplementary Fig. 31 Circumferential resection margin involvement, rectal-only cohorts.

Circumferential resection margin involvement; rectal-only cohorts

**Study**	**da Vinci Xi** Events/total	**da Vinci Si** Events/total	**Weight**	**RR (95% CI)**
Ozben (2016)	3/28	0/25	24.4%	6.26 (0.34 to 115.50)
Cha (2018)	1/36	1/55	27.6%	1.53 (0.10 to 23.66)
Morelli (2018)	0/30	0/30	0%	No event in either arm
Tung (2018)	0/24	0/33	0%	No event in either arm
Aliyev (2022)	0/62	1/88	20.4%	0.47 (0.02 to 11.40)
Wang (2026)	1/55	1/31	27.6%	0.56 (0.04 to 8.70)
**Pooled estimate**	**235**	**262**	**100%**	**1.29 (0.20 to 8.17)**
Prediction interval				(0.12 to 13.31)

Heterogeneity: $I^2 = 0\%$ (0 to 76%), $\tau^2 = 0$, $p = 0.598$

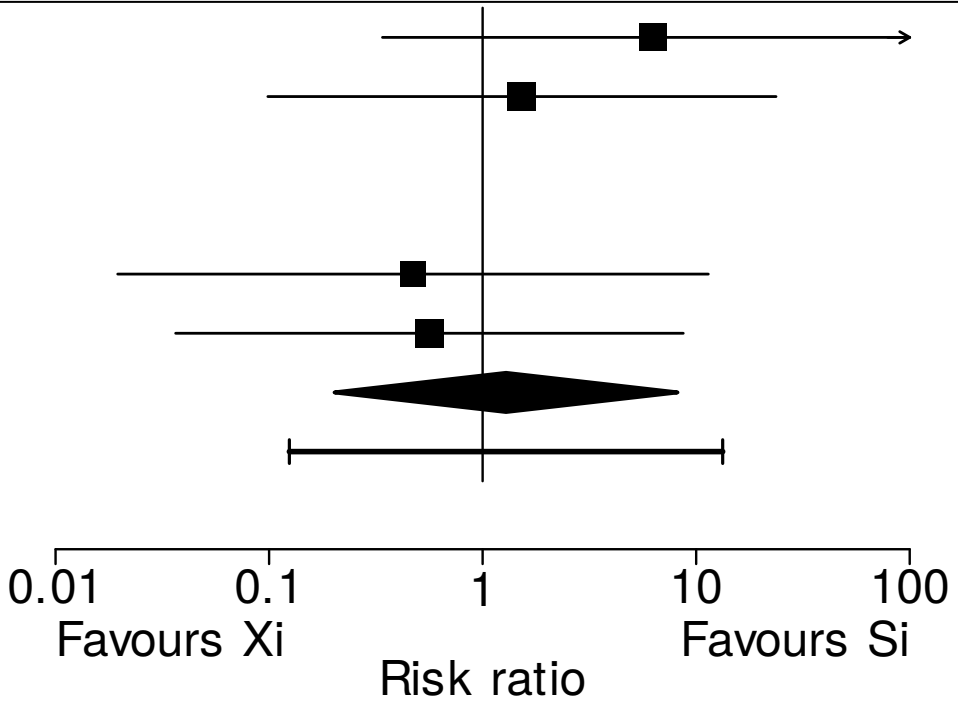

Supplementary Fig. 32 Mesorectal specimen quality, success-scale risk ratio (the measure specified in the protocol). Two cohorts with complete specimens in every patient carry most of the weight.

Mesorectal specimen complete or near-complete; success-scale risk ratio

Study	da Vinci Xi Events/total	da Vinci Si Events/total	Weight	RR (95% CI)
Ozben (2016)	23/23	19/19	18.9%	1.00 (0.91 to 1.10)
Morelli (2018)	30/30	30/30	39.4%	1.00 (0.94 to 1.07)
Aliyev (2022)	58/62	86/88	31.3%	0.96 (0.89 to 1.03)
Wang (2026)	53/55	28/31	10.4%	1.07 (0.94 to 1.21)
**Pooled estimate**	**170**	**168**	**100%**	**0.99 (0.94 to 1.05)**
Prediction interval				(0.93 to 1.06)

Heterogeneity: $I^2 = 0\%$ (0 to 80%), $\tau^2 = 0$, $p = 0.515$

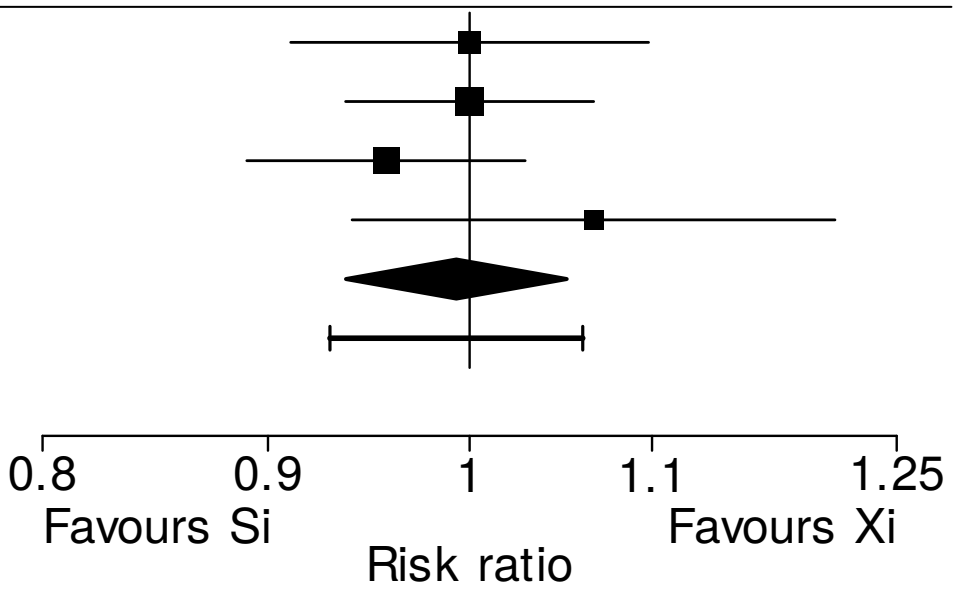

Supplementary Fig. 33 Mesorectal specimen quality on the failure scale, retaining the result at critical risk of bias; without it only two cohorts are informative.

Incomplete mesorectal specimen; failure-scale risk ratio retaining the critical-risk result

**Study**	**da Vinci Xi** Events/total	**da Vinci Si** Events/total	**Weight**	**RR (95% CI)**
Ozben (2016)	0/23	0/19	0%	No event in either arm
Cha (2018)	2/36	6/55	36.0%	0.51 (0.11 to 2.39)
Morelli (2018)	0/30	0/30	0%	No event in either arm
Aliyev (2022)	4/62	2/88	32.8%	2.84 (0.54 to 15.02)
Wang (2026)	2/55	3/31	31.2%	0.38 (0.07 to 2.13)
**Pooled estimate**	**206**	**223**	**100%**	**0.81 (0.06 to 11.91)**
Prediction interval				(0.02 to 40.71)

Heterogeneity: I² = 39% (0 to 81%), τ² = 0.444, p = 0.194

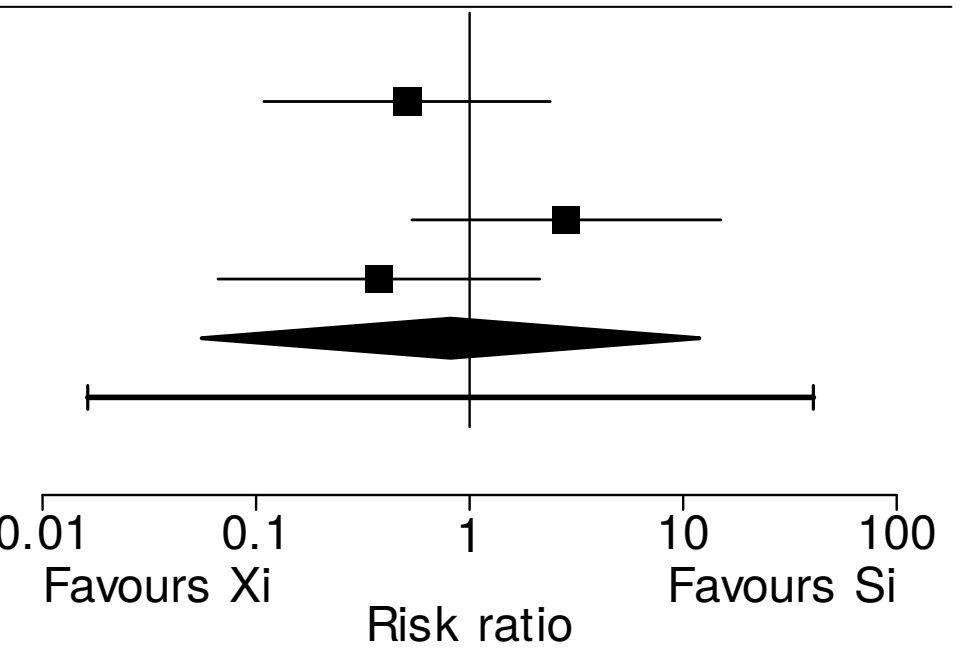

Supplement: Supplementary file 1 [file 11701_2026_4059_MOESM2_ESM.pdf]
