# Supplementary material for: Comparative perioperative and oncological outcomes of the da Vinci Xi versus Si robotic surgical systems in rectal cancer surgery: a systematic review and meta-analysis
Source: J Robot Surg. 2026 Oct 20;20(1):1115. doi: 10.1007/s11701-026-04059-0 (PMC13683476; doi:10.1007/s11701-026-04059-0)
Supplement: Supplementary file 2 [file 11701_2026_4059_MOESM3_ESM.pdf]

PRISMA 2020 Checklist

Comparative Perioperative and Oncological Outcomes of the da Vinci Xi Versus Si Robotic Surgical Systems in Rectal Cancer Surgery: A Systematic Review and Meta-analysis

Journal of Robotic Surgery

Ivens Murad Garcia Magalhães; Fillipe Augusto de Oliveira Paixão; Gustavo Tamura; Pedro Averbach

Corresponding author: Ivens Murad Garcia Magalhães, Faculdade de Medicina, Universidade Federal de Juiz de Fora, Avenida Presidente Itamar Franco, 2344, Juiz de Fora, Minas Gerais, Brazil. E-mail: garciaivens@gmail.com

This file is Online Resource 3. Revised version: the title and the locations of items have been updated to the revised manuscript.

Section and topic	Item	Checklist item	Location where item is reported
**TITLE**			
Title	1	Identify the report as a systematic review.	Title page
**ABSTRACT**			
Abstract	2	See the PRISMA 2020 for Abstracts checklist.	Abstract (structured: Purpose, Methods, Results, Conclusion, PROSPERO registration)
**INTRODUCTION**			
Rationale	3	Describe the rationale for the review in the context of existing knowledge.	Introduction, paragraphs 1–3
Objectives	4	Provide an explicit statement of the objective(s) or question(s) the review addresses.	Introduction, final paragraph; Abstract, Purpose
**METHODS**			
Eligibility criteria	5	Specify the inclusion and exclusion criteria for the review and how studies were grouped for the syntheses.	Methods, Eligibility criteria
Information sources	6	Specify all databases, registers, websites, organisations, reference lists and other sources searched or consulted, and the date when each was last searched or consulted.	Methods, Literature search; Online Resource 1, Section S3 and Supplementary Table 1
Search strategy	7	Present the full search strategies for all databases, registers and websites, including any filters and limits used.	Online Resource 1, Supplementary Table 1
Selection process	8	Specify the methods used to decide whether a study met the inclusion criteria, including how many reviewers screened each record and each report, whether they worked independently, and details of automation tools used.	Methods, Study selection and data extraction
Data collection process	9	Specify the methods used to collect data from reports, including how many reviewers collected data from each report, whether they worked independently, any processes for obtaining or confirming data from investigators, and details of automation tools used.	Methods, Study selection and data extraction (including author correspondence)
Data items	10a	List and define all outcomes for which data were sought.	Methods, Statistical analysis; Table 2; Online Resource 1, Section S2 and Supplementary Table 6

Section and topic	Item	Checklist item	Location where item is reported
	10b	List and define all other variables for which data were sought.	Methods, Study selection and data extraction; Table 1; Online Resource 1, Supplementary Table 7
Study risk of bias assessment	11	Specify the methods used to assess risk of bias, including details of the tool(s) used, how many reviewers assessed each study and whether they worked independently.	Methods, Risk of bias and certainty of evidence; Online Resource 1, Supplementary Table 4
Effect measures	12	Specify for each outcome the effect measure(s) used in the synthesis or presentation of results.	Methods, Statistical analysis; Tables 2 and 3
Synthesis methods	13a	Describe the processes used to decide which studies were eligible for each synthesis.	Methods, Statistical analysis
	13b	Describe any methods required to prepare the data for presentation or synthesis.	Methods, Statistical analysis (median-to-mean conversion); Methods, Study selection and data extraction (derived-value flagging)
	13c	Describe any methods used to tabulate or visually display results of individual studies and syntheses.	Figures 2 and 3; Tables 2 and 3
	13d	Describe any methods used to synthesise results and provide a rationale for the choice(s), including any methods used to identify the presence and extent of statistical heterogeneity.	Methods, Statistical analysis; Online Resource 1, Section S2
	13e	Describe any methods used to explore possible causes of heterogeneity among study results.	Methods, Statistical analysis; Results, Operative efficiency; Online Resource 2, Supplementary Fig. 2
	13f	Describe any sensitivity analyses conducted to assess robustness of the synthesised results.	Methods, Statistical analysis; Table 2
Reporting bias assessment	14	Describe any methods used to assess risk of bias due to missing results in a synthesis.	Methods, Risk of bias and certainty of evidence; Results, Outcomes that could not be pooled, and certainty
Certainty assessment	15	Describe any methods used to assess certainty (or confidence) in the body of evidence for an outcome.	Methods, Risk of bias and certainty of evidence; Table 3; Online Resource 1, Section S1
**RESULTS**			
Study selection	16a	Describe the results of the search and selection process, from the number of records identified in the search to the number of studies included in the review, ideally using a flow diagram.	Results, Study selection; Figure 1
	16b	Cite studies that might appear to meet the inclusion criteria, but which were excluded, and explain why they were excluded.	Results, Study selection; Online Resource 1, Section S5 and Supplementary Table 2
Study characteristics	17	Cite each included study and present its characteristics.	Table 1; Online Resource 1, Supplementary Tables 6 and 7
Risk of bias in studies	18	Present assessments of risk of bias for each included study.	Results, Risk of bias; Online Resource 2,

Section and topic	Item	Checklist item	Location where item is reported
			Supplementary Fig. 1; Online Resource 1, Supplementary Table 4
Results of individual studies	19	For all outcomes, present, for each study, summary statistics for each group and an effect estimate and its precision, ideally using structured tables or plots.	Figure 2; Figure 3; Online Resource 1, Supplementary Table 3
Results of syntheses	20a	For each synthesis, briefly summarise the characteristics and risk of bias among contributing studies.	Results, Characteristics of included studies and Risk of bias; Table 1
	20b	Present results of all statistical syntheses conducted, including summary estimates and their precision and measures of statistical heterogeneity.	Table 2; Figures 2 and 3; Results
	20c	Present results of all investigations of possible causes of heterogeneity among study results.	Results, Operative efficiency and Oncological and pathological outcomes; Online Resource 2, Supplementary Fig. 2
	20d	Present results of all sensitivity analyses conducted to assess the robustness of the synthesised results.	Table 2; Online Resource 2, Supplementary Figs 3–33; Online Resource 4
Reporting biases	21	Present assessments of risk of bias due to missing results for each synthesis assessed.	Results, Outcomes that could not be pooled, and certainty (not assessable below ten cohorts; stated explicitly); Table 3
Certainty of evidence	22	Present assessments of certainty (or confidence) in the body of evidence for each outcome assessed.	Table 3; Online Resource 1, Supplementary Table 5
**DISCUSSION**			
Discussion	23a	Provide a general interpretation of the results in the context of other evidence.	Discussion, opening paragraph and Pathological outcomes, safety signals and previous syntheses
	23b	Discuss any limitations of the evidence included in the review.	Discussion, The operative-time finding depends on one exclusion; Limitations
	23c	Discuss any limitations of the review processes used.	Limitations
	23d	Discuss implications of the results for practice, policy and future research.	Discussion, Who this comparison informs; Implications and future research; Conclusion
**OTHER INFORMATION**			
Registration and protocol	24a	Provide registration information for the review, including register name and registration number, or state that the review was not registered.	Title page; Abstract, PROSPERO registration; Methods, Protocol and registration
	24b	Indicate where the review protocol can be accessed, or state that a protocol was not prepared.	Methods, Protocol and registration
	24c	Describe and explain any amendments to information provided at registration or in the protocol.	Methods, Protocol and registration; Online Resource 1, Sections S5 and S7
Support	25	Describe sources of financial or non-financial support for the review, and the role of the funders or sponsors in the review.	Title page; Declarations
Competing interests	26	Declare any competing interests of review authors.	Title page; Declarations

Section and topic	Item	Checklist item	Location where item is reported
Availability of data, code and other materials	27	Report which of the following are publicly available and where they can be found: template data collection forms; data extracted from included studies; data used for all analyses; analytic code; any other materials used in the review.	Online Resource 1, Supplementary Table 3 (all data extracted from the included studies); Online Resource 4 (the analysis datasets, every result table and the complete analysis code); Declarations, Data availability and Code availability. The data collection form is described in Methods, Study selection and data extraction

From: Page MJ, McKenzie JE, Bossuyt PM, Boutron I, Hoffmann TC, Mulrow CD, et al. The PRISMA 2020 statement: an updated guideline for reporting systematic reviews. BMJ 2021;372:n71. doi:10.1136/bmj.n71
