# Supplementary material for: Comparative perioperative and oncological outcomes of the da Vinci Xi versus Si robotic surgical systems in rectal cancer surgery: a systematic review and meta-analysis
Source: J Robot Surg. 2026 Oct 20;20(1):1115. doi: 10.1007/s11701-026-04059-0 (PMC13683476; doi:10.1007/s11701-026-04059-0)
Supplement: Supplementary file 3 [file 11701_2026_4059_MOESM4_ESM.pdf]

Supplementary Material

Comparative Perioperative and Oncological Outcomes of the da Vinci Xi Versus Si Robotic Surgical Systems in Rectal Cancer Surgery: A Systematic Review and Meta-analysis

Journal of Robotic Surgery

Ivens Murad Garcia Magalhães; Fillipe Augusto de Oliveira Paixão; Gustavo Tamura; Pedro Averbach

Corresponding author: Ivens Murad Garcia Magalhães, Faculdade de Medicina, Universidade Federal de Juiz de Fora, Avenida Presidente Itamar Franco, 2344, Juiz de Fora, Minas Gerais, Brazil. E-mail: garciaivens@gmail.com

This file is Online Resource 1. Revised version, submitted in response to peer review; every change from the submitted version is listed in Section S7.

Contents

Section S1. Rationale for the GRADE upgrading decisions.

Section S2. Outcome definitions, denominators and analysis choices requiring explanation.

Section S3. Search execution notes, screening chronology and the supplementary search.

Section S4. Publications linked to included cohorts.

Section S5. Eligibility revision applied after data extraction.

Section S6. Relationship to a previously registered review.

Section S7. Deviations from the protocol, changes made in response to peer review, and corrections to the submitted version.

Supplementary Table 1. Full search strategies as executed, for all five databases and for Google Scholar, with platform, fields, filters, date of execution, retrieval count and, for Google Scholar, the screening rule applied.

Supplementary Table 2. The fourteen reports assessed at full text and excluded, with the route by which each was identified and the reason for exclusion.

Supplementary Table 3. Complete outcome-level extraction: every value entering every analysis, with the outcome definition, the denominator, and a flag identifying values obtained by arithmetic from reported figures, or supplied by a study author, rather than printed in the source.

Supplementary Table 4. ROBINS-I V2 assessment of all 23 study results: panel A, the domain-level and overall judgements; panel B, the supporting evidence and rationale for each of the 138 domain judgements; panel C, the screening step.

Supplementary Table 5. GRADE evidence profile with the judgement and its reason for every certainty domain, for all ten graded outcomes.

Supplementary Table 6. Outcome definitions as stated by each cohort: operative, docking and console time, length of stay, complications, anastomotic leak and conversion.

Supplementary Table 7. Timing, centres and surgeons of each platform arm, the relation between the arms, co-interventions reported by platform, and possible overlap between cohorts.

Other Online Resources. Online Resource 2 contains Supplementary Figs 1 to 33: the risk-of-bias summary, the exploratory operative-time display, seven leave-one-out analyses and twenty-four sensitivity analyses. Online Resource 3 contains the PRISMA 2020 checklist. Online Resource 4 contains the analysis datasets as comma-separated files, every result table, and the complete R script that reproduces every estimate and every figure in this review from them.

S1. Rationale for the GRADE upgrading decisions

Upgrading was assessed for every graded outcome and applied to none. The reasoning is recorded here in full because it is uniform across outcomes and is referenced from the Results. Because every result was assessed with ROBINS-I, certainty started at high and was rated down for risk of bias, two levels for serious and three for very serious, before any other domain was considered (reference 11 of the main text).

Large magnitude of effect. This criterion was excluded by pre-specification for the operative-efficiency outcomes. A large difference in operative or docking time between two calendar periods, or between two centres, is as consistent with accumulating team experience or with centre as with a platform effect, so the magnitude of the observed difference carries no additional information about causation in this body of evidence. For the dichotomous outcomes no estimate reached the conventional threshold for upgrading on magnitude in an analysis whose confidence interval excluded a null or trivial effect.

Dose-response gradient. No such gradient can exist for a binary exposure. The platform is either the Xi or the Si; there is no intermediate dose and no ordered exposure to examine.

Plausible residual confounding working against the observed effect. This criterion allows upgrading where confounding would be expected to have masked a real effect. In the four cohorts in which the Xi replaced the Si within the same team, the dominant confounder, calendar period and the accumulated experience that travels with it, runs in the same direction as the Xi wherever the Xi appears better, because the Xi arm was the later-operated arm. In the most recent cohort both platforms were used concurrently only after an initial period of Si use alone, so the same bias applies in attenuated form, and two cohorts did not report the timing of their arms. The exception is the multicentre cohort, in which platform coincided with centre and whose investigators state that their Xi arm included twenty training cases and was operated by surgeons early in their robotic experience; there, confounding would be expected to work against the Xi. Because the direction of residual confounding differs between cohorts, and in most of them favours the Xi, the criterion is not met for any outcome (Supplementary Table 7).

S2. Outcome definitions, denominators and analysis choices requiring explanation

The definition that each cohort gives for each outcome, or the absence of one, is tabulated in Supplementary Table 6. This section records only the decisions that those definitions and denominators required.

Anastomotic leak

The denominator is the number of patients who actually received an anastomosis, not the arm size. Patients undergoing abdominoperineal resection, Hartmann's procedure or panproctocolectomy cannot develop an anastomotic leak and were removed from the denominator of both arms of every contributing cohort. Two denominators cannot be established exactly from the published record and are handled as follows, with the alternative values carried in a pre-specified sensitivity analysis.

Cohort	Denominator used	Basis and residual ambiguity
Panteleimonitis 2020	Xi 99, Si 109	Arm sizes less abdominoperineal excision (17 and 12), Hartmann's (0 and 2) and panproctocolectomy (0 and 1), from Table 3. The paper's own printed leak rate of 3.6% is not reproducible from 124 but is from a denominator near 110, confirming that the authors themselves used an anastomosis-only denominator without stating it. The Si value would be 110 if the single panproctocolectomy was restorative, which the paper does not state.
Wang 2026	Xi 52, Si 28	Arm sizes less Miles procedure (3 and 3), from Table 1. Table 8 reports four Si patients without anus preservation in the sub-5 cm subgroup alone, which exceeds the three Miles procedures reported for the whole Si arm, so the Si denominator could be 27. Sensitivity analysis uses 27.

Ozben 2016 (Xi 27, Si 21) and Morelli 2018 (Xi 28, Si 28) are determined exactly from the printed procedure tables; Tung 2018 performed low anterior resection with covering ileostomy in every patient, so its denominators are unchanged at 24 and 33.

Aliyev 2022 heads its counts anastomotic complications in its Table 2 and calls the same counts anastomotic leaks in its Results text, and defines neither. Because the table label admits anastomotic events other than leak, the cohort enters only the sensitivity analysis with the wider definition, as in the submitted version; the reason is now stated in full.

Operative time

Three cohorts define operative time as skin incision to closure; the others do not define it. Panteleimonitis 2020 did not record operative time at its Portuguese centre, all of whose 36 patients were in the Xi arm, so the medians and interquartile ranges in that report describe 80 Xi and 124 Si patients; the converted Xi arm enters the analyses with 80 patients, and the analysis with the whole arm of 116, as entered in the submitted version, is retained as a sensitivity analysis. Aliyev 2022 does not define its timing endpoint: its Table 2 heads the variable operative time, its Table 3 heads the same analysis console time, and its Methods define neither, while the Xi mean of 124 minutes is shorter than the console times reported by two other cohorts. This could not be resolved from the published record, and a sensitivity analysis omits the cohort.

Docking time

One cohort defines docking time, as the time from moving the robot into the surgical field to setting the robotic arms into the port sites; the other two do not define it. Absolute times differ about threefold between cohorts (Xi arms 5.3, 17.5 and 8.8 minutes), and some procedures required the robot to be docked twice (redocking with the Si in one cohort, dual docking with the Xi in another), so a single docking episode is not the unit in every cohort. The methods of the submitted version described all continuous outcomes as measured on the same scale; for docking time that was not established. The three estimates are therefore presented study by study, with the direction of effect counted and tested with a sign test, and are not pooled; the pooled mean difference and the standardised mean difference are shown in Online Resource 2 for completeness only.

Overall postoperative complications

Four definitions of the complication composite are in use across the five contributing cohorts. Only one cohort states an ascertainment window, of 30 days; the others state none. The definitions also differ in whether medical as well as surgical events are counted, not in whether the event is a complication. The label of 30 days or less attached to this outcome in the submitted version was therefore inaccurate and has been removed.

Outcomes reported but not pooled

Major complications of Clavien–Dindo grade III or above and diverting stoma formation are each reported by two cohorts once the study excluded on population grounds is set aside, which is below the three-cohort minimum fixed in the protocol. Both are described narratively in the main text. For major complications the counts of one cohort were supplied by its corresponding author and are not printed in the publication; they are now labelled author-supplied in Supplementary Table 3, where the submitted version labelled them reported. A second cohort reports no Clavien–Dindo category above grade III, so its count equals grade III or above only if no grade IV or V event occurred and went unreported; both facts are recorded in Supplementary Table 3.

Conversion to open surgery

Five conversions occurred among 560 patients. The protocol specified the risk ratio, which cannot use the two cohorts in which no conversion occurred in either arm, together 390 patients, so it rests on three informative cohorts and 170 patients. In response to peer review the risk difference, which retains all five cohorts and all 560 patients, is now the primary summary, and the risk ratio, the Peto odds ratio and a binomial-normal generalised linear mixed model are sensitivity analyses. None of the ratio estimates is informative, and the small absolute difference reflects the rarity of the event in both arms rather than evidence of equivalence. The definitions of conversion differ between cohorts (Supplementary Table 6). The same approach is applied to reoperation, 16 events in three cohorts.

Reoperation

One cohort states a 30-day window for reoperation; one lists reoperation among outcomes recorded within 30 days, where the scope of that qualifier is not explicit; and one states no window. The label of 30 days or less attached to this outcome in the submitted version has been removed.

Mesorectal specimen quality

The protocol specified the risk ratio on the success scale, complete or near-complete grading. More than 95 per cent of specimens were complete, with eleven incomplete specimens, and two of the four contributing cohorts had no incomplete specimen in either arm. On the success scale those two cohorts carry most of the weight and make the estimate look precise; on the failure scale they contribute no information, leaving two informative cohorts, below the three-cohort minimum. In response to peer review the risk difference, which retains all four cohorts, is now the primary summary. The success-scale risk ratio, the failure-scale risk ratio, which has to retain the result at critical risk of bias to reach three informative cohorts and nineteen events, and a binomial-normal model are sensitivity analyses.

Model used for the dichotomous outcomes

The submitted version described the dichotomous analyses as Mantel–Haenszel risk ratios without a zero-cell correction. That description was inaccurate. Every primary estimate comes from a random-effects model that pools study-level log risk ratios, or risk differences, by inverse variance, with a restricted maximum-likelihood estimate of between-study variance and a Hartung–Knapp interval; the software adds 0.5 to the cells of a study with a zero cell, and a study with no event in either

arm contributes no information to a ratio estimate. The Mantel–Haenszel estimate without correction is the common-effect estimate reported in Online Resource 4. The description has been corrected; no estimate changed because of it. Because τ^2 was estimated as zero for every dichotomous outcome, the interval with the ad hoc variance correction is reported alongside and used for interpretation where the two intervals differ in whether they include no effect.

S3. Search execution notes, screening chronology and the supplementary search

Database searches

Searches were executed on 15 August 2026 (Embase, Scopus) and 16 August 2026 (PubMed/MEDLINE, Web of Science Core Collection, Cochrane CENTRAL), from inception, with no date, language, publication-type or study-design restriction. The Cochrane CENTRAL search was re-executed on 16 August 2026 after a field-qualifier defect was identified in the original strategy; the corrected export (n = 24) is a strict subset of the original (n = 30) and none of the six dropped records was eligible. The PubMed export was repeated in MEDLINE format to obtain abstracts; the strategy was unchanged and both exports contained 364 records. The strategies as executed are reproduced verbatim in Supplementary Table 1.

Chronology of screening and registration

The database searches and the screening of titles and abstracts were completed on 15 and 16 August 2026. The review was registered in PROSPERO on 18 August 2026 (CRD420261480007), before data extraction. Registration therefore followed the searches and title-and-abstract screening, and the submitted version's description of the registration as prospective has been replaced by this chronology in the main text. The eligibility criteria, outcomes and analysis plan were fixed in the registered record before any data were extracted.

One internal anomaly in the Cochrane CENTRAL search is recorded rather than omitted: line #8, the union of lines #5, #6 and #7, returns 9,223, exactly the count of line #7 alone. The arithmetic of the remaining lines is internally consistent, line #9 at 249 records is the limiting line, and line #10 returns the 24 records that were exported and imported into deduplication.

Trial registries were not searched. The protocol listed a registry search among the supplementary methods; it was not executed, and no registry record contributes to this review. Consequently the review cannot state that no randomised trial comparing the platforms exists, and the statement to that effect in the submitted version has been removed. No randomised trial was identified by the database searches, which included the Cochrane Central Register of Controlled Trials.

Citation searching

Citation searching covered the reference lists of five reports and the citation graphs of six seed reports; 120 citing records were counted for four seeds, and no count was recorded for the other two. Five included cohorts could not be forward-searched individually because they are not indexed in the citation graphs used or are too recent to have accumulated citations. The citation searching is therefore not claimed to be comprehensive.

Google Scholar

Google Scholar was pre-specified in the protocol and in the PROSPERO record as a supplementary discovery source. Simple keyword searches were run on 20–21 August 2026. The registered record specifies screening the first 100 to 200 hits of each strategy; the lower bound of that pre-specified range was applied, and the first 100 records of each of the five strategies, about 500 records, were screened at title and abstract level. The stopping rule was therefore fixed before the search was executed and the more conservative value within it was used. Google Scholar returns no stable, reproducible total retrieval count, so no total is reported for that source in the flow diagram; the number of records screened is fixed and is now shown in the flow diagram. The supplementary search and the citation searching together yielded ten further reports for full-text assessment, of which nine were excluded and one proved to be an additional report of an already-included cohort, a letter by its investigators. Neither identified a study eligible for inclusion that the database searches had missed.

S4. Publications linked to included cohorts

The unit of synthesis is the independent cohort. Eight cohorts are reported in eighteen publications. One cohort accounts for nine of those publications; two further cohorts have one linked report each. An index publication was pre-specified for every cohort, and only the index publication contributes data to any analysis, so no patient contributes twice. Linked reports were read for supplementary information only: the platform-transition date, the cost analysis, the functional outcomes and the learning-curve analysis. The index publication of each cohort is the one cited for that cohort in Table 1 of the main text; the linked reports are cited alongside it in the study-selection paragraph of the Results. Overlap between different cohorts, as distinct from multiple reports of one cohort, is assessed in Supplementary Table 7: it cannot be excluded for the Si arms of the two Istanbul cohorts, and no overlap was found for the multicentre cohort.

S5. Eligibility revision applied after data extraction

One cohort was carried through data extraction and the first round of analysis before being excluded on population grounds. The reason, the evidence for it and its consequences are recorded here in full. In response to peer review this deviation is now also described in the Methods of the main text, and the PROSPERO record was amended to record it.

The finding

The cohort classifies tumour location by the Japanese Society for Cancer of the Colon and Rectum scheme, in which the rectum is subdivided into RS (rectosigmoid), Ra (upper rectum), Rb (lower rectum) and P (anal canal). Forty-eight of its 122 patients, 39.3 per cent, are RS. Location is also the only baseline variable on which its two arms differ significantly, the RS proportion being 48.3 per cent in the da Vinci Si arm and 30.6 per cent in the da Vinci Xi arm. Every outcome in the publication is reported for the whole arm; the single subgroup presented is defined by inflammatory markers, not by tumour location. No rectal-specific estimate can therefore be recovered from the published record.

The rule applied

The registered protocol admits mixed populations only where rectal-cancer-specific data can be separately identified and extracted. This was the only included cohort with a declared rectosigmoid component, so applying the rule was not a selective decision.

Direction of the change

The excluded cohort favoured the da Vinci Xi on every perioperative outcome it reported, several of them at conventional significance. Removing it therefore removes the most Xi-favourable cohort in the review, and the exclusion is conservative with respect to every conclusion drawn here.

What changed

The evidence base fell from nine cohorts and 965 patients to eight cohorts and 843 patients, and from nineteen to eighteen reports. Risk-of-bias assessment fell from 26 to 23 study results. Two outcomes fell below the three-cohort minimum and are now reported narratively rather than pooled: major complications of Clavien–Dindo grade III or above, and diverting stoma formation. Anastomotic leak, overall complications and conversion to open surgery were re-estimated. Operative time, docking time, length of stay, lymph node yield, reoperation, margin involvement and mesorectal quality are unchanged, the excluded cohort having contributed to none of them: its operative time was reported as a median with a range, which the protocol does not convert.

S6. Relationship to a previously registered review

A systematic review with a related question was registered in PROSPERO in February 2025 under CRD42025643154. The submitted version stated that no report of that review had appeared; that statement was wrong. The colorectal meta-analysis by Li and colleagues published in 2025 (reference 5 of the main text), which is discussed in the Introduction and Discussion, states that it is registered under CRD42025643154, so the registered review has been published. We did not link the published article to the registration number before submission, and our PROSPERO record, which justified proceeding on the ground that the earlier review had not been made available, contained the same error; the record has been amended. The present review was registered independently, after its database searches and title-and-abstract screening (Section S3). It differs from the published review in three respects: it is restricted to rectal cancer, excluding colonic and mixed resections without rectal-specific data; its unit of synthesis is the independent cohort rather than the publication; and it includes four cohorts that appear in neither previous synthesis.

S7. Deviations from the protocol, changes made in response to peer review, and corrections to the submitted version

Every change between the registered protocol, the submitted version and this revision is listed below, with its reason and its status. Post hoc denotes a decision taken after the results of the submitted version were known; each such analysis is reported alongside its pre-specified counterpart and neither is suppressed. Corrections are errors in the submitted version; none changed a conclusion. Reviewer points are numbered as in the response to reviewers.

A. Deviations from the registered protocol already present in the submitted version

No.	Item	Protocol or submitted version	This revision	Reason	Status
1	Population rule applied after data extraction	Protocol: mixed populations eligible only with rectal-specific data. One cohort with 39.3% rectosigmoid tumours was extracted and analysed	Cohort excluded; the deviation is now stated in the Methods of the main text and recorded in the PROSPERO record	Rule as registered; the excluded cohort was the most Xi-favourable (Section S5)	Deviation; conservative
2	Trial registers	Protocol listed a registry search	Not executed; stated in the Methods, the flow diagram and Section S3	Not executed	Deviation
3	Google Scholar stopping rule	Protocol: first 100 to 200 records of each strategy	First 100 of each strategy screened	Lower bound of the pre-specified range	Within protocol
4	Risk-of-bias tool	PROSPERO record: ROBINS-I	ROBINS-I V2 (20 November 2025 version)	Current version of the registered tool	Version update
5	Timing of registration	Submitted version: registered prospectively	Registered 18 August 2026, after the searches and title-and-abstract screening of 15–16 August and before data extraction	Reviewer 1, point 8	Reporting corrected

B. Changes to analyses and reporting made in response to peer review

No.	Item	Protocol or submitted version	This revision	Reason	Status
6	Operative time, primary analysis	Four cohorts reporting mean ± SD, 349 patients: MD −50.6 min (−69.4 to −31.7). The largest cohort, with converted medians, in a sensitivity analysis	Five cohorts, 553 patients, including the largest cohort with converted medians: MD −27.7 (−80.4 to +25.0). The four-cohort analysis is reported alongside and labelled conditional on excluding the largest cohort	The protocol rule excluded about two-fifths of the patients, and the only cohort in which the Xi was slower (Reviewer 1, point 1)	Post hoc; pre-specified analysis retained
7	Length of stay and lymph node yield, primary analyses	Cohorts reporting mean ± SD only; described as failed syntheses	Including the largest cohort with converted medians: MD −1.07 days (−3.67 to +1.52) and +2.29 nodes (−3.08 to +7.66); the pre-specified analyses, of three and four cohorts, retained	Same rule as item 6 (Reviewer 1, points 1 and 4)	Post hoc; pre-specified analyses retained
8	Operative time without the cohort whose endpoint may be console time	Not reported	−19.4 (−91.0 to +52.1) in the primary set and −41.4 (−70.8 to −12.0) in the conditional set	Timing endpoint of that cohort not defined (Reviewer 1, point 1)	Post hoc sensitivity analysis
9	Docking time	Pooled MD −4.04 min (−8.32 to +0.24);	Not pooled; study-level estimates −3.10, −6.00	One of three cohorts	Post hoc

No.	Item	Protocol or submitted version	This revision	Reason	Status
		methods stated a common scale	and −2.96 min, three of three favouring the Xi, sign test P = 0.25; pooled MD and SMD shown for completeness only	defines docking time and absolute times differ threefold (Reviewer 1, point 3)	
10	The term failed synthesis	Used for length of stay and lymph node yield without an objective criterion	Dropped; heterogeneity described with I^2, its confidence interval and the prediction interval	Reviewer 1, point 4	Reporting
11	Lymph node yield without the discordant cohort	Heterogeneity described as attributable entirely to one cohort	Labelled post hoc and exploratory, not used for inference; heterogeneity described as largely from that cohort	Reviewer 1, point 4	Post hoc, exploratory
12	I^2 reporting	Point estimate only; 0% read as consistency	Reported with its 95% confidence interval; 0% with three to five cohorts not read as consistency	Reviewer 1, point 4	Reporting
13	Alternative interval	Hartung–Knapp only	Hartung–Knapp with the ad hoc variance correction also reported, and used for interpretation where the two intervals differ in whether they include no effect	τ^2 estimated as zero for every dichotomous outcome (Reviewer 1, points 4 and 5)	Post hoc
14	Conversion to open surgery	RR 1.41 (0.19 to 10.29) on three informative cohorts; risk difference as sensitivity analysis	Risk difference primary: +0.07 percentage points (−0.70 to +0.83); RR, Peto OR and GLMM as sensitivity analyses	Five events; ratio estimates uninformative (Reviewer 1, point 5)	Post hoc; pre-specified analysis retained
15	Reoperation	RR 1.66 (0.25 to 11.22)	Risk difference primary: +2.6 percentage points (−5.3 to +10.6); RR, Peto OR and GLMM as sensitivity analyses	Sixteen events in three cohorts (Reviewer 1, point 5)	Post hoc; pre-specified analysis retained
16	Mesorectal specimen quality	Success-scale RR 0.99 (0.94 to 1.05) primary	Risk difference primary: −0.6 percentage points (−6.2 to +4.9); success-scale and failure-scale RR and GLMM as sensitivity analyses	Two cohorts with no incomplete specimen carried most of the success-scale weight (Reviewer 1, point 5)	Post hoc; pre-specified analysis retained
17	Sparse-data models	Not performed	Binomial-normal generalised linear mixed models for every dichotomous outcome	Reviewer 1, point 5	Post hoc sensitivity analyses
18	GRADE starting level and domain reporting	Certainty started at low; domain judgements in Online Resource 1 only	Certainty starts at high with ROBINS-I and is rated down two levels for serious and three for very serious risk of bias; every domain judgement for every outcome in Table 3; every outcome remains very low	Reviewer 1, point 9	Method corrected; no rating changed
19	Wording of findings and conclusions	The Xi may shorten operative and docking time	The evidence is very uncertain for every outcome; operative time was shorter with the Xi	Reviewer 1, point 2; editor	Reporting

No.	Item	Protocol or submitted version	This revision	Reason	Status
			only when the largest cohort was excluded		
20	Title	Perioperative, Oncological and Functional Outcomes	Perioperative and Oncological Outcomes; the absence of any between-platform functional comparison is reported as a finding	Reviewer 1, point 2	Reporting
21	Timing, centres and co-interventions	Stated that the Xi replaced the Si at the same institution in every cohort	Tabulated per cohort in Table 1 and Supplementary Table 7: four sequential within the same team, one by centre, one concurrent after an Si-only period, two not reported	Reviewer 1, point 7	Correction and new material
22	Outcome definitions	Not tabulated	Supplementary Table 6	Reviewer 1, point 3 and minor comment	New material
23	Limitations, implications and clinical relevance	Limitations within the Discussion	Separate Limitations section and Implications and future research section; paragraph on whom the comparison informs	Reviewer 1, point 10; Reviewer 2	Reporting
24	Figures and Table 1	Flow diagram without counts for other methods and with clipped text; asterisk in Fig. 2c undefined; axis capitalisation inconsistent in Fig. 3; three-group p values for one cohort in Table 1	Flow diagram redrawn with counts for other methods and a note on the linked letter; asterisk defined; sentence case throughout; the three-group p values removed and the summed Si arm identified	Reviewer 1, minor comments	Reporting

C. Corrections of errors in the submitted version

No.	Item	Protocol or submitted version	This revision	Reason	Status
25	Events in the margin analysis	16 events	14 events in the four informative cohorts of the primary analysis	Arithmetic error	Correction
26	Risks with the Si in the summary of findings	Conversion 23 per 1000 and margin involvement 36 per 1000, from informative cohorts only	7 and 28 per 1000, from all Si patients in every cohort in the primary analysis	Reviewer 1, point 6	Correction
27	Sign of the pooled docking difference	Results text: pooled mean difference 4.0 minutes (−8.3 to +0.2); Table 2 gave −4.04	−4.0; now shown for completeness only (item 9)	Typographical error	Correction
28	Leave-one-out range for anastomotic leak	0.55 to 0.67, from a superseded analysis	0.47 to 0.61	Not updated after the final re-analysis	Correction
29	Description of the dichotomous model	Mantel–Haenszel risk ratios without zero-cell correction	Inverse-variance random-effects pooling of study-level estimates with a 0.5 zero-cell correction; Mantel–Haenszel given in Online Resource 4 (Section S2)	Inaccurate description; no estimate changed	Correction
30	Contribution of the mixed colorectal cohort	Results stated that it contributed only margin involvement	Margin involvement, and operative time on its rectal procedures in one sensitivity analysis	Reviewer 1, point 6	Correction
31	Baseline stage differences	Stage differed in one cohort	Significantly in one cohort, and in two others on tests that are not directly comparable	Reviewer 1, point 6	Correction

No.	Item	Protocol or submitted version	This revision	Reason	Status
32	Operative-time sample of the largest cohort	Xi arm entered with 116 patients	80 patients, operative time not having been recorded at one Xi centre; 116 retained as a sensitivity analysis	Reviewer 1, point 1	Correction
33	Previously registered review (Section S6)	No report had appeared	Published in 2025 (reference 5 of the main text)	Registration number printed in that article	Correction
34	Supplementary Table 2, excluded comment	Cited in the Discussion	Not cited in the main text	The comment is not cited	Correction
35	Supplementary Table 3, major complications in one cohort	Labelled reported	Labelled author-supplied	Counts supplied in correspondence	Correction
36	Supplementary Table 3 and Supplementary Table 5, outcome labels	Overall complications and reoperation labelled 30 days or less	Labels removed; windows stated per cohort (Section S2, Supplementary Table 6)	Only one cohort states a 30-day window	Correction
37	Supplementary Table 3, one cohort's anastomotic-complication counts	Described as anastomotic complications rather than leak	The table-text conflict of labels stated; classification unchanged	Completeness	Clarification
38	Supplementary Table 4, panel B	Five rationales referred to internal project records, and one stated that author correspondence had been prepared	Internal references removed; the corresponding author's confirmation of the leak counts is recorded; judgement unchanged (serious)	Internal references are not available to readers; status out of date	Correction; no judgement changed
39	Supplementary Table 4, panel B, confounding rationale for two cohorts	Multicentre cohort described as period-separated; most recent cohort described as collinear with period throughout	Platform coincided with centre, with overlapping periods; platforms concurrent after an Si-only period; judgements unchanged (serious)	Reviewer 1, point 7	Correction; no judgement changed
40	Supplementary Table 4, panels A and B, multicentre cohort	Operative-efficiency result labelled sensitivity analysis only	Label removed; the result now enters the primary analysis (item 6)	Consequence of item 6	Consequential change
41	Supplementary Table 5	Certainty started at low; the Xi said to have replaced the Si sequentially in all eight cohorts; docking pooled	Rebuilt to match Table 3 and items 6 to 21	Reviewer 1, points 3, 5, 7 and 9	Correction

Supplementary Table 1. Full search strategies as executed. Each strategy is reproduced verbatim, exactly as run on the platform named; no strategy has been rewritten, standardised or reformatted for appearance. All strategies combine three concept blocks with AND — rectal cancer, robotic surgery and platform-specific terms — with terms combined by OR within each block. No date, language, publication-type or study-design limit was applied to any search.

Source	Platform, fields, filters, date and yield	Strategy as executed
PubMed/MEDLINE	Platform: pubmed.ncbi.nlm.nih.gov<br>Fields: MeSH terms plus Title/Abstract [tiab]<br>Filters: none<br>Date run: 16 August 2026<br>Records exported: 364	((("Rectal Neoplasms"[Mesh] OR "Rectum"[Mesh:NoExp] OR "Colorectal Neoplasms"[Mesh]<br>OR rectal cancer*[tiab] OR rectal carcinom*[tiab] OR rectal neoplas*[tiab]<br>OR rectal tumo*[tiab] OR rectal adenocarcinom*[tiab] OR rectum cancer*[tiab]<br>OR "cancer of the rectum"[tiab] OR rectosigmoid[tiab] OR colorectal cancer*[tiab]<br>OR colorectal neoplas*[tiab] OR "total mesorectal excision"[tiab] OR TME[tiab]<br>OR "low anterior resection"[tiab] OR "anterior resection"[tiab]<br>OR "abdominoperineal resection"[tiab] OR "intersphincteric resection"[tiab]<br>OR proctectom*[tiab] OR rectal resection*[tiab]))<br>AND<br>(("Robotic Surgical Procedures"[Mesh] OR "Robotics"[Mesh]<br>OR robotic*[tiab] OR robot-assisted[tiab] OR robot assisted[tiab]<br>OR robotically[tiab] OR "da Vinci"[tiab] OR daVinci[tiab]<br>OR "Intuitive Surgical"[tiab]))<br>AND<br>(("da Vinci Xi"[tiab] OR "daVinci Xi"[tiab] OR "Xi system"[tiab]<br>OR "Xi platform"[tiab] OR "Xi robot"[tiab] OR "Xi Surgical System"[tiab]<br>OR "da Vinci Si"[tiab] OR "daVinci Si"[tiab] OR "Si system"[tiab]<br>OR "Si platform"[tiab] OR "Si robot"[tiab] OR "Si Surgical System"[tiab]<br>OR "Xi vs Si"[tiab] OR "Si vs Xi"[tiab] OR "Xi versus Si"[tiab]<br>OR "Si versus Xi"[tiab] OR "robotic platform*"[tiab]<br>OR "robotic generation*"[tiab] OR "robotic system generation*"[tiab]<br>OR "fourth-generation"[tiab] OR "third-generation"[tiab]))
Embase	Platform: Embase.com (Elsevier)<br>Vocabulary: Emtree with explosion, plus title/abstract/keyword<br>Filters: none<br>Date run: 15 August 2026<br>Records exported: 836	(('rectum tumor'/exp OR 'rectum cancer'/exp OR 'colorectal tumor'/exp<br>OR 'rectum carcinoma'/exp OR 'total mesorectal excision'/exp<br>OR 'rectum resection'/exp<br>OR 'rectal cancer*':ti,ab,kw OR 'rectal carcinoma*':ti,ab,kw<br>OR 'rectal neoplas*':ti,ab,kw OR 'rectal tumo*':ti,ab,kw<br>OR 'rectal adenocarcinoma*':ti,ab,kw OR 'cancer of the rectum':ti,ab,kw<br>OR 'rectosigmoid':ti,ab,kw OR 'colorectal cancer*':ti,ab,kw<br>OR 'total mesorectal excision':ti,ab,kw OR 'low anterior resection':ti,ab,kw<br>OR 'abdominoperineal resection':ti,ab,kw<br>OR 'intersphincteric resection':ti,ab,kw OR 'proctectom*':ti,ab,kw)<br>AND<br>('robot assisted surgery'/exp OR 'robotics'/exp OR 'robot'/exp<br>OR 'da vinci robot'/exp<br>OR 'robotic*':ti,ab,kw OR 'robot assisted':ti,ab,kw<br>OR 'robot-assisted':ti,ab,kw OR 'da vinci':ti,ab,kw

Source	Platform, fields, filters, date and yield	Strategy as executed
		OR 'davinci':ti,ab,kw OR 'intuitive surgical':ti,ab,kw)<br>AND<br>('da vinci xi':ti,ab,kw OR 'davinci xi':ti,ab,kw OR 'xi system':ti,ab,kw<br>OR 'xi platform':ti,ab,kw OR 'xi robot':ti,ab,kw<br>OR 'da vinci si':ti,ab,kw OR 'davinci si':ti,ab,kw<br>OR 'si system':ti,ab,kw OR 'si platform':ti,ab,kw OR 'si robot':ti,ab,kw<br>OR ((xi OR si) NEAR/3 (robot* OR platform* OR system* OR generation*)):ti,ab,kw<br>OR 'robotic platform*':ti,ab,kw OR 'robotic generation*':ti,ab,kw))
Scopus	Platform: scopus.com Advanced Search<br>Field code: TITLE-ABS-KEY<br>Filters: none<br>Date run: 15 August 2026<br>Records exported: 596	TITLE-ABS-KEY (("rectal cancer*" OR "rectal carcinoma*" OR "rectal neoplas*"<br>OR "rectal tumo*" OR "rectal adenocarcinoma*" OR "cancer of the rectum"<br>OR "rectum cancer*" OR rectosigmoid OR "colorectal cancer*"<br>OR "colorectal neoplas*" OR "total mesorectal excision"<br>OR "low anterior resection" OR "abdominoperineal resection"<br>OR "intersphincteric resection" OR proctectom* OR "rectal resection*"))<br>AND TITLE-ABS-KEY ((robotic* OR "robot-assisted" OR "robot assisted"<br>OR robotically OR "da Vinci" OR daVinci OR "Intuitive Surgical"))<br>AND TITLE-ABS-KEY (("da Vinci Xi" OR "daVinci Xi" OR "Xi system"<br>OR "Xi platform" OR "Xi robot" OR "Xi Surgical System"<br>OR "da Vinci Si" OR "daVinci Si" OR "Si system" OR "Si platform"<br>OR "Si robot" OR "Si Surgical System"<br>OR ((Xi OR Si) W/3 (robot* OR platform* OR system* OR generation*))<br>OR "robotic platform*" OR "robotic generation*"))
Web of Science Core Collection	Platform: Clarivate Web of Science, Advanced Search<br>Field tag: TS (Topic)<br>Editions: SCI-EXPANDED, SSCI, ESCI, CPCI-S<br>Filters: none<br>Date run: 16 August 2026<br>Records exported: 362	TS=("rectal cancer*" OR "rectal carcinoma*" OR "rectal neoplas*"<br>OR "rectal tumo$r*" OR "rectal adenocarcinoma*" OR "cancer of the rectum"<br>OR "rectum cancer*" OR rectosigmoid OR "colorectal cancer*"<br>OR "colorectal neoplas*" OR "total mesorectal excision"<br>OR "low anterior resection" OR "abdominoperineal resection"<br>OR "intersphincteric resection" OR proctectom* OR "rectal resection*")<br>AND<br>TS=(robotic* OR "robot-assisted" OR "robot assisted" OR robotically<br>OR "da Vinci" OR daVinci OR "Intuitive Surgical")<br>AND<br>TS=("da Vinci Xi" OR "daVinci Xi" OR "Xi system" OR "Xi platform"<br>OR "Xi robot" OR "Xi Surgical System" OR "da Vinci Si" OR "daVinci Si"<br>OR "Si system" OR "Si platform" OR "Si robot" OR "Si Surgical System"<br>OR ((Xi OR Si) NEAR/3 (robot* OR platform* OR system* OR generation*))<br>OR "robotic platform*" OR "robotic generation*")
Cochrane CENTRAL	Platform: Cochrane Library (Wiley), Search Manager<br>Fields: MeSH descriptors plus title–	#1 MeSH descriptor: [Rectal Neoplasms] explode all trees 3152<br>#2 MeSH descriptor: [Colorectal Neoplasms] explode all trees 13919<br>#3 (rectal NEXT cancer*):ti,ab,kw

Source	Platform, fields, filters, date and yield	Strategy as executed
	abstract–keyword (ti,ab,kw)<br>Filters: none<br>Date run: 16 August 2026, 15:08:16<br>Records exported: 24	OR (rectal NEXT carcinoma*):ti,ab,kw<br>OR (rectal NEXT neoplas*):ti,ab,kw<br>OR (rectal NEXT tumo*):ti,ab,kw<br>OR (cancer NEXT of NEXT the NEXT rectum):ti,ab,kw<br>OR (rectosigmoid):ti,ab,kw<br>OR (colorectal NEXT cancer*):ti,ab,kw<br>OR (total NEXT mesorectal NEXT excision):ti,ab,kw<br>OR (low NEXT anterior NEXT resection):ti,ab,kw<br>OR (abdominoperineal NEXT resection):ti,ab,kw<br>OR (intersphincteric NEXT resection):ti,ab,kw<br>OR (proctectom*):ti,ab,kw 24073<br>#4 #1 OR #2 OR #3 27797<br>#5 MeSH descriptor: [Robotic Surgical Procedures] explode all trees 1217<br>#6 MeSH descriptor: [Robotics] explode all trees 2395<br>#7 (robotic*):ti,ab,kw<br>OR (robot NEXT assisted):ti,ab,kw<br>OR (robotically):ti,ab,kw<br>OR (da NEXT Vinci):ti,ab,kw<br>OR (daVinci):ti,ab,kw<br>OR (Intuitive NEXT Surgical):ti,ab,kw 9223<br>#8 #5 OR #6 OR #7 9223<br>#9 (da NEXT Vinci NEXT Xi):ti,ab,kw<br>OR (daVinci NEXT Xi):ti,ab,kw<br>OR (Xi NEXT system):ti,ab,kw<br>OR (Xi NEXT platform):ti,ab,kw<br>OR (Xi NEXT robot):ti,ab,kw<br>OR (da NEXT Vinci NEXT Si):ti,ab,kw<br>OR (daVinci NEXT Si):ti,ab,kw<br>OR (Si NEXT system):ti,ab,kw<br>OR (Si NEXT platform):ti,ab,kw<br>OR (Si NEXT robot):ti,ab,kw<br>OR (robotic NEXT platform*):ti,ab,kw<br>OR (robotic NEXT generation*):ti,ab,kw 249<br>#10 #4 AND #8 AND #9 24
Google Scholar (supplementary source)	Platform: scholar.google.com<br>Fields: free text; no controlled vocabulary and no reproducible total retrieval count<br>Dates run: 20–21 August 2026<br>Screening rule applied: the first 100	Search 1: "da Vinci Xi" "da Vinci Si" rectal cancer<br>Search 2: "da Vinci Xi" "da Vinci Si" "total mesorectal excision"<br>Search 3: robotic rectal cancer "Xi" "Si" comparison operative time outcomes<br>Search 4: "Xi versus Si" OR "Si versus Xi" robotic rectal resection<br>Search 5: robotic "rectal cancer" "robotic platform" generation comparison perioperative oncological functional outcomes

Source	Platform, fields, filters, date and yield	Strategy as executed
	records of each strategy, screened on title and abstract	

Total records imported 2,182; duplicates removed 1,000; unique records screened 1,182. The counts printed at the end of each Cochrane CENTRAL search line are the hits returned by that line, reproduced from the Search Manager export log. The Web of Science field tag TS searches the topic field. Google Scholar supports neither controlled vocabulary nor a reproducible total retrieval count and was used as a supplementary discovery source only; the screening rule stated for it was fixed in the registered protocol before the search was run. Trial registers were not searched (Section S3).

Supplementary Table 2. Reports assessed at full text and excluded, with reasons. The order follows the two identification routes of the PRISMA 2020 flow diagram and, within each route, the exclusion categories shown there.

No.	Reference	Identified via	Reason for exclusion
1	Protyniak B, Jorden J, Farmer R. Multiquadrant robotic colorectal surgery: the da Vinci Xi vs Si comparison. J Robot Surg 2018;12(1):67–74. https://doi.org/10.1007/s11701-017-0689-x	Databases	Mixed colorectal and benign population without separable rectal-specific data. Outcomes are reported for the whole colorectal series; no outcome carries a rectal-specific denominator.
2	Hill A, McCormick J. In experienced hands, does the robotic platform impact operative efficiency? Comparison of the da Vinci Si versus Xi robot in colorectal surgery. J Robot Surg 2020;14(5):789–792. https://doi.org/10.1007/s11701-020-01055-w	Databases	Mixed colorectal and benign population without separable rectal-specific data. The low anterior resection subgroup (Xi 25, Si 22) contains diverticulitis and inflammatory bowel disease as well as cancer and is not separable by diagnosis.
3	Gatto T, Monsellato I, Palucci M, et al. Robotic rectal cancer surgery in a medium-volume center: oncologic and functional outcomes of an 8-year experience. J Robot Surg 2025;19(1):749. https://doi.org/10.1007/s11701-025-02936-8	Databases	No outcome reported separately by platform. The da Vinci Si and Xi cases are analysed as one combined cohort.
4	Monsellato I, Gatto T, Lodin M, et al. Long-term single-surgeon outcomes of 320 robotic colorectal cancer resections: safety, oncologic reliability, and cost-effectiveness. Int J Med Robot 2025;21(5):e70126. https://doi.org/10.1002/rcs.70126	Databases	No outcome reported separately by platform; population overlaps the preceding report from the same centre.
5	Fujita Y, Ishimoto T, Kosuga T, Nakashima S, Fujiyama J, Masuyama M. A study of the invasiveness of robotic surgery with da Vinci Si or Xi for rectal cancer. Gan To Kagaku Ryoho 2024;51(13):1618–1620 (in Japanese).	Databases	Rectosigmoid tumours not separable from rectal tumours. Forty-eight of 122 patients (39.3%) are classified RS, tumour location is the only baseline variable differing significantly between the arms, and no outcome is reported with the rectosigmoid patients removed. See Section S5.
6	López-Huerta A, Reyes-Díaz ML, Bonilla-Cozar MA, et al. Evaluating short- and long-term clinical outcomes of robotic rectal cancer surgery: a comparison of da Vinci S and Xi systems. J Robot Surg 2025;19(1):720. https://doi.org/10.1007/s11701-025-02844-x	Other methods	Wrong comparator. The earlier-generation arm is the da Vinci S (IS2000), not the Si (IS3000); the Methods list the Si separately as a generation the centre did not use for this series.
7	Alshalawi W, Lee CS, et al. A comparative study on the short-term clinical outcomes of da Vinci SP versus da Vinci Xi for rectal cancer surgery. Int J Med Robot 2024;20(6):e2558. https://doi.org/10.1002/rcs.2558	Other methods	Wrong comparator. The comparison is the single-port da Vinci SP against the Xi.
8	Huang YL, Chen MC, Chiang FF. Robotic platform da Vinci Xi is feasible and beneficial in both colon and rectal surgery in short-term outcome and recovery. Gastroenterol Insights 2023;14(4):538–552. https://doi.org/10.3390/gastroent14040038	Other methods	Wrong comparator. The comparison is the Xi against conventional laparoscopy; only one robotic platform was used.
9	Li W, Chen ZH, Yang JS, et al. Comparison of perioperative outcomes and oncological efficacy between da Vinci Si and da Vinci Xi robotic systems in colorectal surgery: systematic review and meta-analysis of controlled trials. J Robot Surg 2025;19(1):332. https://doi.org/10.1007/s11701-025-02466-3	Other methods	Secondary evidence synthesis, not a primary study. Its reference list was screened and it is discussed in the main text. It is the published report of the review registered as CRD42025643154 (Section S6).

No.	Reference	Identified via	Reason for exclusion
10	Felsenreich DM, Gachabayov M, Karas J, Rojas A, Bergamaschi R; RoSiX Study Group. A meta-analysis of da Vinci Si versus Xi in colorectal surgery. Int J Med Robot 2021;17(3):e2222. https://doi.org/10.1002/rcs.2222	Other methods	Secondary evidence synthesis, not a primary study. Its reference list was screened and it is discussed in the main text.
11	Tseng YK, Chiang FF, Chen MC, Lin CY. Learning curve of da Vinci Xi robotic low anterior resection: a cumulative sum analysis of a single high-volume surgeon. J Clin Med 2026;15(3):1248. https://doi.org/10.3390/jcm15031248	Other methods	No da Vinci Xi versus Si comparison. The analysis is confined to Xi cases; Si cases are explicitly excluded.
12	Panteleimonitis S, Harper M, Hall S, Figueiredo N, Qureshi T, Parvaiz A. Precision in robotic rectal surgery using the da Vinci Xi system and integrated table motion, a technical note. J Robot Surg 2018;12(3):433–436. https://doi.org/10.1007/s11701-017-0752-7	Other methods	No da Vinci Xi versus Si comparison. Both arms are Xi, with and without integrated table motion.
13	Bianchi G, Gavriilidis P, Martínez-Pérez A, et al. Robotic multiquadrant colorectal procedures: a single-center experience and a systematic review of the literature. Front Surg 2022;9:991704. https://doi.org/10.3389/fsurg.2022.991704	Other methods	No platform-stratified outcome, and the population is predominantly inflammatory bowel disease.
14	Zhan R, Jiao C, Zhao Y, Han J. Comment on: comparison of perioperative outcomes and oncological efficacy between da Vinci Si and da Vinci Xi robotic systems in colorectal surgery: systematic review and meta-analysis of controlled trials. J Robot Surg 2025;19(1):431. https://doi.org/10.1007/s11701-025-02607-8	Other methods	Correspondence, not primary research. No original data. Not cited in the main text.

Five reports were excluded at full text among those identified through databases and registers, and nine among those identified through other methods; a tenth report assessed through other methods proved to be an additional report of an already-included cohort and was linked to it rather than excluded. No report sought for retrieval could not be obtained.

Supplementary Table 3. Complete outcome-level extraction. Every value entering every analysis in this review is listed. The da Vinci Xi is the experimental group and the da Vinci Si the control group throughout. Changes made in this revision are listed in Section S7.

Outcome and definition	Cohort	da Vinci Xi	da Vinci Si	Value	Basis, and any residual ambiguity
**Continuous outcomes — value shown as mean ± standard deviation (n)**					
Operative time, min<br>Total operating time as defined by each source	Ozben (2016)	321.6 ± 69.4 (28)	360.4 ± 63.2 (25)	Reported	
	Morelli (2018)	285 ± 49 (30)	318 ± 57 (30)	Reported	
	Aliyev (2022)	123.6 ± 25.7 (62)	181.3 ± 31.8 (88)	Reported	Timing endpoint not determinable from the source: Table 2 heads the variable operative time and Table 3 heads the same analysis console time; the Methods define neither. Basis of the indirectness downgrade for this outcome.
	Wang (2026)	226.71 ± 57.23 (55)	282.13 ± 80.76 (31)	Reported	
	Huang (2019)	241.8 ± 60.7 (28)	264.8 ± 72.1 (73)	Reported	Sensitivity analysis only; mixed colorectal cohort, rectal subgroup identified after surgery
	Panteleimonitis (2020)	289.45 ± 67.60 (80)	245.47 ± 72.08 (124)	Derived	Primary analysis in this revision; a sensitivity analysis in the submitted version, which entered the Xi arm with 116 patients. Operative time was not recorded at the Portuguese centre, all 36 of whose patients were Xi, so 80 Xi patients contribute. Source reports medians with interquartile ranges (Xi 300, IQR 240–330; Si 230, IQR 204–300). Mean by Luo 2018, standard deviation by Wan 2014, from the interquartile range only
Docking time, min<br>Time to dock the robotic arms	Ozben (2016)	5.3 ± 1.3 (28)	8.4 ± 3.6 (25)	Reported	
	Morelli (2018)	17.5 ± 3.4 (30)	23.5 ± 2.7 (30)	Reported	
	Wang (2026)	8.78 ± 3.33 (55)	11.74 ± 4.46 (31)	Reported	
Length of hospital stay, days	Ozben (2016)	6.2 ± 2.5 (28)	5.1 ± 3.3 (25)	Reported	
	Aliyev (2022)	5.6 ± 2.8 (62)	6.2 ± 3.4 (88)	Reported	

Outcome and definition	Cohort	da Vinci Xi	da Vinci Si	Value	Basis, and any residual ambiguity
	Wang (2026)	7.78 ± 1.69 (55)	9.68 ± 3.62 (31)	Reported	
	Panteleimonitis (2020)	5.28 ± 3.71 (116)	7.71 ± 5.21 (124)	Derived	Primary analysis in this revision; a sensitivity analysis in the submitted version. Source reports medians with interquartile ranges (Xi 5, IQR 3–8; Si 7, IQR 5–12). Converted as above
Lymph node yield, n<br>Nodes harvested in the resection specimen	Ozben (2016)	27.5 ± 14.0 (28)	17.0 ± 9.1 (25)	Reported	
	Morelli (2018)	16.6 ± 8.0 (30)	19.0 ± 8.0 (30)	Reported	
	Aliyev (2022)	26.1 ± 10.1 (62)	25.7 ± 10.6 (88)	Reported	
	Wang (2026)	16.84 ± 4.89 (55)	15.35 ± 8.09 (31)	Reported	
	Panteleimonitis (2020)	21.28 ± 8.15 (116)	17.53 ± 7.36 (124)	Derived	Primary analysis in this revision; a sensitivity analysis in the submitted version. Source reports medians with interquartile ranges (Xi 21, IQR 16–27; Si 17, IQR 13–23). Converted as above
**Dichotomous outcomes — value shown as events / denominator**					
Overall postoperative complications<br>Four definitions are in use across the five contributing cohorts, differing in whether medical as well as surgical events are counted; only one cohort states an ascertainment window, of 30 days. Each cohort's own definition was used (Supplementary Table 6)	Ozben (2016)	4 / 28	3 / 25	Reported	
	Morelli (2018)	6 / 30	8 / 30	Reported	
	Tung (2018)	5 / 24	5 / 33	Derived	Si arm is the sum of the two Si subgroups reported separately by the source (hybrid n = 19, totally robotic n = 14); the combined denominator of 33 is stated in the Results

Outcome and definition	Cohort	da Vinci Xi	da Vinci Si	Value	Basis, and any residual ambiguity
	Aliyev (2022)	7 / 62	9 / 88	Reported	
	Wang (2026)	4 / 55	5 / 31	Reported	
Anastomotic leak<br>Denominator restricted to patients who received an anastomosis; abdominoperineal resection, Hartmann's procedure and panproctocolectomy removed from both arms	Ozben (2016)	1 / 27	2 / 21	Derived	Denominator from the printed procedure table; determined exactly
	Morelli (2018)	2 / 28	3 / 28	Derived	Denominator from the printed procedure table; determined exactly. Counts confirmed by the corresponding author. Screen-detected radiological leak; basis of the indirectness downgrade for this outcome
	Tung (2018)	2 / 24	5 / 33	Derived	Low anterior resection with covering ileostomy in every patient, so the denominators equal the arm sizes. Si counts are the sum of the two reported Si subgroups
	Panteleimonitis (2020)	0 / 99	4 / 109	Derived	Arm sizes less abdominoperineal excision (17 and 12), Hartmann's procedure (0 and 2) and panproctocolectomy (0 and 1), from Table 3. See Section S2
	Wang (2026)	2 / 52	1 / 28	Derived	Arm sizes less Miles procedure (3 and 3), from Table 1. See Section S2
	Aliyev (2022)	7 / 62	5 / 88	Reported	Table 2 heads these counts anastomotic complications, and the Results text calls the same counts anastomotic leaks; neither is defined. Enters the wider-definition sensitivity analysis only
Conversion to open surgery	Ozben (2016)	1 / 28	1 / 25	Reported	
	Morelli (2018)	1 / 30	1 / 30	Reported	
	Tung (2018)	1 / 24	0 / 33	Derived	Si count is the sum of the two reported Si subgroups
	Panteleimonitis (2020)	0 / 116	0 / 124	Reported	No event in either arm; retained in the primary risk-difference analysis; contributes no

Outcome and definition	Cohort	da Vinci Xi	da Vinci Si	Value	Basis, and any residual ambiguity
					information to ratio estimates
	Aliyev (2022)	0 / 62	0 / 88	Reported	No event in either arm; retained in the primary risk-difference analysis; contributes no information to ratio estimates
Reoperation<br>Window 30 days in one cohort, within the 30-day outcome set in one, not stated in one	Ozben (2016)	1 / 28	2 / 25	Reported	
	Morelli (2018)	1 / 30	0 / 30	Reported	
	Panteleimonitis (2020)	8 / 116	4 / 124	Reported	
Circumferential resection margin involved<br>Tumour ≤ 1 mm from the circumferential resection margin	Ozben (2016)	3 / 28	0 / 25	Reported	
	Morelli (2018)	0 / 30	0 / 30	Reported	No event in either arm; contributes no information to the risk ratio
	Tung (2018)	0 / 24	0 / 33	Derived	No event in either arm; contributes no information to the risk ratio. Si denominator is the sum of the two reported Si subgroups
	Huang (2019)	2 / 29	6 / 77	Reported	Rectal subgroup; the only outcome in this source with a rectal-specific denominator
	Aliyev (2022)	0 / 62	1 / 88	Reported	
	Wang (2026)	1 / 55	1 / 31	Reported	
	Cha (2018)	1 / 36	1 / 55	Derived	Critical risk of bias; sensitivity analysis only. Counts derived from the printed margin-negativity rates of 97.2% of 36 and 98.2% of 55, which resolve uniquely to 35 and 54 negative specimens
Mesorectal specimen quality complete or near-complete<br>Quirke grading	Ozben (2016)	23 / 23	19 / 19	Reported	Graded only in patients who underwent total mesorectal excision, so the denominator is smaller than the arm size
	Morelli (2018)	30 / 30	30 / 30	Reported	

Outcome and definition	Cohort	da Vinci Xi	da Vinci Si	Value	Basis, and any residual ambiguity
	Aliyev (2022)	58 / 62	86 / 88	Reported	
	Wang (2026)	53 / 55	28 / 31	Reported	
	Cha (2018)	34 / 36	49 / 55	Derived	Critical risk of bias; sensitivity analysis only. Counts derived from the printed rates of 94.6% of 36 and 89.0% of 55, which resolve uniquely to 34 and 49
**Outcomes reported but not pooled (fewer than the three cohorts fixed in the protocol)**					
Major complications, Clavien–Dindo grade III or above	Morelli (2018)	1 / 30	2 / 30	Author-supplied	Counts supplied by the corresponding author; not printed in the publication
	Aliyev (2022)	2 / 62	3 / 88	Reported	The source reports no Clavien–Dindo category above grade III, so this count equals grade III or above only if no grade IV or V event occurred and went unreported
Diverting stoma	Morelli (2018)	28 / 30	28 / 30	Derived	Methods state that a diverting ileostomy was performed in every anterior and intersphincteric resection; Table 1 gives 22 anterior and 6 intersphincteric resections per arm
	Aliyev (2022)	58 / 62	81 / 88	Reported	
	Ozben (2016)	20 / 28	7 / 25	Reported	Critical risk of bias (confounding by indication: very low anterior resection 32.1% versus 4.0%, p = 0.01); excluded from the primary presentation
30-day mortality	Ozben (2016)	0 / 28	1 / 25	Reported	
	Morelli (2018)	0 / 30	0 / 30	Reported	
	Tung (2018)	0 / 24	0 / 33	Derived	Si denominator is the sum of the two reported Si subgroups
	Panteleimonitis (2020)	0 / 116	0 / 124	Reported	

Reported denotes a value printed in the source. Author-supplied denotes a value provided by the corresponding author in correspondence and not printed in the source. Derived denotes a value obtained by exact arithmetic from figures printed in the source; the derivation is given in the final column in every case. No value was imputed, no variance was borrowed between studies, and no median reported with a range was converted; medians reported with interquartile ranges were converted as stated.

Continuous values are the mean and standard deviation with the number of patients in parentheses. Dichotomous values are the number of events over the denominator at risk. Where a cohort is marked as contributing to a sensitivity analysis only, it does not enter the primary estimate of that outcome.

Supplementary Table 4, panel A. ROBINS-I V2 domain-level and overall judgements for all 23 assessed study results. Assessment was result-specific: one index numerical result was named for each study and outcome group, and results within a group were assessed separately where their domains differed.

Study	Result assessed	Index numerical result and its location in the source	D1 Confounding	D2 Classification of interventions	D3 Selection of participants	D4 Missing data	D5 Measurement of outcome	D6 Selection of the reported result	Overall
Ozben (2016)	G1 Operative efficiency	Console time 265.7 ± 60.9 vs 317.1 ± 70.5 min (Table 3, p. 420); multivariable OR 0.09, 95% CI 0.02-0.47 (Table 5, p. 421)	Serious	Low	Moderate	Moderate	Moderate	Moderate	Serious
Ozben (2016)	G2 Perioperative safety	Length of hospital stay 6.2 ± 2.5 vs 5.1 ± 3.3 days, p = 0.001 (Table 8, p. 422)	Serious	Low	Moderate	Moderate	Moderate	Moderate	Serious
Ozben (2016)	G3 Pathological / oncological	Radial (circumferential) margin involved 3/28 (10.7%) vs 0/25 (0%), p = 0.24 (Table 4, p. 421)	Serious	Low	Serious	Moderate	Low	Moderate	Serious
Ozben (2016)	G4 Process measure (diverting stoma)	Loop ileostomy 20/28 (71.4%) vs 7/25 (28.0%); stoma creation overall p = 0.005 (Table 2, p. 420)	Critical	Low	Moderate	Moderate	Low	Moderate	Critical
Cha (2018)	G3 Pathological / oncological	Mesorectum complete or near-complete 94.6% vs 89.0%, p = 0.1738; CRM negative 97.2% vs 98.2%, p = 0.4224 (Dis Colon Rectum 2018;61(5):e262)	Critical	Not assessed	Not assessed	Not assessed	Not assessed	Not assessed	Critical
Morelli (2018)	G1 Operative efficiency	Docking time 17.5 ± 3.4 vs 23.5 ± 2.7 min, p < 0.001 (Table 2, p. 592)	Serious	Low	Low	Moderate	Moderate	Moderate	Serious
Morelli (2018)	G2 Perioperative safety	Anastomotic radiological leak 2/30 (6.7%) vs 3/30 (10%), p = 0.64 (Table 4, p. 593)	Serious	Low	Low	Low	Moderate	Serious	Serious
Morelli (2018)	G3 Pathological / oncological	Lymph nodes harvested 16.6 ± 8.0 vs 19 ± 8, p = 0.31 (Table 3, p. 592)	Serious	Low	Low	Low	Low	Moderate	Serious
Morelli (2018)	G4 Process measure (diverting stoma)	Diverting ileostomy in all anterior and intersphincteric resections: 28/30 in each arm (Methods, p. 591; procedure counts Table 1, p. 592)	Moderate	Low	Low	Moderate	Low	Moderate	Serious
Tung (2018)	G2 Perioperative safety	Clinical anastomotic leakage 2/24 vs 5/33 (Table 2, Clinics in Surgery 2018;3:2014)	Serious	Low	Moderate	Moderate	Moderate	Serious	Serious
Tung (2018)	G3 Pathological / oncological	CRM involvement 0/24 vs 0/33 (Table 2)	Serious	Low	Moderate	Moderate	Low	Serious	Serious
Huang (2019)	G1 Operative efficiency (sensitivity analysis	Operative time for LAR + APR + Hartmann: 241.8 ± 60.7 (n = 28) vs 264.8 ± 72.1 (n = 73) min, p = 0.138 (Table 2, p. 196)	Serious	Low	Serious	Moderate	Moderate	Moderate	Serious

Study	Result assessed	Index numerical result and its location in the source	D1 Confounding	D2 Classification of interventions	D3 Selection of participants	D4 Missing data	D5 Measurement of outcome	D6 Selection of the reported result	Overall
	only)								
Huang (2019)	G3 Pathological / oncological	CRM involvement in the rectal subgroup 2/29 (6.9%) vs 6/77 (7.8%), p = 1 (Table 3, p. 196)	Serious	Low	Moderate	Moderate	Low	Moderate	Serious
Panteleimonitis (2020)	G1 Operative efficiency	Operative time median 300 (IQR 240-330) vs 230 (IQR 204-300) min, p < 0.001 (Table 4)	Serious	Low	Moderate	Moderate	Moderate	Moderate	Serious
Panteleimonitis (2020)	G2 Perioperative safety	Length of hospital stay median 5 (IQR 3-8) vs 7 (IQR 5-12) days, p = 0.010 (Table 5)	Serious	Low	Moderate	Moderate	Moderate	Moderate	Serious
Panteleimonitis (2020)	G3 Pathological / oncological	Lymph node yield median 21 (IQR 16-27) vs 17 (IQR 13-23), p = 0.010 (Table 5)	Serious	Low	Moderate	Moderate	Low	Moderate	Serious
Aliyev (2022)	G1 Operative efficiency	Operative time 123.6 ± 25.7 vs 181.3 ± 31.8 min, p < 0.001 (Table 2, p. 1342)	Serious	Low	Moderate	Moderate	Moderate	Serious	Serious
Aliyev (2022)	G2 Perioperative safety	Anastomotic complications 7/62 (11.3%) vs 5/88 (5.7%), p = 0.173 (Table 2, p. 1342)	Serious	Low	Moderate	Moderate	Moderate	Serious	Serious
Aliyev (2022)	G3 Pathological / oncological	Mesorectal excision complete or near-complete 58/62 (93.5%) vs 86/88 (97.7%), p = 0.504 (Table 2, p. 1342)	Serious	Low	Moderate	Moderate	Low	Serious	Serious
Aliyev (2022)	G4 Process measure (diverting stoma)	Diverting ileostomy 58/62 (93.5%) vs 81/88 (92.0%), p = 0.495 (Table 2, p. 1342)	Serious	Low	Moderate	Moderate	Low	Serious	Serious
Wang (2026)	G1 Operative efficiency	Operative time 226.71 ± 57.23 vs 282.13 ± 80.76 min, p = 0.010 (Table 2); multivariable linear regression (Table 4)	Serious	Low	Moderate	Moderate	Moderate	Moderate	Serious
Wang (2026)	G2 Perioperative safety	Postoperative complications 4/55 (7.3%) vs 5/31 (16.1%), p = 0.198 (Table 6)	Serious	Low	Moderate	Moderate	Moderate	Moderate	Serious
Wang (2026)	G3 Pathological / oncological	Mesorectal integrity grade 3 (complete) 53/55 (96.4%) vs 28/31 (90.3%), p = 0.608 (Table 7)	Serious	Low	Moderate	Moderate	Low	Moderate	Serious

The target trial against which every result is assessed is one in which patients would be randomly assigned to the da Vinci Xi or the da Vinci Si by the same surgical team in the same calendar period. Not assessed denotes a result rated at critical risk of bias at the ROBINS-I V2 screening step, for which the tool directs that no further domain assessment is required. Pre-specified confounding domains were calendar period, surgeon and team experience, institution, tumour height and stage, neoadjuvant therapy, procedure type, body mass index, sex and perioperative care protocol. Missing information was not interpreted as low risk of bias.

Supplementary Table 4, panel B. Supporting evidence and rationale for each domain judgement.

Study	Result assessed	Domain	Judgement	Supporting evidence and rationale
Ozben (2016)	G1 Operative efficiency	D1 Confounding	Serious	Adjusted for operative procedure, (y)pT class and mesorectal excision type, but not for calendar period or surgeon experience. The Si was used January 2010 to October 2014 and the Xi from November 2014 (Methods, p. 417), so platform is collinear with period and no adjustment is possible.
Ozben (2016)	G1 Operative efficiency	D2 Classification of interventions	Low	Platform is recorded at the time of operation from the theatre record; classification is unambiguous and cannot be influenced by the outcome.
Ozben (2016)	G1 Operative efficiency	D3 Selection of participants	Moderate	Consecutive series of all robotic rectal cancer resections in the period; follow-up begins at surgery. No post-intervention selection for this result.
Ozben (2016)	G1 Operative efficiency	D4 Missing data	Moderate	Completeness of the operative-time data is not reported. Absence of reporting is not treated as evidence of completeness.
Ozben (2016)	G1 Operative efficiency	D5 Measurement of outcome	Moderate	Times were recorded unblinded by the operating team, but are objective clock measurements obtained by the same method in both arms.
Ozben (2016)	G1 Operative efficiency	D6 Selection of reported result	Moderate	No pre-specified analysis plan or protocol is available for this retrospective series.
Ozben (2016)	G2 Perioperative safety	D1 Confounding	Serious	As above: period and experience uncontrolled. In addition the arms differ significantly in procedure type and stoma creation (Table 2), both of which affect length of stay, and this result is unadjusted.
Ozben (2016)	G2 Perioperative safety	D2 Classification of interventions	Low	As above.
Ozben (2016)	G2 Perioperative safety	D3 Selection of participants	Moderate	Consecutive series; 30-day outcomes reported for all patients.
Ozben (2016)	G2 Perioperative safety	D4 Missing data	Moderate	Readmission and reoperation reported for all; completeness not explicitly stated.
Ozben (2016)	G2 Perioperative safety	D5 Measurement of outcome	Moderate	Clinically ascertained, unblinded, but objective endpoints applied identically to both arms.
Ozben (2016)	G2 Perioperative safety	D6 Selection of reported result	Moderate	No pre-specified plan. The published abstract contains a typographical error ("significant differences ... P > 0.05") resolved against Table 2; the tables themselves are internally consistent.
Ozben (2016)	G3 Pathological / oncological	D1 Confounding	Serious	Period and experience uncontrolled; arms differ in stage ((y)pTNM III 39.3% vs 8.0%, p = 0.02) and in tumour distance from the anal verge (9.5 vs 12.0 cm), both of which affect margin risk. Unadjusted.
Ozben (2016)	G3 Pathological / oncological	D2 Classification of interventions	Low	As above.
Ozben (2016)	G3 Pathological / oncological	D3 Selection of participants	Serious	SERIOUS for the mesorectal-quality result within this group: quality of the mesorectal fascia was assessed only in patients who underwent total mesorectal excision (23 of 28 and 19 of 25, footnote to Table 4), so participants enter the analysis on the basis of the procedure actually performed, a post-intervention

Study	Result assessed	Domain	Judgement	Supporting evidence and rationale
				variable.
Ozben (2016)	G3 Pathological / oncological	D4 Missing data	Moderate	Pathology reported for all patients in the CRM analysis.
Ozben (2016)	G3 Pathological / oncological	D5 Measurement of outcome	Low	Assessed by a pathologist working from the resected specimen against a standard threshold (tumour within 1 mm), with no plausible influence of knowledge of the platform.
Ozben (2016)	G3 Pathological / oncological	D6 Selection of reported result	Moderate	No pre-specified plan.
Ozben (2016)	G4 Process measure (diverting stoma)	D1 Confounding	Critical	CRITICAL. The decision to defunction is determined by tumour height and procedure type, and the two arms differ significantly in exactly those variables: very low anterior resection 9/28 (32.1%) versus 1/25 (4.0%), p = 0.01, and abdominoperineal resection 1 (3.6%) versus 4 (16.0%) (Table 2). The result is unadjusted. It therefore cannot be interpreted as an effect of the robotic platform, and under ROBINS-I V2 a result at critical risk should generally be excluded from evidence syntheses.
Ozben (2016)	G4 Process measure (diverting stoma)	D2 Classification of interventions	Low	As above.
Ozben (2016)	G4 Process measure (diverting stoma)	D3 Selection of participants	Moderate	Consecutive series.
Ozben (2016)	G4 Process measure (diverting stoma)	D4 Missing data	Moderate	Stoma status recorded for all patients.
Ozben (2016)	G4 Process measure (diverting stoma)	D5 Measurement of outcome	Low	Objective, taken from the operative record.
Ozben (2016)	G4 Process measure (diverting stoma)	D6 Selection of reported result	Moderate	No pre-specified plan.
Cha (2018)	G3 Pathological / oncological	D1 Confounding	Critical	CRITICAL by screening question B2. The publication is a conference abstract reporting no adjustment, no matching and no baseline characteristics. The Xi became commercially available in April 2014, so within a 2011-2017 series the platforms are necessarily period-separated. Under ROBINS-I V2, if B2 is answered Yes the result is at critical risk of bias and no further assessment is required.
Cha (2018)	G3 Pathological / oncological	D2 Classification of interventions	Not assessed	Not assessed - screening step B terminates the assessment.
Cha (2018)	G3 Pathological / oncological	D3 Selection of participants	Not assessed	Not assessed.
Cha (2018)	G3 Pathological / oncological	D4 Missing data	Not assessed	Not assessed.
Cha (2018)	G3 Pathological / oncological	D5 Measurement of outcome	Not assessed	Not assessed.

Study	Result assessed	Domain	Judgement	Supporting evidence and rationale
Cha (2018)	G3 Pathological / oncological	D6 Selection of reported result	Not assessed	Not assessed.
Morelli (2018)	G1 Operative efficiency	D1 Confounding	Serious	1:1 case-matching on eight patient and tumour variables is the strongest confounding control in the review, but it does not and cannot address calendar period or surgeon experience: the Si was used April 2010 to December 2014 and the Xi from January 2015 (Methods, p. 590), by a single surgeon whose robotic experience increased across that interval. The authors acknowledge a possible proficiency-gain effect (Discussion, p. 598).
Morelli (2018)	G1 Operative efficiency	D2 Classification of interventions	Low	Platform recorded at operation.
Morelli (2018)	G1 Operative efficiency	D3 Selection of participants	Low	The first 30 consecutive Xi cases were matched to Si controls from the same prospective database; follow-up begins at surgery.
Morelli (2018)	G1 Operative efficiency	D4 Missing data	Moderate	Completeness of operative data not explicitly reported.
Morelli (2018)	G1 Operative efficiency	D5 Measurement of outcome	Moderate	Unblinded but objective; the same timing convention applied to both arms.
Morelli (2018)	G1 Operative efficiency	D6 Selection of reported result	Moderate	No pre-specified plan.
Morelli (2018)	G2 Perioperative safety	D1 Confounding	Serious	As above.
Morelli (2018)	G2 Perioperative safety	D2 Classification of interventions	Low	As above.
Morelli (2018)	G2 Perioperative safety	D3 Selection of participants	Low	As above.
Morelli (2018)	G2 Perioperative safety	D4 Missing data	Low	All patients were available at every follow-up time point (Methods, p. 590), which is explicitly stated - one of the few studies to do so.
Morelli (2018)	G2 Perioperative safety	D5 Measurement of outcome	Moderate	Leak was ascertained by routine water-soluble contrast enema one month after surgery in every patient (Methods, p. 590). This is a screening-detected outcome that will capture subclinical leaks, but it was applied identically to both arms, so it is comparable within the study. It is not comparable with the clinically diagnosed leak reported by other cohorts, which is a between-study indirectness issue rather than within-study bias.
Morelli (2018)	G2 Perioperative safety	D6 Selection of reported result	Serious	SERIOUS. The anastomotic leak and complication counts used are those printed in Table 4; the corresponding author confirmed the leak counts in correspondence (Supplementary Table 3). Judgement unchanged.
Morelli (2018)	G3 Pathological / oncological	D1 Confounding	Serious	As above.
Morelli (2018)	G3 Pathological /	D2 Classification of	Low	As above.

Study	Result assessed	Domain	Judgement	Supporting evidence and rationale
	oncological	interventions		
Morelli (2018)	G3 Pathological / oncological	D3 Selection of participants	Low	As above.
Morelli (2018)	G3 Pathological / oncological	D4 Missing data	Low	As above.
Morelli (2018)	G3 Pathological / oncological	D5 Measurement of outcome	Low	Pathological assessment of the specimen; mesorectal quality graded by Quirke criteria and CRM at a 1 mm threshold. No plausible influence of platform knowledge.
Morelli (2018)	G3 Pathological / oncological	D6 Selection of reported result	Moderate	No pre-specified plan. The pathological results are internally consistent between table and text, unlike the safety results.
Morelli (2018)	G4 Process measure (diverting stoma)	D1 Confounding	Moderate	MODERATE rather than serious for this specific result: the arms were matched on type of operation, so the principal determinant of stoma creation is balanced by design. Period and experience remain uncontrolled but have little bearing on a policy-driven decision.
Morelli (2018)	G4 Process measure (diverting stoma)	D2 Classification of interventions	Low	As above.
Morelli (2018)	G4 Process measure (diverting stoma)	D3 Selection of participants	Low	As above.
Morelli (2018)	G4 Process measure (diverting stoma)	D4 Missing data	Moderate	The count is derived from the statement that all ARR and ISR patients received a diverting ileostomy combined with the procedure counts, rather than printed directly.
Morelli (2018)	G4 Process measure (diverting stoma)	D5 Measurement of outcome	Low	Objective, from the operative record.
Morelli (2018)	G4 Process measure (diverting stoma)	D6 Selection of reported result	Moderate	No pre-specified plan.
Tung (2018)	G2 Perioperative safety	D1 Confounding	Serious	No adjustment or matching. Baseline reporting is limited to age, sex and neoadjuvant treatment (Table 1); tumour height, stage, BMI and ASA are not reported, so the comparability of the arms in the principal prognostic variables cannot be judged. Period separation within January 2016 to April 2018 is not described.
Tung (2018)	G2 Perioperative safety	D2 Classification of interventions	Low	Platform and approach recorded at operation; the three arms are clearly defined.
Tung (2018)	G2 Perioperative safety	D3 Selection of participants	Moderate	Consecutive series of robotic rectal cancer resections; the two Si arms were combined to n = 33 for this review, which does not alter selection.
Tung (2018)	G2 Perioperative safety	D4 Missing data	Moderate	Completeness not reported.
Tung (2018)	G2 Perioperative safety	D5 Measurement of outcome	Moderate	Clinically ascertained, unblinded, objective endpoints.
Tung (2018)	G2 Perioperative safety	D6 Selection of reported result	Serious	SERIOUS. Several reported p values are arithmetically impossible (blood loss 55 / 59 / 60 mL with p = 0.026; conversion 0 / 0 / 1 with p = 0; 30-day mortality 0 / 0 / 0

Study	Result assessed	Domain	Judgement	Supporting evidence and rationale
				with p = 0), the abstract contradicts itself on whether operative time was significant, and Table 1 reports neoadjuvant treatment as p = 0.046 while the text states the difference was not significant. The reliability of any reported figure is therefore in doubt. The event counts extracted for this review are internally consistent with the stated denominators, which is why this domain is rated serious rather than critical.
Tung (2018)	G3 Pathological / oncological	D1 Confounding	Serious	As above.
Tung (2018)	G3 Pathological / oncological	D2 Classification of interventions	Low	As above.
Tung (2018)	G3 Pathological / oncological	D3 Selection of participants	Moderate	As above.
Tung (2018)	G3 Pathological / oncological	D4 Missing data	Moderate	Completeness not reported.
Tung (2018)	G3 Pathological / oncological	D5 Measurement of outcome	Low	Pathological assessment from the specimen.
Tung (2018)	G3 Pathological / oncological	D6 Selection of reported result	Serious	As above. This result contributes no information to the meta-analysis (zero events in both arms).
Huang (2019)	G1 Operative efficiency (sensitivity analysis only)	D1 Confounding	Serious	No adjustment. The Si group was operated December 2011 to May 2015 and the Xi group May 2015 to October 2017, with no overlap. Baseline imbalance is documented: clinically node-positive disease in 75.8% versus 50%, p = 0.002 (Table 1). The authors acknowledge that sequential accrual may bias outcomes in favour of the Xi group (Discussion, p. 198).
Huang (2019)	G1 Operative efficiency (sensitivity analysis only)	D2 Classification of interventions	Low	Platform recorded at operation.
Huang (2019)	G1 Operative efficiency (sensitivity analysis only)	D3 Selection of participants	Serious	SERIOUS. This result is restricted to a subgroup defined by the procedure actually performed (left hemicolectomy, anterior resection, low anterior resection, APR and Hartmann), which is a post-intervention variable, and is drawn from a mixed colon-and-rectum cohort. Follow-up begins at surgery, but selection into this particular analysis occurs after the intervention.
Huang (2019)	G1 Operative efficiency (sensitivity analysis only)	D4 Missing data	Moderate	Completeness not reported.
Huang (2019)	G1 Operative efficiency (sensitivity analysis only)	D5 Measurement of outcome	Moderate	Operative time defined as skin incision to completion of wound closure (Methods, p. 194), applied identically to both arms; unblinded.
Huang (2019)	G1 Operative efficiency (sensitivity analysis only)	D6 Selection of reported result	Moderate	No pre-specified plan.

Study	Result assessed	Domain	Judgement	Supporting evidence and rationale
Huang (2019)	G3 Pathological / oncological	D1 Confounding	Serious	As above.
Huang (2019)	G3 Pathological / oncological	D2 Classification of interventions	Low	As above.
Huang (2019)	G3 Pathological / oncological	D3 Selection of participants	Moderate	MODERATE for this result. The rectal subgroup is defined by tumour location, a baseline characteristic, and the denominators (77 and 29) are those of all rectal patients, so selection is not post-intervention. The parent cohort is nevertheless a mixed colorectal series.
Huang (2019)	G3 Pathological / oncological	D4 Missing data	Moderate	Pathology reported for all rectal patients.
Huang (2019)	G3 Pathological / oncological	D5 Measurement of outcome	Low	CRM considered positive if cancer cells were within 1 mm (Methods, p. 194); pathological assessment from the specimen.
Huang (2019)	G3 Pathological / oncological	D6 Selection of reported result	Moderate	No pre-specified plan.
Panteleimonitis (2020)	G1 Operative efficiency	D1 Confounding	Serious	The result extracted is unadjusted. A multivariable logistic model adjusting for age, gender, BMI and other clinically relevant variables was applied only to CRM clearance, not to operative time. Platform coincided with centre: every Si case was operated at Portsmouth, May 2013 to February 2016, and every Xi case at Poole, from November 2015, or Lisbon, from May 2016, so platform is collinear with centre and largely with period, and the Xi arm includes training cases at units whose robotic programmes began with the Xi. The centres also differed in Si technique: the Portsmouth centre achieved single-docking fully robotic surgery with the Si by changing port configuration and flipping the arms between stages, whereas the technique at the other two centres is not described.
Panteleimonitis (2020)	G1 Operative efficiency	D2 Classification of interventions	Low	Platform recorded at operation.
Panteleimonitis (2020)	G1 Operative efficiency	D3 Selection of participants	Moderate	All consecutive robotic rectal cancer resections from three prospectively collated databases.
Panteleimonitis (2020)	G1 Operative efficiency	D4 Missing data	Moderate	The paper states that missing values were handled for the binary logistic regression, but the extent of missing data is not quantified.
Panteleimonitis (2020)	G1 Operative efficiency	D5 Measurement of outcome	Moderate	Unblinded but objective; recorded by each centre from theatre records.
Panteleimonitis (2020)	G1 Operative efficiency	D6 Selection of reported result	Moderate	No pre-specified plan.
Panteleimonitis (2020)	G2 Perioperative safety	D1 Confounding	Serious	As above. Length of stay is additionally governed by centre-level discharge policy, and three centres in two countries contribute.
Panteleimonitis (2020)	G2 Perioperative safety	D2 Classification of interventions	Low	As above.

Study	Result assessed	Domain	Judgement	Supporting evidence and rationale
Panteleimonitis (2020)	G2 Perioperative safety	D3 Selection of participants	Moderate	As above.
Panteleimonitis (2020)	G2 Perioperative safety	D4 Missing data	Moderate	As above.
Panteleimonitis (2020)	G2 Perioperative safety	D5 Measurement of outcome	Moderate	Clinically ascertained, unblinded, objective.
Panteleimonitis (2020)	G2 Perioperative safety	D6 Selection of reported result	Moderate	No pre-specified plan.
Panteleimonitis (2020)	G3 Pathological / oncological	D1 Confounding	Serious	As above.
Panteleimonitis (2020)	G3 Pathological / oncological	D2 Classification of interventions	Low	As above.
Panteleimonitis (2020)	G3 Pathological / oncological	D3 Selection of participants	Moderate	As above.
Panteleimonitis (2020)	G3 Pathological / oncological	D4 Missing data	Moderate	As above.
Panteleimonitis (2020)	G3 Pathological / oncological	D5 Measurement of outcome	Low	Pathological assessment of the specimen.
Panteleimonitis (2020)	G3 Pathological / oncological	D6 Selection of reported result	Moderate	No pre-specified plan.
Aliyev (2022)	G1 Operative efficiency	D1 Confounding	Serious	The extracted result is an unadjusted group comparison. The study itself demonstrates the confounder: CUSUM analysis shows the Si learning curve was completed only at the 44th case, and multivariate analysis shows that both the platform and the learning curve were independently associated with console time (Table 3). The Si group was operated from January 2011 (Methods; December 2011 in the Abstract) to December 2017 and the Xi group January 2018 to May 2021, with no overlap. The confounding is therefore documented by the authors and not controlled in the result used here.
Aliyev (2022)	G1 Operative efficiency	D2 Classification of interventions	Low	Platform recorded at operation.
Aliyev (2022)	G1 Operative efficiency	D3 Selection of participants	Moderate	Consecutive sphincter-saving TME cases by a single surgeon.
Aliyev (2022)	G1 Operative efficiency	D4 Missing data	Moderate	Completeness not reported.
Aliyev (2022)	G1 Operative efficiency	D5 Measurement of outcome	Moderate	Unblinded but objective. The Si group required repositioning of the robotic arms between the vascular/mobilisation phase and the pelvic phase whereas the Xi group did not (Methods), but that difference is the causal pathway by which the platform acts, not a difference in how the outcome was measured.

Study	Result assessed	Domain	Judgement	Supporting evidence and rationale
Aliyev (2022)	G1 Operative efficiency	D6 Selection of reported result	Serious	SERIOUS. Table 2 reports a distal margin of 14.4 ± 15.5 mm overall against group means of 19.7 and 16.8 mm, which is arithmetically impossible; the Clavien-Dindo percentages use a denominator of 34 rather than 88 and 62; and several Table 1 rows use a Si denominator of 44 instead of 88. These are demonstrable arithmetic errors in the reported results.
Aliyev (2022)	G2 Perioperative safety	D1 Confounding	Serious	As above.
Aliyev (2022)	G2 Perioperative safety	D2 Classification of interventions	Low	As above.
Aliyev (2022)	G2 Perioperative safety	D3 Selection of participants	Moderate	As above.
Aliyev (2022)	G2 Perioperative safety	D4 Missing data	Moderate	Completeness not reported.
Aliyev (2022)	G2 Perioperative safety	D5 Measurement of outcome	Moderate	The study reports "anastomotic complications", a broader construct than anastomotic leak. The definition is applied identically to both arms, so this is comparable within the study; it is not comparable with the leak definitions used by the other cohorts, which is why this result was excluded from the primary leak analysis by decision D3.
Aliyev (2022)	G2 Perioperative safety	D6 Selection of reported result	Serious	As above.
Aliyev (2022)	G3 Pathological / oncological	D1 Confounding	Serious	As above.
Aliyev (2022)	G3 Pathological / oncological	D2 Classification of interventions	Low	As above.
Aliyev (2022)	G3 Pathological / oncological	D3 Selection of participants	Moderate	As above.
Aliyev (2022)	G3 Pathological / oncological	D4 Missing data	Moderate	Completeness not reported.
Aliyev (2022)	G3 Pathological / oncological	D5 Measurement of outcome	Low	Pathological assessment of the specimen.
Aliyev (2022)	G3 Pathological / oncological	D6 Selection of reported result	Serious	As above. The distal margin result from this study was judged unusable for meta-analysis on the basis of the same arithmetic problem.
Aliyev (2022)	G4 Process measure (diverting stoma)	D1 Confounding	Serious	As above. The population is restricted to sphincter-saving TME for mid-low rectal cancer, so tumour height is relatively homogeneous and stoma policy near-universal in both arms, which limits the scope for confounding of this particular result.
Aliyev (2022)	G4 Process measure (diverting stoma)	D2 Classification of interventions	Low	As above.
Aliyev (2022)	G4 Process measure (diverting stoma)	D3 Selection of participants	Moderate	As above.

Study	Result assessed	Domain	Judgement	Supporting evidence and rationale
Aliyev (2022)	G4 Process measure (diverting stoma)	D4 Missing data	Moderate	Completeness not reported.
Aliyev (2022)	G4 Process measure (diverting stoma)	D5 Measurement of outcome	Low	Objective, from the operative record.
Aliyev (2022)	G4 Process measure (diverting stoma)	D6 Selection of reported result	Serious	As above.
Wang (2026)	G1 Operative efficiency	D1 Confounding	Serious	A multivariable linear regression adjusting for platform and clinical variables was performed, which is more than most cohorts, but calendar period and surgeon experience are not and cannot be included because the Si was used alone from November 2019 to December 2020 and both platforms were available from January 2021, allocation then following availability and theatre scheduling, so platform remains partly collinear with period.
Wang (2026)	G1 Operative efficiency	D2 Classification of interventions	Low	Platform recorded at operation.
Wang (2026)	G1 Operative efficiency	D3 Selection of participants	Moderate	Consecutive institutional series. Possible overlap with the same centre's robotic-versus-laparoscopic proctectomy report over the identical window was noted; that report is not included in this review, so no double counting arises.
Wang (2026)	G1 Operative efficiency	D4 Missing data	Moderate	Completeness not reported.
Wang (2026)	G1 Operative efficiency	D5 Measurement of outcome	Moderate	Unblinded but objective; same timing convention in both arms.
Wang (2026)	G1 Operative efficiency	D6 Selection of reported result	Moderate	No pre-specified plan. Reported results are internally consistent.
Wang (2026)	G2 Perioperative safety	D1 Confounding	Serious	As above.
Wang (2026)	G2 Perioperative safety	D2 Classification of interventions	Low	As above.
Wang (2026)	G2 Perioperative safety	D3 Selection of participants	Moderate	As above.
Wang (2026)	G2 Perioperative safety	D4 Missing data	Moderate	Completeness not reported.
Wang (2026)	G2 Perioperative safety	D5 Measurement of outcome	Moderate	Clinically ascertained, unblinded, objective.
Wang (2026)	G2 Perioperative safety	D6 Selection of reported result	Moderate	No pre-specified plan.
Wang (2026)	G3 Pathological / oncological	D1 Confounding	Serious	As above.

Study	Result assessed	Domain	Judgement	Supporting evidence and rationale
Wang (2026)	G3 Pathological / oncological	D2 Classification of interventions	Low	As above.
Wang (2026)	G3 Pathological / oncological	D3 Selection of participants	Moderate	As above.
Wang (2026)	G3 Pathological / oncological	D4 Missing data	Moderate	Pathology reported for all patients.
Wang (2026)	G3 Pathological / oncological	D5 Measurement of outcome	Low	Pathological assessment of the specimen against standard criteria.
Wang (2026)	G3 Pathological / oncological	D6 Selection of reported result	Moderate	No pre-specified plan.

Supplementary Table 4, panel C. ROBINS-I V2 screening step: whether the result is from a study that attempted to control for confounding, and whether it can be rated at critical risk of bias at screening.

Study	Did the study attempt to control for confounding?	Is confounding by the pre-specified domains so severe that the result cannot be trusted?	Was the intervention assignment affected by the outcome?	Screening outcome
Ozben (2016)	Y - multivariable logistic regression (Tables 5-7) adjusting for operative procedure, (y)pT class and type of mesorectal excision	NA	N	Proceed
Cha (2018)	N - conference abstract; no adjustment, matching or baseline characteristics reported	Y - result is unadjusted, no baseline data are available, and the platforms are period-separated (the Xi became commercially available in April 2014 within a 2011-2017 series)	N	CRITICAL - no further assessment
Morelli (2018)	Y - 1:1 case-matching on age, sex, BMI, ASA, neoadjuvant CRT, stage, distance from anal verge and type of operation	NA	N	Proceed
Tung (2018)	N - no adjustment or matching; baseline limited to age, sex and neoadjuvant treatment	PN - some baseline comparison is reported and the arms are broadly contemporaneous, so the result can be considered further	N	Proceed
Huang (2019)	N - no adjustment; baseline reported and imbalance acknowledged (clinically node-positive 75.8% vs 50%, p=0.002)	PN - baseline characteristics are fully reported, allowing the confounding to be judged rather than merely suspected	N	Proceed
Panteleimonitis (2020)	PY - a multivariable model adjusted for age, gender, BMI and other clinically relevant factors was applied, but only to CRM clearance; the results extracted for this review are unadjusted	PN - baseline characteristics were compared and reported as similar	N	Proceed
Aliyev (2022)	PY - univariate and multivariate analysis performed, but applied to console time and survival; the perioperative results extracted here are unadjusted group comparisons (Table 2)	PN - baseline characteristics are reported	N	Proceed
Wang (2026)	Y - multivariable linear regression for operative time (Table 4) and Cox models adjusting for platform and TNM stage	NA	N	Proceed

Supplementary Table 5. GRADE evidence profile: da Vinci Xi compared with da Vinci Si for rectal cancer surgery, all ten graded outcomes, with the judgement and its reason for every certainty domain.

Outcome	Cohorts / patients (events)	Risk of bias	Incon-sistency	Indirect-ness	Impre-cision	Publication bias	Effect (95% CI)	Anticipated absolute effects	Cer-tainty
**Critical outcomes**									
Anastomotic leak	5 / 449 (22)	Very serious[a]	Not serious[b]	Serious[c]	Serious[d]	Not assessable[e]	RR 0.53 (0.24 to 1.14); ad hoc 0.15 to 1.88	68 per 1000 with the Si; 36 per 1000 with the Xi (17 to 78)	Very low
Overall postoperative complications	5 / 406 (56)	Serious[f]	Not serious[g]	Serious[h]	Serious[d]	Not assessable[e]	RR 0.91 (0.55 to 1.52); ad hoc 0.46 to 1.81	145 per 1000 with the Si; 132 per 1000 with the Xi (79 to 220)	Very low
Conversion to open surgery	5 / 560 (5)	Serious[f]	Not serious[i]	Not serious[j]	Serious[k]	Not assessable[e]	RD +0.07 percentage points (−0.70 to +0.83); ad hoc −1.84 to +1.97	7 per 1000 with the Si; 1 more per 1000 with the Xi (7 fewer to 8 more)	Very low
Reoperation	3 / 353 (16)	Serious[f]	Not serious[i]	Not serious[l]	Very serious[m]	Not assessable[e]	RD +2.6 percentage points (−5.3 to +10.6); ad hoc −7.1 to +12.3	34 per 1000 with the Si; 26 more per 1000 with the Xi (53 fewer to 106 more)	Very low
Circumferential resection margin involvement	6 / 512, 4 informative / 395 (14)	Serious[n]	Not serious[i]	Not serious[o]	Very serious[p]	Not assessable[e]	RR 1.02 (0.23 to 4.49); ad hoc 0.16 to 6.48	28 per 1000 with the Si; 29 per 1000 with the Xi (7 to 126)	Very low
Mesorectal specimen quality, complete or near-complete	4 / 338 (11 incomplete)	Serious[n]	Not serious[i]	Not serious[o]	Serious[q]	Not assessable[e]	RD −0.6 percentage points (−6.2 to +4.9); ad hoc −6.9 to +5.6	970 per 1000 with the Si; 964 per 1000 with the Xi (908 to 1000[r])	Very low
**Important outcomes**									
Operative time	5 / 553	Serious[f]	Serious[s]	Serious[t]	Serious[u]	Not assessable[e]	MD −27.7 min (−80.4 to +25.0); conditional on excluding the largest cohort −50.6 (−69.4 to −31.7)	—	Very low
Docking time	3 / 199	Serious[f]	Not serious[v]	Serious[w]	Serious[x]	Not assessable[e]	Not pooled: −3.10, −6.00 and −2.96 min; 3 of 3 favour the Xi (sign test P = 0.25)	—	Very low
Length of hospital stay	4 / 529	Serious[f]	Serious[y]	Serious[z]	Serious[d]	Not assessable[e]	MD −1.07 days (−3.67 to +1.52)	—	Very low

Outcome	Cohorts / patients (events)	Risk of bias	Incon-sistency	Indirect-ness	Impre-cision	Publication bias	Effect (95% CI)	Anticipated absolute effects	Cer-tainty
Lymph node yield	5 / 589	Serious[f]	Serious[aa]	Not serious[o]	Serious[d]	Not assessable[e]	MD +2.29 nodes (−3.08 to +7.66)	—	Very low

RR risk ratio, RD risk difference, MD mean difference, CI confidence interval. Estimates are random-effects (restricted maximum likelihood) with a Hartung–Knapp interval; the interval with the ad hoc variance correction is given for the dichotomous outcomes, for which τ^2 was estimated as zero.

Applying to every row. Every result was assessed with ROBINS-I, so certainty starts at high and is rated down two levels for serious and three for very serious risk of bias; every other domain is rated down one level for serious and two for very serious. In four cohorts the da Vinci Xi replaced the da Vinci Si within the same team; in one, platform coincided with centre; in one, the platforms were used concurrently only after an initial period of Si use alone; two did not report the timing (Supplementary Table 7). Calendar period, centre, surgeon and team experience and evolving perioperative care are therefore entangled with the platform and could not be adjusted for, and every estimate is of the platform together with those factors. Upgrading was assessed for every outcome and applied to none (Section S1). Risks with the Si are the event rates in all Si patients of every cohort in the primary analysis of the outcome, including cohorts with no event in either arm; risks with the Xi are derived from them and the pooled relative effect or risk difference and were not reported by any study. For every outcome the evidence is very uncertain about the effect of platform.

[a] Risk of bias, very serious. The structural confounding described above, and, in one contributing cohort, leak counts confirmed by its corresponding author (Supplementary Table 4, panel B).

[b] Inconsistency, not serious. Four of five cohorts point in the same direction and the confidence intervals overlap widely; I^2 is 0% (95% CI 0 to 43.1%), which with five cohorts is uninformative and was not read as evidence of consistency.

[c] Indirectness, serious. One contributing cohort ascertained leak by routine contrast enema one month after surgery, detecting subclinical leaks, whereas the others report clinical leaks, so the same label denotes a different event there.

[d] Imprecision, serious. The confidence interval includes both no difference and an appreciable effect in either direction.

[e] Publication bias, not assessable and therefore not rated down. The largest analysis contains five cohorts against a pre-specified threshold of ten; no funnel plot or asymmetry test was produced. This means not assessed, not absent.

[f] Risk of bias, serious. Structural confounding by period, centre or theatre availability; no cohort adjusted, matched or stratified for calendar period, centre or operator experience, and none could have done so where platform was determined by the date of surgery or by centre.

[g] Inconsistency, not serious. The confidence intervals overlap widely; I^2 is 0% (95% CI 0 to 62.6%), which with five cohorts is uninformative.

[h] Indirectness, serious. Four definitions of the complication composite, differing in whether medical as well as surgical events are counted; only one cohort states a 30-day window (Supplementary Table 6).

[i] Inconsistency, not serious. The confidence intervals overlap; I^2 of 0% with three to five cohorts and few events carries little information.

[j] Indirectness, not serious, although definitions of conversion differ between cohorts (Supplementary Table 6).

[k] Imprecision, serious. Five events in 560 patients: the optimal information size is not met, and ratio estimates, which rest on three informative cohorts, range from a fivefold reduction to a tenfold increase. The small absolute difference reflects the rarity of the event, not evidence of equivalence.

[l] Indirectness, not serious. The ascertainment window is 30 days in one cohort, within a 30-day outcome set in one, and not stated in one.

[m] Imprecision, very serious. Sixteen events; the interval spans substantial benefit and substantial harm, and reoperation was more frequent with the Xi in two of three cohorts.

[n] Risk of bias, serious. As in f. The primary analysis excludes the one result at critical risk of bias, in accordance with ROBINS-I guidance; the analysis retaining it is a pre-specified sensitivity analysis.

[o] Indirectness, not serious. The 1 mm threshold for margin involvement, the Quirke grading of the mesorectal specimen and the count of harvested nodes are standard pathological measures applied uniformly.

[p] Imprecision, very serious. Fourteen events; the interval cannot exclude a fourfold relative increase, and margin involvement is the pathological variable most strongly associated with local recurrence.

[q] Imprecision, serious. Eleven incomplete specimens; the optimal information size is not met. The success-scale risk ratio of 0.99 (0.94 to 1.05) looks precise only because more than 95% of specimens were complete.

[r] The derived upper limit computes to 1020 per 1000, which exceeds the attainable maximum, and is shown as 1000.

[s] Inconsistency, serious. I^2 95% (95% CI 92 to 97%); the largest cohort, the only one in which the Xi was slower, points the other way, and the prediction interval spans −154 to +99 minutes.

[t] Indirectness, serious. The cohort carrying most weight in the conditional analysis does not define its timing endpoint and labels the same variable console time elsewhere, and the definitions of the other cohorts differ or are not stated.

[u] Imprecision, serious. The interval of the primary analysis includes no difference and a large reduction.

[v] Inconsistency, not serious. All three cohorts favour the Xi.

[w] Indirectness, serious. A process measure, defined by one cohort only and differently timed in the others, with some procedures requiring two dockings.

[x] Imprecision, serious. Three small cohorts, not pooled; three of three in one direction cannot exclude chance (sign test $P = 0.25$).

[y] Inconsistency, serious. I^2 82% (95% CI 55 to 93%); the prediction interval spans more than ten days.

[z] Indirectness, serious. Discharge practice is determined by institutional and health-system policy rather than by the operating platform.

[aa] Inconsistency, serious. I^2 73% (95% CI 33 to 89%), largely from one cohort whose arms differed in stage and procedure type; the post hoc analysis omitting it is exploratory and was not used for inference.

Supplementary Table 6. Outcome definitions as stated in the index publication of each cohort. Quotation is avoided; each entry paraphrases the source and gives its location. NR not reported; ND reported but not defined.

Cohort	Operative time	Docking and console time	Length of stay	Overall complications	Anastomotic leak	Conversion
Ozben 2016 [21]	Total operative time, first skin incision to final closure of the abdominal wall (Methods)	Docking: from moving the robot into the surgical field to setting the arms into the port sites. Console: time the surgeon operated at the console (Methods). Redocking and a hybrid technique used in some Si cases	ND	Morbidity within 30 days after surgery, including leak, ileus, septic and thromboembolic complications; patients with at least one complication (Methods)	Component of 30-day morbidity; ascertainment not stated	Completion of any part of the procedure open or by standard laparoscopy, excluding specimen delivery and anvil preparation (Methods)
Cha 2018 [22]	Conference abstract; contributes only margin involvement and mesorectal quality, in sensitivity analyses. No definitions reported					
Morelli 2018 [23]	Overall operative time; ND	Docking: ND. Console: NR	Median (range); not pooled	Clavien–Dindo graded; window not stated	Radiological leak on routine water-soluble contrast enema one month after surgery in every patient (Methods)	Conversion to open or hand-assisted laparoscopic surgery; ND
Tung 2018 [24]	ND	NR	Postoperative hospital stay; ND	Early complications; window not stated (operative mortality within 30 days)	Clinical (peritonitis or faecal drain content) or radiological (contrast enema) leakage (Methods); clinical leakage counts used	ND
Huang 2019 [25]	Skin incision to completion of wound closure (Methods); rectal procedures in one sensitivity analysis only	Not contributed	Whole colorectal cohort only; not used	Morbidity within 30 days; whole colorectal cohort only; not used	Clinical or radiological defect of the anastomosis; whole cohort only; not used	Unintended extension of laparotomy beyond the 5-cm retrieval incision, or change to laparoscopy; not used
Panteleimonitis 2020 [26]	Median (IQR); ND. Not recorded at the Portuguese centre, whose 36 patients were all Xi, so 80 Xi and 124	NR	Median (IQR); discharge on meeting discharge criteria within an enhanced	Minor complications (Clavien–Dindo grades 1–2) not recorded (Discussion); no complication composite	Clinical leak requiring re-intervention such as a drain or further surgery; every patient with total mesorectal excision	Any incision needed to mobilise the colon or rectum or to ligate the vessels (Methods)

Cohort	Operative time	Docking and console time	Length of stay	Overall complications	Anastomotic leak	Conversion
	Si patients contribute		recovery programme used at all centres (Methods)	or count of grade III or higher reported by arm; not in the complication analyses	defunctioned (Methods)	
Aliyev 2022 [27]	Operative time in Table 2; the same analysis headed console time in Table 3; ND	Docking: NR (Discussion). Console: see operative time	Mean ± SD; ND	Overall complications; window not stated; Clavien–Dindo percentages computed on a denominator of 34	Anastomotic complications in Table 2, anastomotic leaks in the Results text, same counts; ND; wider-definition sensitivity analysis only	No conversion in either arm; ND
Wang 2026 [28]	First incision to completion of surgical suturing (Methods)	Docking: ND. Console: ND. Dual docking used with the Xi for extended fields (Methods)	Days from completion of surgery to discharge (Methods)	Listed components, including leak and abdominal infection; window not stated	Disruption of the bowel anastomosis with enteric leakage (Methods); ascertainment not stated	NR

Reference numbers are those of the main text. Definitions were extracted from the index publications. Where a cohort contributes no value to an outcome, the entry says so.

Supplementary Table 7. Timing, centres and surgeons of each platform arm, the relation between the arms, co-interventions reported by platform, and possible overlap between cohorts, from the index publication of each cohort.

Cohort	Si arm	Xi arm	Relation of the arms	Centres; surgeons	Co-interventions and technique reported by platform	Possible overlap with another cohort
Ozben 2016 [21]	Jan 2010–Oct 2014 (25)	Nov 2014–Mar 2015 (28)	Sequential; the Xi replaced the Si	One institution, Istanbul (Acıbadem University hospitals); several surgeons	Xi: all fully robotic with single docking. Si: hybrid technique in 10 of 25 and redocking in some. Double-stapled anastomosis in both, hand-sewn coloanal after intersphincteric resection	Si arm overlaps in time and city with the Si arm of Aliyev 2022; author teams differ; neither publication identifies its patients further, so overlap cannot be excluded
Cha 2018 [22]	2011–2017 (55)	2011–2017 (36)	NR	One centre (Lankenau Medical Center); NR	NR	None identified
Morelli 2018 [23]	Apr 2010–Dec 2014 (30)	Jan 2015–Sep 2016 (30)	Sequential; the Xi replaced the Si	One centre (Pisa); one surgeon	Si: single-docking fully robotic or hybrid technique, chosen per patient. Xi: fully robotic with boom rotation. Double-stapled anastomosis and routine contrast enema in both arms	Nine publications of the same series; only the index publication contributes data (Section S4)
Tung 2018 [24]	Jan 2016–Apr 2018 (33, in two arms)	Jan 2016–Apr 2018 (24)	NR	One hospital (Hong Kong); NR	Si arms: hybrid (19) and totally robotic single docking (14). Circular-stapled anastomosis and covering ileostomy in every patient	None identified
Huang 2019 [25]	Dec 2011–May 2015 (77 rectal of 120)	May 2015–Oct 2017 (29 rectal of 60)	Sequential; the Xi replaced the Si	One institution (Taipei); single surgical team	Same inclusion criteria and bowel preparation in both periods; fluorescence imaging mentioned in the Discussion only	None identified
Panteleimonitis 2020 [26]	Portsmouth, May 2013–Feb 2016 (124)	Poole, from Nov 2015, and Lisbon, from May 2016, both to May 2018 (116)	By centre; periods overlap Nov 2015–Feb 2016	Three centres (UK, Portugal); four surgeons, three trained by the senior surgeon with a	Enhanced recovery programme at all centres; defunctioning ileostomy after every total mesorectal excision; integrated table	An interim report of the same cohort is linked to it (Section S4); an Xi-only technical note from the same group was excluded; no overlap with another included cohort identified

Cohort	Si arm	Xi arm	Relation of the arms	Centres; surgeons	Co-interventions and technique reported by platform	Possible overlap with another cohort
				standardised modular technique	motion with the Xi; Xi arm includes training cases	
Aliyev 2022 [27]	Jan 2011 (Methods; Dec 2011 in the Abstract)–Dec 2017 (88)	Jan 2018–May 2021 (62)	Sequential; the Xi replaced the Si	Two hospitals, Istanbul (Liv Hospital; Maslak Acıbadem Hospital); one surgeon, learning curve completed at the 44th case	Double-stapled low anterior resection 75.8% of Xi and 56.8% of Si, the rest intersphincteric with coloanal anastomosis	As for Ozben 2016
Wang 2026 [28]	Nov 2019–Jun 2024 (31)	Jan 2021–Jun 2024 (55)	Si alone to Dec 2020, then both concurrently; allocation by availability and theatre scheduling	One centre (Tianjin); same team, trained before the study	Dual docking with the Xi for extended fields	A robotic-versus-laparoscopic report from the same centre over the same window is not included, so no double counting arises

Periods are recruitment periods as reported, with the arm size in parentheses. NR not reported. Robotic stapling, fluorescence perfusion assessment, total neoadjuvant therapy and transanal total mesorectal excision were not reported by platform in any cohort, and enhanced recovery only in the multicentre cohort. Extracted from the index publications.
